# Photo-thermal Catalytic CO_2_ Methanation by RuO_*x*_@MIL-101(Cr) with 9.2% Apparent Quantum Yield under Visible Light Irradiation

**DOI:** 10.1021/acsami.5c10215

**Published:** 2025-08-25

**Authors:** Juan José Ramírez-Hernández, Vitor Fernandes de Almeida, Zahraa Abou-Khalil, Belén Ferrer, Francesc X. Llabrés i Xamena, Marco Daturi, Guillaume Clet, Ignacio Vayá, Herme G. Baldoví, Amarajothi Dhakshinamoorthy, Mohamad El-Roz, Sergio Navalón

**Affiliations:** † Departamento de Química, 16774 Universitat Politècnica de València, Camino de Vera s/n, Valencia 46022, Spain; ‡ Université de Caen Normandie, ENSICAEN, CNRS, LCS, 14000 Caen, France; § School of Chemistry, Madurai Kamaraj University, Madurai 625021, Tamil Nadu, India; ∥ Instituto de Tecnología Química (CSIC-UPV), Universitat Politècnica de València, Agencia Estatal Consejo Superior de Investigaciones Científicas, Av. de los Naranjos s/n, 46022 Valencia, Spain

**Keywords:** photo-thermal catalysis, metal−organic frameworks, MIL-101(Cr), RuO_*x*_ nanoparticles, CO_2_ methanation

## Abstract

Solar-assisted gaseous CO_2_ hydrogenation to CH_4_ is a potential strategy for favoring the transition to net zero emissions. Here, we report the development of a series of efficient metal–organic frameworks with MIL-101­(Cr or Fe) topology decorated with RuO_*x*_ nanoparticles (ca. 0.2–2 wt %) as heterogeneous photocatalysts for the selective methanation of CO_2_ by H_2_ under simulated sunlight irradiation. The activity of RuO_*x*_(1 wt %)@MIL-101­(Cr) is between 3 and 50 times higher than related MOF-based photocatalysts under similar reaction conditions. Among the different photocatalysts, the optimized RuO_*x*_(2 wt %)@MIL-101­(Cr) photocatalyst showed 98.1% CO_2_ conversion with 98.8% CH_4_ selectivity reaching a production rate of 7.85 mmol g^–1^ h^–1^ with 720 mW cm^–2^ at 200 °C. Further, this photocatalyst exhibited a record apparent quantum yield of 9.2% at 600 nm and 200 °C after subtracting thermal activity contribution compared to any previous MOF- or other heterogeneous-based photocatalyst reported so far. The photocatalyst retained its activity and integrity upon reuse for about 110 h. Transient photocurrent, electrochemical impedance, photoluminescence, and laser flash photolysis spectroscopies together with additional photocatalytic experiments suggest the occurrance of dual photochemical and photothermal reaction pathways. The photocatalytic CO_2_ methanation reaction mechanism was further investigated using *operando* Fourier transform infrared spectroscopy.

## Graphical abstract

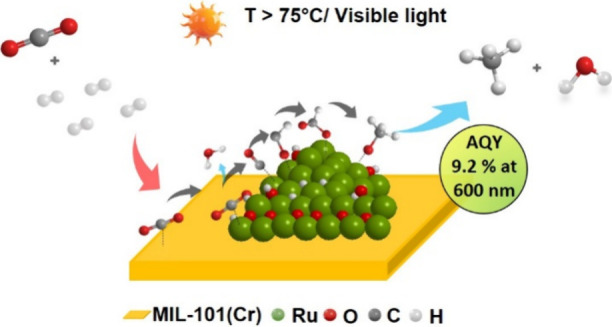

## 1 Introduction

CO_2_ recycling into fuels and chemicals is among the suitable key strategies to achieve net zero emissions and mitigate climate change.^1,2^ In this context, catalysis offers several pathways for converting CO_2_.^2–4^ Some promising catalytic technologies for this purpose include thermocatalysis, electrocatalysis, photocatalysis, and photoelectrocatalysis.^3,5,6^ Despite the need to further improve the efficiencies of these processes, some particular successes have been achieved for thermocatalytic CO_2_ conversion into C1 value-added products such as CO, HCOOH, CH_3_OH, CH_4_, or organic carbonates, among others.^3^

Catalytic CH_4_ production by CO_2_ reduction is considered as one of the potential methods to produce green chemical energy.^7–9^ This synthetic CH_4_, sometimes termed as *synthetic natural gas*, can be directly used in the existing natural gas infrastructure, facilitating its industrial application to help decarbonize industries.^10^ CH_4_ can also be converted into chemicals and fuels such as CH_3_OH, syngas, ethylene, halomethanes, and higher hydrocarbons. Several reviews have investigated catalytic CO_2_ methanation^11,12^ and CH_4_ transformation to value added products.^13^ Catalytic CO_2_ hydrogenation to CH_4_, also known as the Sabatier reaction, is typically carried out at high reaction temperatures (>300–400 °C) and relatively low pressures (<2 bar).^14^ As this process is exothermic, equilibrium is favored at lower reaction temperatures, even though high temperatures can be used to speed up the eight-electron reduction process,^15^ requiring a compromise between thermodynamics and kinetics for efficient industrial applications. In any case, lowering this reaction temperature continues to be a challenge in the field of favoring thermodynamics and reducing external energy requirements.^15^ Importantly, some studies have shown that the photocatalytic version of the Sabatier reaction can achieve higher activities at lower reaction temperatures than the analogous thermocatalytic process.^16^ Some of the reported photocatalysts are based on inorganic semiconductors, such as TiO_2_, perovskites like SrTiO_3_ or BaTiO_3_, and carbon nitride- or graphene-based materials containing metal/metal oxide nanoparticles (NPs) based on ruthenium, palladium, copper, or nickel as cocatalysts. Among these, use of Ru^0^/RuO_*x*_ NPs as cocatalysts provides high activity and selectivity for CO_2_ hydrogenation to CH_4_.^14,15^

Since the first report of using metal–organic frameworks (MOFs) as photocatalysts for CO_2_ reduction, this field has been under continuous development.^17^ MOFs are crystalline porous materials generally built from secondary building units of metal ions, metal–oxo clusters, or chains coordinated to multipodal organic ligands.^18–20^ Some of their unique properties include high versatility and tunability when preparing multifunctional porous (photo)­catalysts with defined energy band level diagrams to meet the thermodynamic requirements of the reactions.^21^ Some of the widely reported MOF-based photocatalysts for CO_2_ reduction in either the liquid or vapor phase in the presence of sacrificial agents (i.e., triethanolamine) include MIL-125­(Ti)-NH_2_,^22^ UiO-66­(Zr)-NH_2_,^23^ MIL-101­(Fe),^24^ MIL-53­(Fe),^24^ MIL-88B­(Fe),^24^ or MIL-101­(Cr),^25,26^ among many others.^27^ In 2019, photocatalytic gaseous CO_2_ hydrogenation to CH_4_ by a Zn-MOF loaded with Cu_2_O NPs was reported for the first time.^28^ Since then, successive studies have reported the development of MOF-based photocatalysts for the same purpose, including MIL-125­(Ti)-NH_2_,^29^ MIP-208­(Ti),^30^ or Zr-MOFs such as multimetallic UiO-66­(Zr/Ce/Ti)^31^ or UiO-66­(Zr/Ti)-X (X= -NH_2_ or -NO_2_)^32^ modified with RuO_*x*_ NPs as cocatalysts. Regardless of the significant achievements made in this field, the possibility of developing MOF-based photocatalysts for the Sabatier reaction using robust chromium- or iron-based MOFs with MIL-101 topology has not yet been reported. MIL-101 solids are built from terephthalate (BDC: 1,4-benzenedicarboxylate) as organic ligands coordinated to trinuclear M_3_-μ_3_-oxo clusters (i.e., M = Cr or Fe) with the theoretical formula [Cr_3_(O)­X­(H_2_O)_2_(BDC)_3_] (X = −OH, −F, −Cl).^33^ Some of the salient properties of these three-dimensional MOFs include high porosity and thermal stability, abundant Lewis acid sites (up to 3.6 mmol g^–1^),^19,33^ and properties that favor a remarkably high CO_2_ adsorption capacity.^19^

Herein, we study the development of MIL-101­(Cr or Fe) solids decorated with RuO_*x*_ NPs as cocatalysts (RuO_*x*_@MIL-101­(Cr)) for selective photo-thermal gaseous CO_2_ hydrogenation to CH_4_ under simulated concentrated solar-light irradiation. The MOFs were prepared by solvothermal methods followed by photodeposition of different loadings of RuO_*x*_ NPs as cocatalysts and characterized by several techniques including powder X-ray diffraction (PXRD), electron microscopy techniques, and spectroscopic and analytical methods. The various samples were evaluated for photocatalytic gaseous CO_2_ reduction with H_2_ under both batch and continuous flow operations. The activity of inorganic semiconductor materials such as Cr_2_O_3_ and γ-Fe_2_O_3_, decorated with RuO_*x*_ NPs, was also considered for comparison. The photocatalytic activity and stability of the most active sample, RuO_*x*_(2 wt %)@MIL-101­(Cr), were studied by reusing the sample several times and characterizing it after use, as well as by investigating any possible deactivation under continuoius flow conditions. Consequently, we obtained insights into the origin of the photocatalytic activity by characterizing the MOFs and, in some cases, Cr_2_O_3_ and γ-Fe_2_O_3_ solids, by spectroscopic techniques including transient photocurrent, (photo)­electrochemical measurements, and spectroscopic techniques such as photoluminescence (PL), time-resolved PL (TR-PL), and laser flash photolysis (LFP). The photocatalytic reaction mechanism of CO_2_ reduction by H_2_ using RuO_*x*_(2 wt %)@MIL-101­(Cr) was also investigated through *operando* FT-IR spectroscopy.

## 2 Methods

Reagents, reactants, detailed experimental procedures for the synthesis of catalysts like MIL-101­(Cr) and MIL-101­(Fe), deposition of RuO_*x*_ NPs, characterization methods employed in this work, photocatalytic experiments under batch conditions, *operando* FT-IR photocatalytic tests, *operando* Raman experiments, and other relevant details are provided in the Supporting Information (SI).

## 3 Results and Discussion

### 3.1 Catalyst Characterization

The comparison of PXRD patterns of the as-synthesized MIL-101­(Cr or Fe) solids with the simulated pattern confirmed the formation of the expected MOF topologies (Figure 1a). Isothermal N_2_ adsorption measurements (SI Figure S1) and thermogravimetric analyses (TGA) (Figure S2) revealed that MIL-101­(Cr) exhibited higher porosity and thermal stability under an air atmosphere (2057 m^2^ g^–1^; 0.99 cm^3^ g^–1^; >300 °C) than MIL-101­(Fe) (1490 m^2^ g^–1^; 0.6 cm^3^ g^–1^; >270 °C). The estimated metal contents of these MOFs from TGA residues at temperatures above 650 °C and associated with Cr_2_O_3_ and γ-Fe_2_O_3_ are in good agreement with the metal content of their theoretical calculation (Figure S2 and Table S1). Attenuated total reflectance FT-IR spectroscopy revealed the presence of carboxylate (υ_asymm_ = 1585 cm^–1^ and υ_symm_ = 1384 cm^–1^) stretching vibrations together with characteristic aromatic C–C sp^2^ (1600 cm^–1^) stretching vibrations in the MOF due to BDC ligands (Figure S3). PXRD measurements of RuO_*x*_@MIL-101­(Cr) (*ca*. 0.2–2 wt %) showed that these solids preserve their initial crystallinity after cocatalyst photodeposition, while MIL-101­(Fe) loses its crystallinity after RuO_*x*_ NPs photodeposition, as revealed by PXRD measurements (Figure 1a). In all cases, the absence of diffraction peaks corresponding to RuO_*x*_ NPs is due to the relatively low loading of Ru (from 0.2 to 2 wt % based on ICP-OES measurements) (Figure 1a) and/or formations of small particles (from 1.25 to 1.56 nm on average for 0.2 and 2 wt % Ru loading, respectively) as revealed by transmission electron microscopy (TEM) measurements (Figures 1c and S4–S8). High-resolution TEM (HR-TEM) analyses on dark spots in the MOF particle revealed a *d*-spacing interlayer distance of about 0.225 nm, recognized to be the 200 facet of RuO_2_ NPs (Figures 1d and S4–S8).^34^ MOFs were also characterized by scanning electron microscopy (SEM) images, revealing the presence of octahedral morphologies for both MIL-101­(Cr) and MIL-101­(Fe) solids with sizes of 192.3 ± 63.1 and 451.4 ± 128.0 nm, respectively (Figures 1b and S9 and S10). The SEM-EDX analyses of different MOF particles confirmed a good distribution of the expected elements within the particles (Figures S11–S17). The relatively low intensities of Ru found in some MOFs were associated with the instrument’s detection limit (*ca*. 1 wt %).

**Figure 1:**
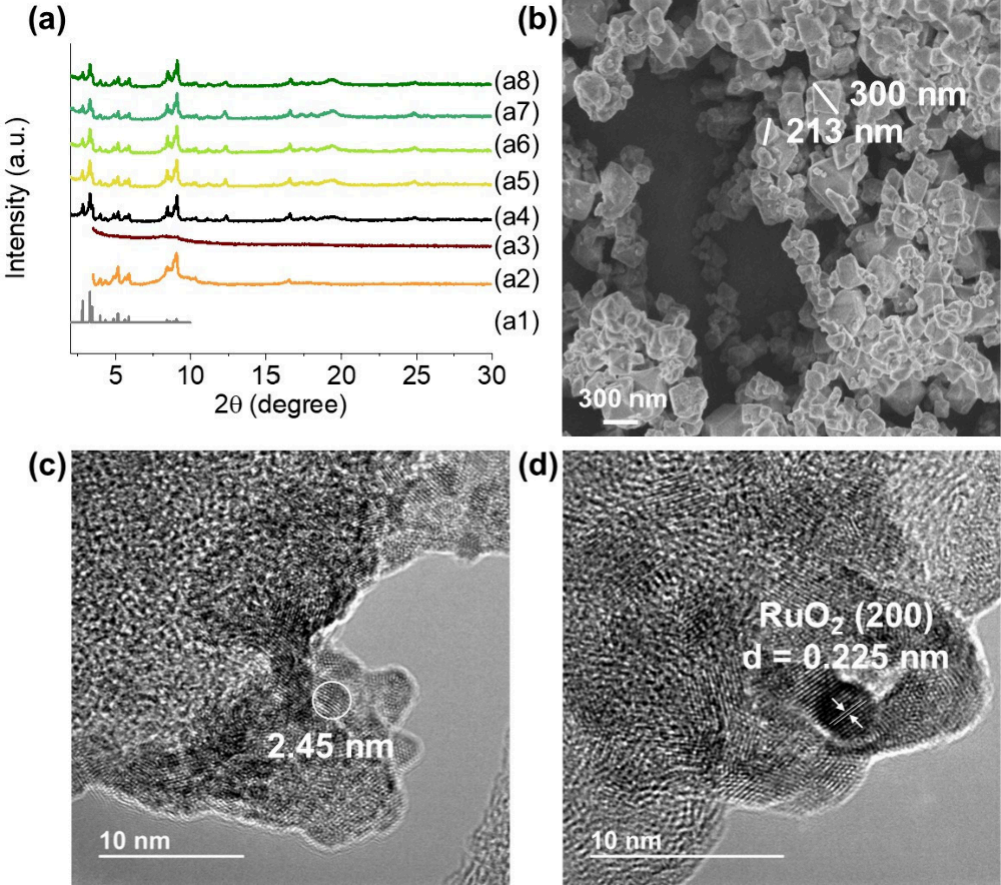
(a) PXRD of (a1) simulated MIL-101, (a2) MIL-101­(Fe), (a3) RuO_*x*_(2 wt %)@MIL-101­(Fe), (a4) MIL-101­(Cr), (a5) RuO_*x*_(0.2 wt %)@MIL-101­(Cr), (a6) RuO_*x*_(0.5 wt %)@MIL-101­(Cr), (a7) RuO_*x*_(1 wt %)@MIL-101­(Cr), and (a8) RuO_*x*_(2 wt %)@MIL-101­(Cr). (b) SEM of MIL-101­(Cr). (c) TEM image of RuO_*x*_(2 wt %)@MIL-101­(Cr) and (d) TEM image of RuO_*x*_ NPs facets in RuO_*x*_(2 wt %)@MIL-101­(Cr).

MIL-101­(Cr or Fe) samples without (Figures S18 and S19) and with RuO_*x*_ NPs (Figures S20–-S24) were further characterized by high-resolution X-ray photoelectron spectroscopy (XPS) (Figure 2). The XPS C 1s spectra showed two bands at 284.4 and 288.1 eV associated with the presence of aromatic sp^2^ carbons and carboxylate groups, respectively, in the MOF organic ligands.^35^ For those containing RuO_*x*_ NPs, XPS of Ru 3d is partially overlapped with C 1s, although a small band centered at 281.5 eV can be assigned to the oxidized Ru species in RuO_*x*_ NPs.^32^ The XPS Ru 3p region is in fact characterized by the presence of two bands at 462.9 and 486.0 eV due to Ru 3 p_3/2_ and Ru 3p_1/2_, which further confirmed the presence of oxidized Ru species. The XPS O 1s spectra are characterized by a broad band due to the partial overlapping of oxygen atoms in the MOF metal nodes or RuO_*x*_ NPs (529.7 eV), together with those present in carboxylate groups (531.7 eV). MIL-101­(Cr)-based solids are characterized by an XPS Cr 2p region, showing two bands at 577.2 and 586.8 eV characteristic of Cr 2 p_3/2_ and Cr 2p_1/2_, respectively, and characteristic of the Cr^3+^ species present in the metal node.^35^ The XPS Fe 2p spectra of the MIL-101­(Fe) solid show two bands at 711.2 and 725.2 eV associated with the Fe 2p_3/2_ and Fe 2p_1/2_ signals of Fe^3+^ in the metal nodes.

**Figure 2:**
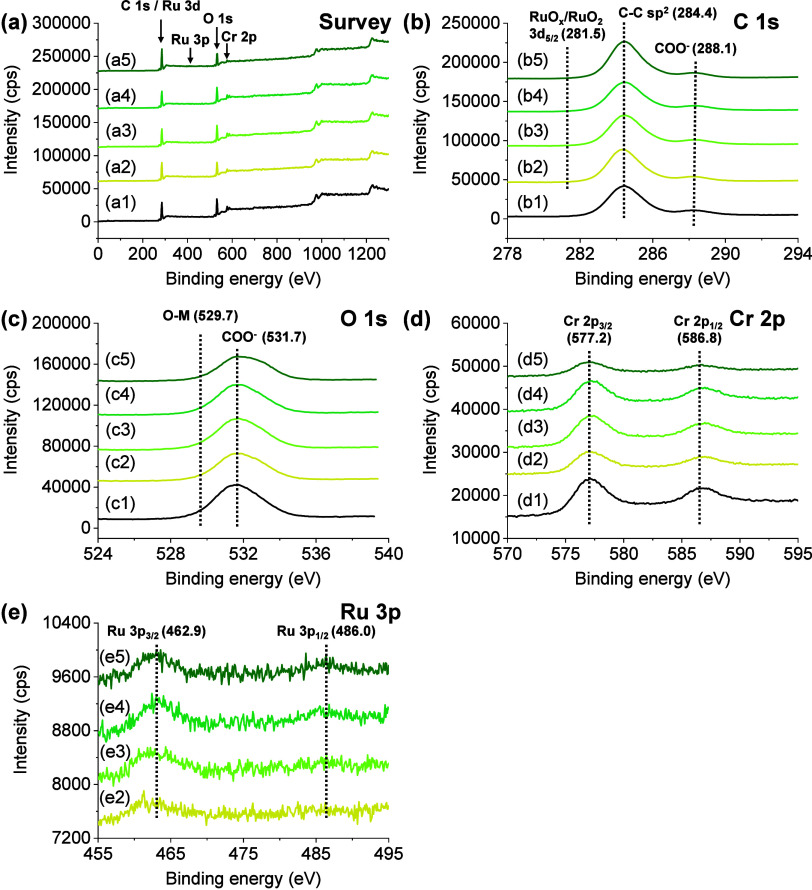
(a) XPS survey, (b) C 1s, (c) O 1s, (d) Cr 2p, and (e) Ru 3p of MIL-101­(Cr) with different RuO_*x*_ loadings. Legend panels: 1, 2, 3, 4, and 5 represent loadings of 0, 0.2, 0.5, 1, and 2 wt % of RuO_*x*_ over MIL-101­(Cr), respectively.

The energy band level diagrams of MIL-101­(Cr or Fe) solids were estimated from the UV–vis diffuse reflectance spectroscopy (DRS) and XPS analyses (Figures S25 and S26).^21^ The UV–vis DRS of MIL-101­(Cr) is characterized by an intense band centered around 250 nm due to the electronic transitions related to the presence of the BDC ligand. The visible absorption bands at 420 and 620 nm are due to the d–d electronic transitions of the Cr^3+^ species in the metal nodes, while the other characteristic Cr^3+^ band at about 300 nm is overlapped with the absorption of the BDC.^36,37^ RuO_*x*_@MIL-101­(Cr) solids showed optical properties similar to those of pristine MOF. It should be noted that the characteristic visible-light absorption from about 400 to 800 nm and due to the localized surface plasmon resonance (LSPR) of RuO_*x*_ NPs is overlapped with the d–d transitions of Cr^3+^ species (Figure 3a).^29,36^ In the case of RuO_*x*_(2 wt %)@MIL-101­(Fe), the UV–vis DRS shows an extra absorption in the range of 500 to 800 nm compared to pristine MIL-101­(Fe) that is due to LSPR of RuO_*x*_ NPs (Figure S26). The optical band gaps of MIL-101­(Cr) and MIL-101­(Fe) estimated from the Tauc plot by the UV–vis DRS data were 2.77 eV and 2.39 eV, respectively. (Figure 3b). The XPS highest occupied crystal orbital (HOCO) maximum band (Figure S25) was estimated to be +1.69 V versus NHE, and the lowest unoccupied crystal orbital (LUCO) minimum calculated from optical band gap and HOCO edge value was −1.08 V versus NHE values, which agrees with previous report using MIL-101­(Cr).^38^ Similarly, the HOCO maximum and LUCO minimum values of MIL-101­(Fe) were also determined (Figure S26). Details on calculating the energy band level diagram can be found in the SI.

**Figure 3:**
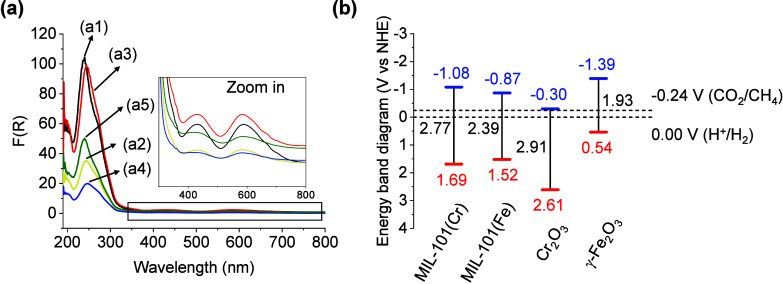
(a) UV–vis DRS of RuO_*x*_@MIL-101­(Cr) solids and (b) estimated energy band level diagrams of the photocatalysts under study. Legend panels: (a1), (a2), (a3), (a4), and (a5) represent loadings of 0, 0.2, 0.5, 1, and 2 wt % of RuO_*x*_ NPs over MIL-101­(Cr), respectively.

Commercially available Cr_2_O_3_ (2.1 m^2^ g^–1^; particles of 458.8 ± 200.6 nm from Figure S27) and γ-Fe_2_O_3_ (50–245 m^2^ g^–1^; particles of 36.9 ± 13.8 nm from Figure S28) were included in the study for comparison. The XRD of Cr_2_O_3_ and γ-Fe_2_O_3_ with and without RuO_*x*_ NPs (2 wt % Ru as evidenced by ICP-OES analyses) can be found in SI Figure S29. TEM analyses of RuO_*x*_(2 wt %)@Cr_2_O_3_ (Figure S30) and RuO_*x*_(2 wt %)@γ-Fe_2_O_3_ (Figure S31) allow estimating the average and standard deviation values of RuO_*x*_ NPs being 1.79 ± 0.49 and 1.56 ± 0.53 nm, respectively. In both cases, HR-TEM measurements allowed us to determine interplanar distances of about 0.225 nm that agree with the presence of (200) facets in RuO_2_. SEM-EDX analyses revealed a good distribution of the expected elements within the MOF particles (Figures S32–S35). The energy band level diagrams of Cr_2_O_3_ and γ-Fe_2_O_3_ solids (Figures S36 and S37) determined as described for MIL-101 solids confirm that they meet the thermodynamics for CO_2_ photoreduction by H_2_ (Figure 3b).

### 3.2 Photoassisted Catalytic Activity

The catalytic performance of MIL-101­(Cr or Fe) samples with or without RuO_*x*_ NPs was evaluated in the photoassited gaseous CO_2_ hydrogenation reaction at 200 °C under simulated concentrated sunlight irradiation (420 mW cm^–2^). In general, all the experiments show the formation of CH_4_ as main product. After 22 h of reaction, CH_4_ production was only 11.9, 8.8, 11.3, and 10.2 μmol g^–1^ over MIL-101­(Cr), MIL-101­(Fe), Cr_2_O_3_, γ-Fe_2_O_3_, respectively. In sharp contrast, RuO_*x*_ NPs (2 wt %) supported on MIL-101­(Cr) provided the highest photoassisted catalytic activity with CO_2_ conversion of ∼92% to CH_4_ (35.2 mmol g^–1^) with 98% selectivity along with the formation of ethane (1.49%), propane (0.51%), and CO traces under similar reaction conditions. An analogous photocatalytic control experiment with labeled ^13^CO_2_ was performed and confirmed the formation of ^13^CH_4_ with *m*/*z* = 17 and its characteristic low mass fragments as evidenced by gas-phase analysis using a gas chromatograph coupled to a mass spectrometer (GC-MS) (Figure S38). Besides, a decrease of RuO_*x*_ NPs loading on MIL-101­(Cr) from 2 to 0.2 wt % resulted in a gradual drop in the CH_4_ production, which highlights the important role of these NPs as cocatalysts (Figure 4a). Although RuO_*x*_(2 wt %)@MIL-101­(Fe) contains small-size RuO_*x*_ NPs (1.70 ± 0.52 nm), its negligible activity is likely due to MOF structural collapse during RuO_*x*_ NPs deposition (Figure 1a). To rationalize the possible benefits of MOFs, analogous control experiments were performed with RuO_*x*_(2 wt %) NPs supported on Cr_2_O_3_ or γ-Fe_2_O_3_. Under identical experimental conditions, CH_4_ production was 18.21 and 3.70 mmol g^–1^ after 22 h with RuO_*x*_(2 wt %)@Cr_2_O_3_ and RuO_*x*_(2 wt %)@γ-Fe_2_O_3_, respectively (Figure 4b). These inferior performances indicate the benefits of using stable MOFs like MIL-101­(Cr) as photocatalysts over these metal oxides.

**Figure 4:**
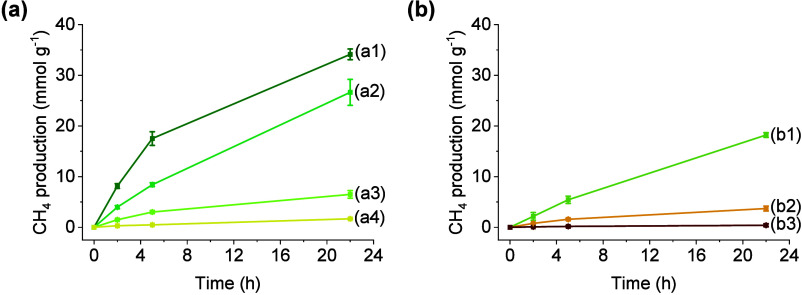
(a) Photoassisted catalytic CO_2_ methanation using MIL-101­(Cr) solid loaded with RuO_*x*_ NPs at (a1) 2, (a2) 1, (a3) 0.5, and (a4) 0.2 wt %. (b) Photocatalytic CO_2_ methanation using RuO_*x*_ NPs (2 wt %) supported on (b1) Cr_2_O_3_, (b2) γ-Fe_2_O_3_, or (b3) MIL-101­(Fe). Reaction conditions: Photocatalyst (15 mg), *P*_H_2__ = 1.2 bar, *P*_CO_2__ = 0.3 bar, and simulated concentrated sunlight irradiation (420 mW cm^–2^), 200 °C.

The influence of the reaction temperature during photoassisted catalytic CO_2_ methanation was studied with RuO_*x*_(2 wt %)@MIL-101­(Cr) under concentrated simulated sunlight irradiation (420 or 720 mW cm^–2^) (Figures 5a,b) or under dark conditions (Figure 5c). In general, the catalytic activities were about 60–70% lower (Figure 5c) under dark conditions than to those photoassisted. As will be shown in Section 3.3, irradiances at 420 and 720 mW cm^–2^ correspond to reaction pathways related to photochemical and photothermal pathways, respectively. During the photochemical reaction pathway, light energy is mainly transformed into charge carriers (electrons and holes), while during the photoassisted thermal mechanism, solar light is transformed into heat. Both processes can promote reduction of CO_2_ by H_2_. It should be noted that the photocatalytic experiment performed at 720 mW cm^–2^ achieves a 98.1% CO_2_ conversion with CH_4_ selectivity of 98.8% accompanied by ethane (1.1%) and propane (0.1%) together with CO traces after 5 h at 200 °C. As will be shown in Section 3.3, these results are interpreted due to the occurrence of a dual photochemical and photothermal reaction pathway. It should be emphasized that the highest CH_4_ production (36.2 mmol g^–1^) is achieved with concentrated sunlight irradiance at 720 mW cm^–2^ after 5 h, as well as performing low-temperature photomethanation at 175 and 125 °C, with values of 15.7 and 2.4 mmol g^–1^ in 5 h, respectively.

**Figure 5:**
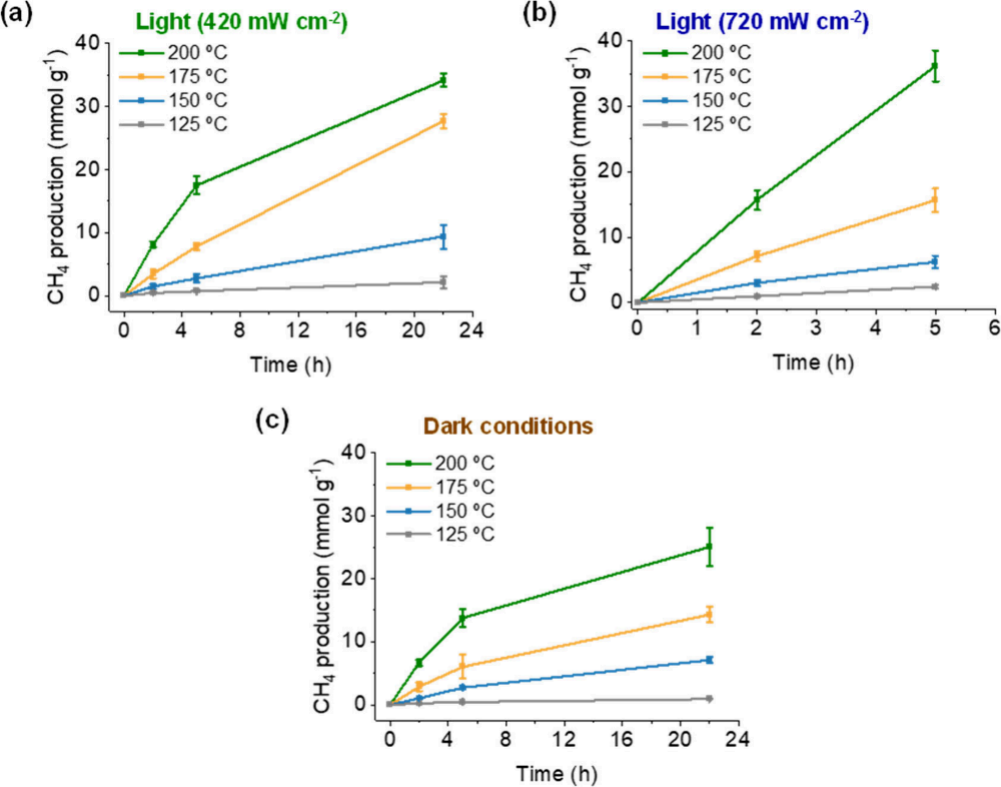
(a) Influence of reaction temperature on the photoassisted catalytic CH_4_ generation using RuO_*x*_(2 wt %)@MIL-101­(Cr) under concentrated simulated sunlight irradiation at 420 mW cm^–2^ and (b) 720 mW cm^–2^ and (c) under dark conditions. Reaction conditions: RuO_*x*_(2 wt %)@MIL-101­(Cr) (15 mg), *P*_H_2__ = 1.2 bar, *P*_CO_2__ = 0.3 bar, simulated concentrated sunlight irradiation, and temperature as indicated.

Furthermore, the performances of RuO_*x*_(2 wt %)@MIL-101­(Cr) and MIL-101­(Cr) under flow operation conditions (H_2_:CO_2_ molar ratio of 4:1; total flow rate of 10 cm^3^ min^–1^) were systematically evaluated by using a sandwich-cell reactor (Scheme S1) under visible-light irradiation (λ > 390 nm) at reaction temperatures ranging from 30 to 200 °C (Section S4.2 in the SI). MIL-101­(Cr) showed negligible activity, producing only small amounts of CO (0.198 mmol g^–1^ h^–1^) at 200 °C, irrespective of illumination. Conversely, RuO_*x*_(2 wt %)@MIL-101­(Cr) commenced CH_4_ generation at 75 °C and increased with temperature under dark or visible-light conditions (Figure 6a). A pronounced enhancement in CH_4_ production was found under visible-light irradiation, reaching 9.4 mmol g^–1^ h^–1^ at 200 °C with a 95% selectivity towards CH_4_. CO, ethane, and propane were also identified as other products with selectivities of 1.5%, 2.5%, and 1%, respectively (inset in Figure 6a). Online MS measurements further demonstrated the production of H_2_O as another main product, as shown in Figure 6b. These findings highlight the synergistic effect of RuO_*x*_ NPs and visible-light irradiation in promoting CO_2_ methanation with a high selectivity.

**Figure 6:**
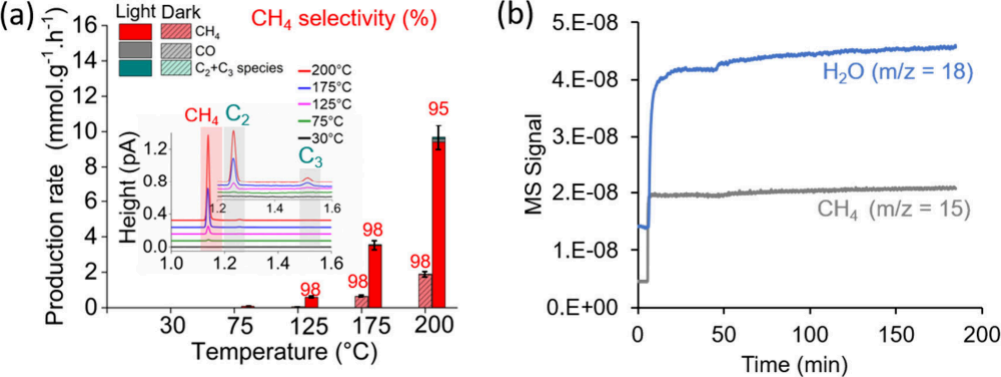
(a) Activity and selectivity of RuO_*x*_(2 wt %)@MIL-101­(Cr) as a function of temperature in dark and under visible light irradiation under flow conditions. The inset shows gas chromatograms of the reaction gas phase under the same conditions. (b) MS evolution of CH_4_ and H_2_O during the CO_2_ photomethanation reaction at 200 °C with RuO_*x*_(2 wt %)@MIL-101­(Cr) under flow conditions. Reaction conditions: RuO_*x*_(2 wt %)@MIL-101­(Cr) (20 mg), total pressure (1 bar), CO_2_:H_2_ (2:8 cm^3^ min^–1^) gas mixture, and visible light irradiation (>390 nm; 70 mW cm^–2^).

The achieved photocatalytic activities of RuO_*x*_(1 wt %)@MIL-101­(Cr) were compared to those of other previously reported MOF-based materials (Figure 7a and Table S2). Figure 7a shows that RuO_*x*_(1 wt %)@MIL-101­(Cr) exhibits a record activity compared to related MOF-based photocatalysts under similar reaction conditions (Table S2, entries 1–9). Specifically, this photocatalyst is about 3 and 50 times higher than to RuO_*x*_(1 wt %)@MIL-125­(Ti)-NH_2_^29^ and RuO_*x*_(1 wt %)@UiO-66­(Zr/Ti)-NO_2_,^32^ respectively. It should be noted that caution should be given when comparing photocatalytic activities, since there may be large differences in the photoreactor setup, including reactor design, reaction temperature, light sources, and corresponding irradiances, or the nature of cocatalyst (e.g., composition, particle size distribution, and loading) among others (Table S2, entries 10 and 11). The use of RuO_*x*_(2 wt %)@MIL-101­(Cr) as photocatalyst at 200 °C further increases CH_4_ production to 4.07 and 7.85 mmol g^–1^ h^–1^ working at 420 and 720 mW cm^–2^, respectively (Table S2, entries 12 and 13). These activities are much better compared to a high Ru loaded RuO_*x*_(10 wt %)@MIL-125­(Ti)-NH_2_ (0.98 mmol g^–1^ h^–1^) even working at 1120 mW cm^–2^ and 200 °C (Table S2, entry 14).

**Figure 7:**
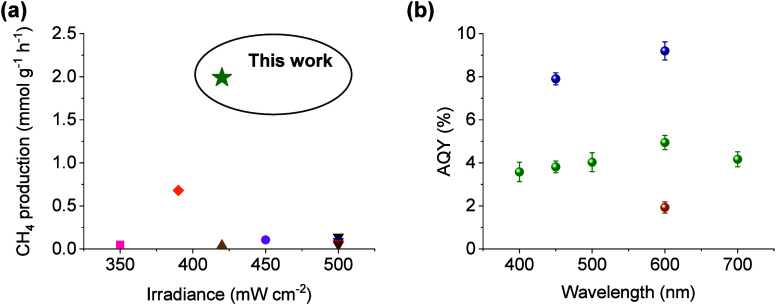
(a) Comparison of photocatalytic CH_4_ production from gaseous CO_2_ hydrogenation as a function of simulated sunlight irradiance at 200 °C using RuO_*x*_(1 wt %)@MIL-101­(Cr) (★, green color, this work) and previously reported MOF-based photocatalysts. Legend: RuO_*x*_(1 wt %)@UiO-66­(Zr/Ti)-NO_2_ (⧫, orange color),^32^ RuO_*x*_(1 wt %)@UiO-66­(Zr/Ce/Ti) (■, pink color; ●, violet color; ▼, black color),^31^ RuO_*x*_(1 wt %)@UiO-66­(Zr/Ti) (▼, blue color),^31^ RuO_*x*_(1 wt %)@UiO-66­(Zr/Ce) (▼, red color),^31^ RuO_*x*_(1 wt %)@UiO-66­(Ce) (▼, light blue color),^31^ RuO_*x*_(1 wt %)@UiO-66­(Zr) (▼, brown color),^31^ RuO_*x*_(1 wt %)@MIL-125­(Ti)-NH_2_ (▲, light brown color).^29^ (b) AQY results of RuO_*x*_(2 wt %)@MIL-101­(Cr). Legend: as a function of the wavelengths and light intensities at 200 °C. Orange, green, and blue balls refer to experiments carried out at 10, 30, and 40 mW cm^–2^, respectively.

The apparent quantum yield (AQY) is a parameter considered to be more appropriate than the product production rate (e.g., mmol g^–1^) to compare photocatalytic activities, taking into account the nature of irradiance.^39^ This parameter correlates the number of reacted electrons with the number of incident photons in a specific wavelength with respect to the activity observed under dark conditions. In general, the increase of activity observed due to photocatalyst irradiation with photons with respect to dark conditions can be associated with different reaction pathways,^40–42^ including the following: (1) photochemical pathway wherein photons are transformed into electrons and holes; (2) photothermal pathway wherein photons are transformed into local heat; (3) a combination of both photochemical and photothermal pathways. Regardless of the operation of the photocatalytic reaction mechanism, the efficiency of the whole process can be quantified by AQY parameter after subtracting thermal activity contribution (results in dark).^40,41^ In this work, the efficiency of RuO_*x*_(2 wt %)@MIL-101­(Cr) for CH_4_ generation at visible wavelength was thus assessed by subtracting its generation under dark conditions from analogous experiments under monochromatic irradiation at 200 °C. The achieved AQYs in the range between 400 to 700 nm are around 4% (Figure 7b) when using an irradiation power of about 30 mW cm^–2^, while an AQY of about 9.2% at 600 nm was achieved under irradiation at about 40 mW cm^–2^. These results are much higher than previous reports using MOF-based photocatalysts. For example, a recent study with RuO_*x*_(1 wt %)@UiO-66­(Zr/Ti)-NO_2_ as a MOF-based photocatalyst working at 200 °C achieved AQYs of 0.25 and 0.01% upon irradiation at 400 and 600 nm, respectively.^32^ As far as we know, one of the most active heterogeneous photocatalysts reported until now under visible-light irradiation is a Rh/TiO_2_ with the maximum AQY value of about 7.2% using a blue LED (450 nm) at 250 °C.^43^ In another work, Cu_2_O NPs supported on graphene-based material exhibited an AQY 7.84% in the range of 250 – 360 nm at 250 °C.^40^ Further, Ni NPs supported carbon nitride showed an AQY of 0.01% at 150 °C using UV–vis light irradiation.^44^ Regardless of the variations in photocatalytic setups and illumination conditions, it appears, to the best of our knowledge, that the achieved AQY using RuO_*x*_(2 wt %)@MIL-101­(Cr) at 200 °C in the visible region is the highest reported so far.

Reusability of RuO_*x*_(2 wt %)@MIL-101­(Cr) solid was evaluated by performing consecutive photocatalytic experiments. The results showed that this photocatalyst retained >90% of its initial photocatalytic activity. A comparison of the PXRD patterns of fresh and five times-used photocatalyst does not show any significant differences, showing that the structural integrity and crystallinity are retained under these experimental conditions (Figure 8b). Although SEM (Figures 8c and S39) characterization of the five times used sample does not show significant changes in MOF morphology, some RuO_*x*_ NP aggregation (1.89 ± 0.50 nm) is seen with respect to a pristine sample (1.56 ± 0.51 nm) as shown by TEM images (Figures 8d and S40). The XPS characterization of the used sample showed that RuO_*x*_ NPs are partially reduced to metallic Ru^0^, especially evidenced by XPS Ru 3p signals of the used photocatalyst that are shifted to lower binding energy values (ca. 0.7 eV) (Figures 8d,e and S41 and S42). To further address the partial reduction of RuO_*x*_@MIL-101­(Cr) under similar reductive reaction conditions, an *in situ* XPS comparison experiment of RuO_*x*_(2 wt %)@MIL-101­(Cr) whether or not submitted to a thermal H_2_ treatment at 200 °C was carried out (Figures S43–S45). The results indicated that RuO_*x*_ NPs are partially reduced to metallic Ru^0^, as shown by the negative binding energy shifts of Ru 3d and 3p of about 1.2 and 0.4 eV, respectively (Figure S45). In contrast, the binding energies of C 1s, O 1s, and Cr 3p of fresh and used or H_2_ treated sample are practically similar, indicating that the oxidation state of these elements is not modified during photocatalytic experiments of reductive *in situ* XPS measurements. It should be noted that previous related studies proposed that metallic Ru species are active sites for H_2_ activation, while those with oxidized Ru can favor CO_2_ and CO chemisorption,^45,46^ so that both species together play a role in the CO_2_ methanation.

**Figure 8:**
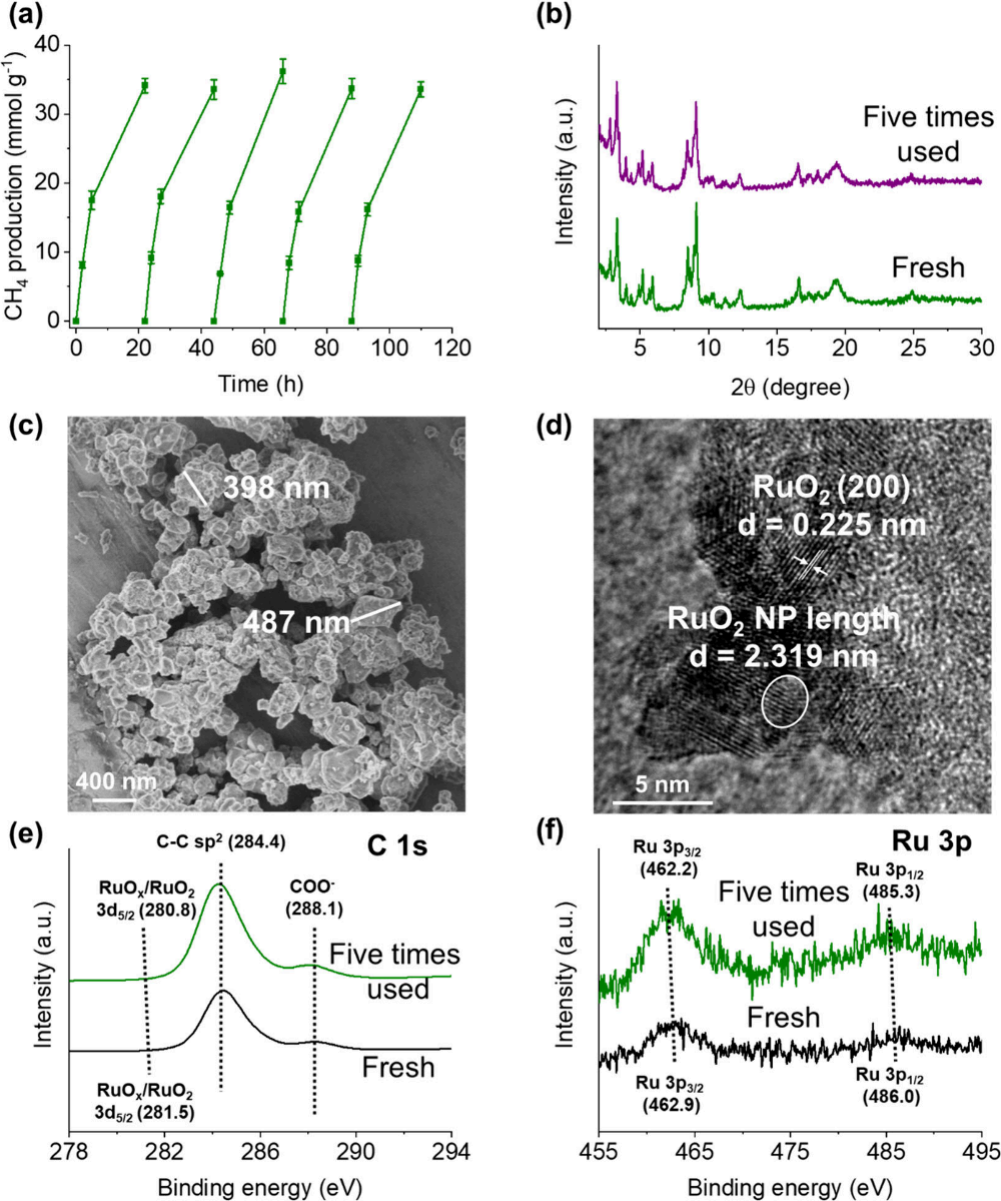
(a) Photocatalytic reusability of RuO_*x*_(2 wt %)@MIL-101­(Cr), (b) its corresponding PXRD pattern before and after five times use, (c) SEM and (d) TEM images of five times used RuO_*x*_(2 wt %)@MIL-101­(Cr) solid, and (e) XPS of C 1s and (f) XPS of Ru 3p. Reaction conditions: RuO_*x*_(2 wt %)@MIL-101­(Cr) (15 mg), *P*_H_2__ = 1.2 bar, *P*_CO_2__ = 0.3 bar, simulated concentrated sunlight irradiation (420 mW cm^–2^), and 200 °C.

### 3.3 Evaluation of Light Influence on Photoassisted Catalytic Reaction Pathways

This section is devoted to evaluating the role of light during the photocatalytic process using an MIL-101­(Cr) solid both loaded and unloaded with RuO_*x*_ NPs. As will be discussed later, a dual photochemical and photothermal reaction mechanism is proposed in this work. Incident photons on the photocatalyst through photochemical pathway generate electrons and holes that can promote reduction and oxidation reactions, respectively.^21^ Subsequently, photogenerated holes are consumed by H_2_ molecules that are oxidized into protons, while CO_2_ is reduced by photogenerated electrons and protons.^47,48^ This process is similar to the photocatalytic CO_2_ reduction in the presence of H_2_O, where H_2_O is oxidized to O_2_ and releases protons.^49^ Some of the advantages reported while using H_2_ instead of H_2_O as oxidizable substrate during the photocatalytic process is the lower oxidation potential of the former that boosts the overall efficiency of the process.^50^ In this context, a photocatalytic control experiment performed using CO_2_ (0.3 bar), Ar (1.2 bar), and H_2_O (20 mL; 1.1 mmol) in the presence of RuO_*x*_(2 wt %)@MIL-101­(Cr) at 200 °C under simulated concentrated sunlight irradiation (420 mW cm^–2^) resulted in a CH_4_ production of 0.01 mmol g^–1^ h^–1^ after 22 h. This activity is significantly much lower than the activity achieved with the photocatalytic CO_2_ (0.3 bar) reduction in the presence of H_2_ (1.2 bar) involving RuO_*x*_(2 wt %)@MIL-101­(Cr) as a photocatalyst at 200 °C resulted in a CH_4_ production of 4.07 mmol g^–1^ h^–1^ after 22 h. In addition, it will be shown that incident photons are transformed into heat and thus promoting a photothermal mechanism.^51^ As commented above, RuO_*x*_ NPs supported on fresh photocatalysts are partially reduced during the reaction, as shown by characterization of the used samples and quasi *in situ* XPS measurements under a reductive atmosphere. To consider the changes that occur in the RuO_*x*_ species during the photocatalytic reaction, the following characterizations were performed with the used samples: transient photocurrent, EIS, PL, and TR-PL. The samples modified with RuO_*x*_ NPs with the highest (2 wt %) and lowest (0.2 wt %) Ru loadings were studied and the results compared to those from a pristine MIL-101­(Cr) solid.

Transient photocurrent and EIS measurements were carried out to get insights into photoinduced charge separation efficiencies and photocatalytic activities using MOF-based solids. The transient photocurrent results upon working electrode (WE) polarization at +0.9 V (Figure 9a) show that the order of current intensity agrees with the order of photocatalytic activity (Figure 4). The higher the RuO_*x*_ NPs loading within MIL-101­(Cr), the higher the current intensity response on irradiation. To gain some insights about the role of H_2_ as an oxidizable substrate during photocatalytic CO_2_ hydrogenation, additional transient photocurrent measurements were performed without applying any WE polarization and in the absence or presence of H_2_. In contrast to the experiment performed using pristine MIL-101­(Cr), an increase of current intensity is observed in the case of used RuO_*x*_@MIL-101­(Cr) as WE also in the presence of H_2_. These experiments highlight the role of supported RuO_*x*_ NPs to adsorb H_2_,^48^ which is subsequently oxidized by photogenerated holes, minimizing electron/hole pair recombination and leading to an increase of photocurrent intensity.^48^ Besides, EIS studies using Nyquist plots were carried out to estimate the charge transfer resistance of MIL-101­(Cr) solids both loaded and unloaded with RuO_*x*_ NPs (Figure 9b). These Nyquist plots were modeled by a simplified Randles circuit and details can be found in the SI. EIS results (Table S3) agree with those of transient photocurrent: lower charge transfer resistances (*R*_ct_) of these solids were achieved with increasing amounts of RuO_*x*_ NPs. Specifically, estimated *R*_ct_ values of 1971, 4392, and 5054 Ω were obtained for RuO_*x*_(2 wt %)@MIL-101­(Cr), RuO_*x*_(0.2 wt %)@MIL-101­(Cr), or MIL-101­(Cr), respectively. RuO_*x*_@MIL-101­(Cr) irradiation further reduces the *R*_ct_ values to about 1550 Ω, in agreement with the role of light in promoting photoinduced charge generation. In line with previous transient photocurrent experiments, additional EIS measurements were performed with pristine MIL-101­(Cr) and used RuO_*x*_@MIL-101­(Cr) as WE, without polarization, under dark conditions in the presence of H_2_. In the case of pristine MIL-101­(Cr), these results revealed that the presence of H_2_ during these measurements does not modify the charge transfer resistance. In contrast, the Nyquist plot recorded with used RuO_*x*_@MIL-101­(Cr) in the presence of H_2_ shows a smaller arc radius that indicates reduced charge transfer resistance with respect to an analogous experiment in the absence of H_2_. These observations are attributed to H_2_ storage within supported RuO_*x*_ NPs, likely as ruthenium/hydride-like species (RuO_*x*_–H).^48,52,53^ Overall, the transient photocurrent and EIS results agree with the role of RuO_*x*_ NPs, not only as a cocatalyst for CO_2_ reduction but also as a counterpart to improve the efficiency of photoinduced charge separation.

**Figure 9:**
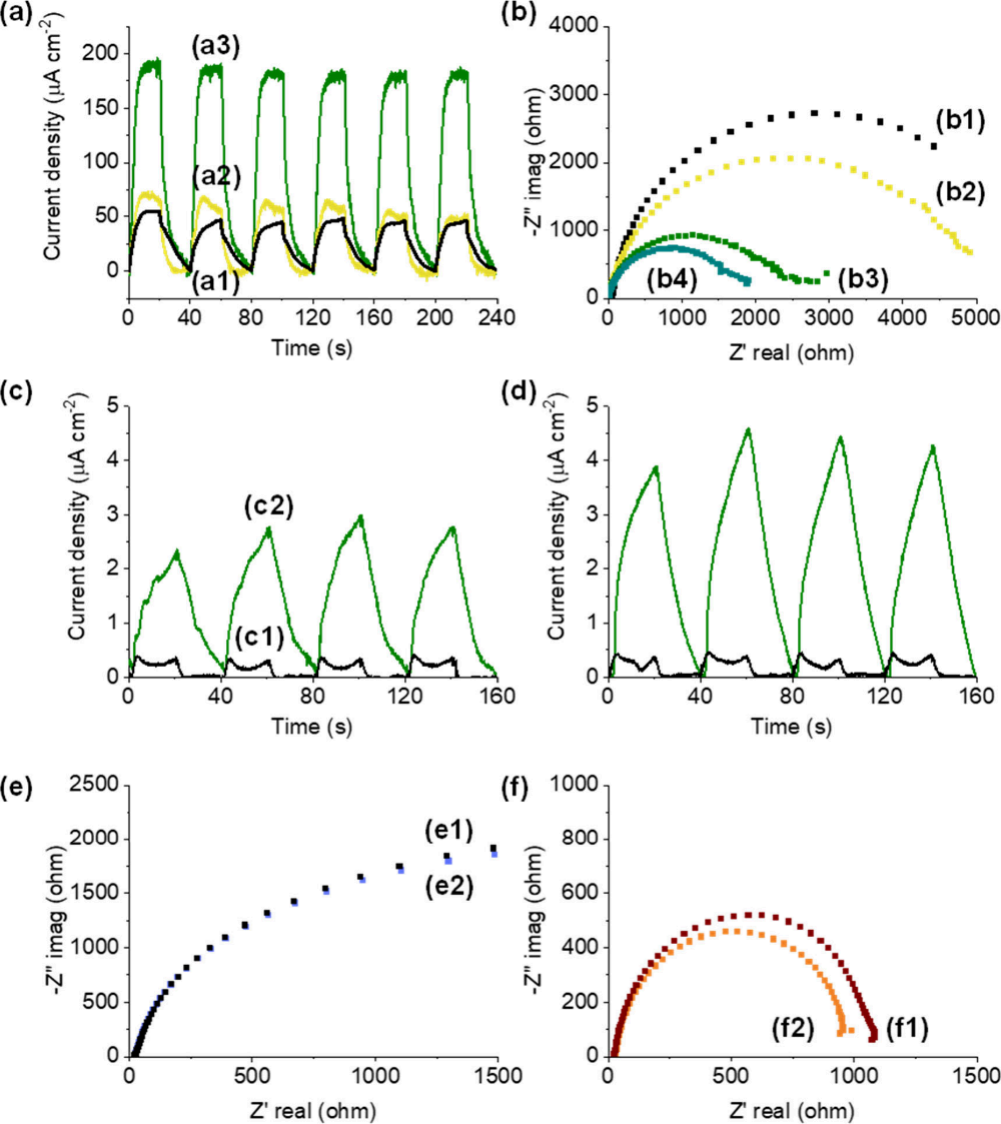
(a) Transient photocurrent response with WE polarized at +0.9 V for (a1) MIL-101­(Cr), (a2) RuO_*x*_(0.2 wt %)@MIL-101­(Cr), and (a3) RuO_*x*_(2 wt %)@MIL-101­(Cr) and (b) Nyquist plots with WE polarized at +0.2 V for (b1) MIL-101­(Cr), (b2) RuO_*x*_(0.2 wt %)@MIL-101­(Cr), (b3) RuO_*x*_(2 wt %)@MIL-101­(Cr), and (b4) RuO_*x*_(2 wt %)@MIL-101­(Cr) under simulated sunlight irradiation. (c, d) Transient photocurrent employing MIL-101­(Cr) (black line) and used RuO_*x*_(2 wt %)@MIL-101­(Cr) (green line) as WE without WE polarization in the absence (c) and presence (d) of H_2_. (e) Nyquist plots of MIL-101­(Cr) as a WE and without polarization in the absence (e1) and presence (e2) of H_2_. (f) Nyquist plots of used RuO_*x*_(2 wt %)­MIL-101­(Cr) as WE and without polarization in the absence (f1) and presence (f2) of H_2_.

Nanosecond LFP measurements were carried out to investigate the nature of the photogenerated charge carriers of the solids. For this purpose, Ar-purged acetonitrile suspensions with absorbances ca. 0.35 at the excitation wavelength (λ_exc_ = 266 nm) were prepared. This wavelength was selected to excite the organic BDC ligand of the MOFs. The obtained spectrum for RuO_*x*_(2 wt %)@MIL-101­(Cr) (Figure 10a), RuO_*x*_(0.2 wt %)@MIL-101­(Cr) (Figure S46a), or MIL-101­(Cr) (Figure S46b) under Ar on the nanosecond time scale is characterized by continuous absorption from 340 to 750 nm. To obtain key information about the nature of the observed transient signals, CH_3_OH and O_2_ were employed as hole and electron quenchers, respectively. In the case of the RuO_*x*_(2 wt %)@MIL-101­(Cr) and RuO_*x*_(0.2 wt %)@MIL-101­(Cr) samples, the presence of CH_3_OH resulted in a partial quenching of the signals from about 650 to 800 nm, accompanied by an increase in the absorption intensities of the signals from 340 to 650 nm (Figure 10a). These findings suggest that hole deactivation by CH_3_OH occurred simultaneously with a concomitant increase of electrons. In addition, O_2_ also quenches the transient absorption signals around 450 and 550 nm but to a lesser extent. For RuO_*x*_(0.2 wt %)@MIL-101­(Cr) (Figure S46a) and pristine MIL-101­(Cr) (Figure S46b), quenching of the absorption species was also detected in the presence of CH_3_OH or O_2_ but with a much lower yield than for the RuO_*x*_(2 wt %)@MIL-101­(Cr) solid (Figure 10a), again indicating the role of RuO_*x*_ NPs in stabilizing photogenerated electrons and holes.

**Figure 10:**
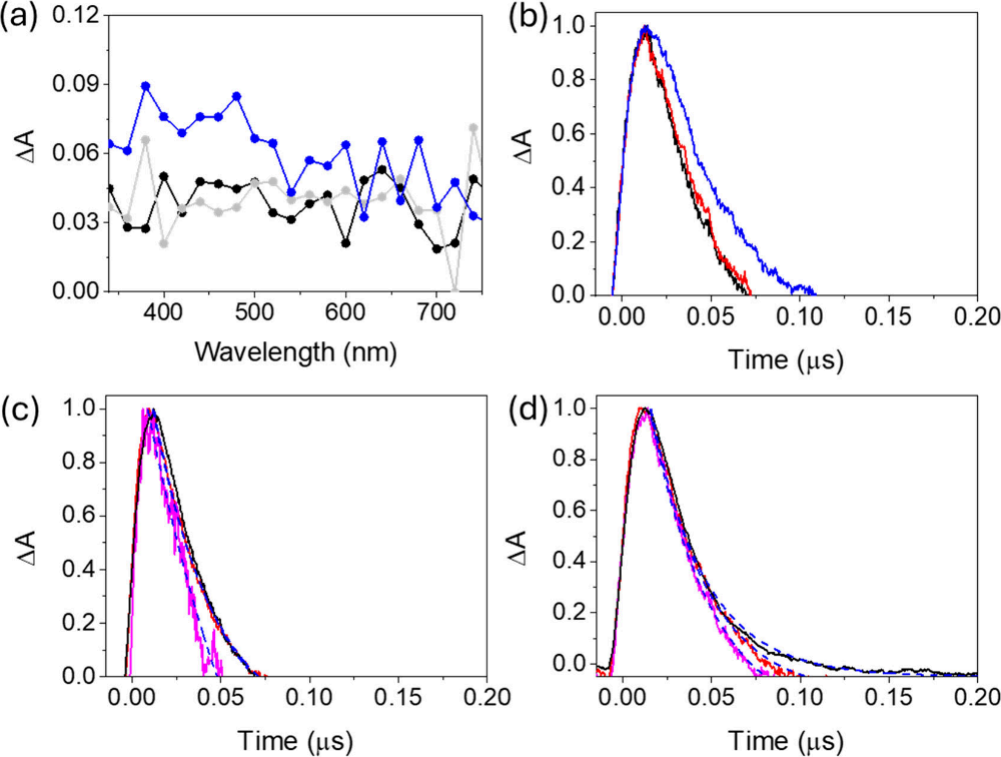
(a) LFP spectra 0.02 μs after the laser pulse for RuO_*x*_(2 wt %)@MIL-101­(Cr) acetonitrile suspensions under Ar (black) and O_2_ (gray) and in the presence of CH_3_OH (blue). (b) LFP decay traces for RuO_*x*_(2 wt %)@MIL-101­(Cr) at 450 nm in Ar (black) and O_2_ (red) and in the presence of CH_3_OH (blue). Decay traces for pristine MIL-101­(Cr) (black), RuO_*x*_(0.2 wt %)@MIL-101­(Cr) (red), and RuO_*x*_(2 wt %)@MIL-101­(Cr) (magenta) monitored at (c) 350 and (d) 450 nm under Ar conditions. All measurements were performed at λ_exc_ = 266 nm. The fitted curves in panels c and d are shown in dashed blue.

The kinetic profiles of the transient signals monitored at 350, 450, and 650 nm were also studied for RuO_*x*_(2 or 0.2 wt %)@MIL-101­(Cr) samples. Figure S47 shows that the presence of CH_3_OH considerably increases the average lifetime of the kinetics at 350 and 450 nm and to a much lesser extent at 650 nm. These findings agree with the above-mentioned discussion on the role of CH_3_OH as a hole scavenger, which partially quenches the transient species from ca. 650 to 750 nm associated with holes, with the simultaneous enhancement of transients from 340 to 650 nm. The higher the RuO_*x*_ loading supported on MIL-101­(Cr), the higher the increase of the average lifetime in the presence of CH_3_OH, again supporting the role of these NPs in the efficiency of photoinduced charge separation. Similar measurements carried out in the presence of O_2_ as an electron quencher revealed a relatively minor influence on the average lifetime. The results from the quenching experiments with CH_3_OH or O_2_, thus revealing that the photogenerated holes have a great influence on the recorded transient absorption spectra (TAS). To summarize, the TAS results are compatible with the photogeneration of electrons and holes.

Further analyses of the LFP kinetics at 350, 450, and 650 nm under an Ar atmosphere showed that the presence of increasing amounts of RuO_*x*_ NPs from 0.2 to 2 wt % within MIL-101­(Cr) induces faster relaxation of the transient species (Figures 10b,c and Figure S48). Interestingly, the fastest decay kinetics for RuO_*x*_(2 wt %)@MIL-101­(Cr) can be directly correlated with its highest photocatalytic activity. It should be noted that similar results were previously observed for related materials using MOF-based photocatalysts. For example, the order of photocatalytic activity observed for gas-phase CO_2_ methanation for RuO_*x*_ NPs supported UiO-66 solids followed the decreasing order UiO-66­(Zr/Ce/Ti) > UiO-66­(Zr/Ti) > UiO-66­(Ce) > UiO-66­(Zr) and increasing order of LFP lifetimes displaying values of ca. 0.04, 0.11, 0.12, and 5.37 μs, respectively.^31^ In other studies using defective UiO-66­(Zr)-NH_2_ solids, supported Pt NPs^54^ or Al-porphyrin MOFs modified with Pt in the form of NPs or single atoms,^55^ it was found that the increasing order of photocatalytic activity within the series of materials correlates with the faster relaxation dynamics determined by ultrafast TAS. In general, these studies^31,54,55^ showed that the photocatalysts with a higher activity exhibited the fastest relaxation kinetics and vice versa. It was proposed that long-lived species are related to deep trap charges with low redox ability to promote the reactions under study. The incorporation of RuO_*x*_ NPs^31^ and Pt^54,55^ single-atoms NPs accelerates the dynamics associated with the opening of additional charge carrier transfer pathways that favor the overall efficiency of the photoassisted catalytic processes.

Steady-state PL and TR-PL measurements were conducted to further study the excited-state properties and charge recombination of MIL-101­(Cr) and used RuO_*x*_(2 wt %)@MIL-101­(Cr). The wavelength-dependent PL spectra (Figures 11a,b) revealed a similar emission profile for both samples, with MIL-101­(Cr) exhibiting a prominent emission peak centered at 430 nm. The photoelectron–hole recombination rate in used RuO_*x*_(2 wt %)@MIL-101­(Cr) was also evaluated by PL spectroscopy whereby the emission peak at 430 nm (Figure 11c) was significantly reduced after incorporating RuO_*x*_ NPs into the MIL-101­(Cr) framework.^32^ This reduction indicates a lower recombination rate in used RuO_*x*_(2 wt %)@MIL-101­(Cr), which can be attributed to the formation of a surface junction between MIL-101­(Cr) and RuO_*x*_ NPs, which facilitates efficient separation of photogenerated electron–hole pairs. Thus, it significantly improves the photocatalytic performance of the material. A comparison of the TR-PL decay profiles of MIL-101­(Cr) and used RuO_*x*_(2 wt %)@MIL-101­(Cr) (Figure 11d and Table S4) revealed three-component decay kinetics in both samples. Notably, used RuO_*x*_(2 wt %)@MIL-101­(Cr) exhibited prolonged average lifetimes, reflecting enhanced charge carrier separation in the presence of RuO_*x*_ NPs.^32^

**Figure 11:**
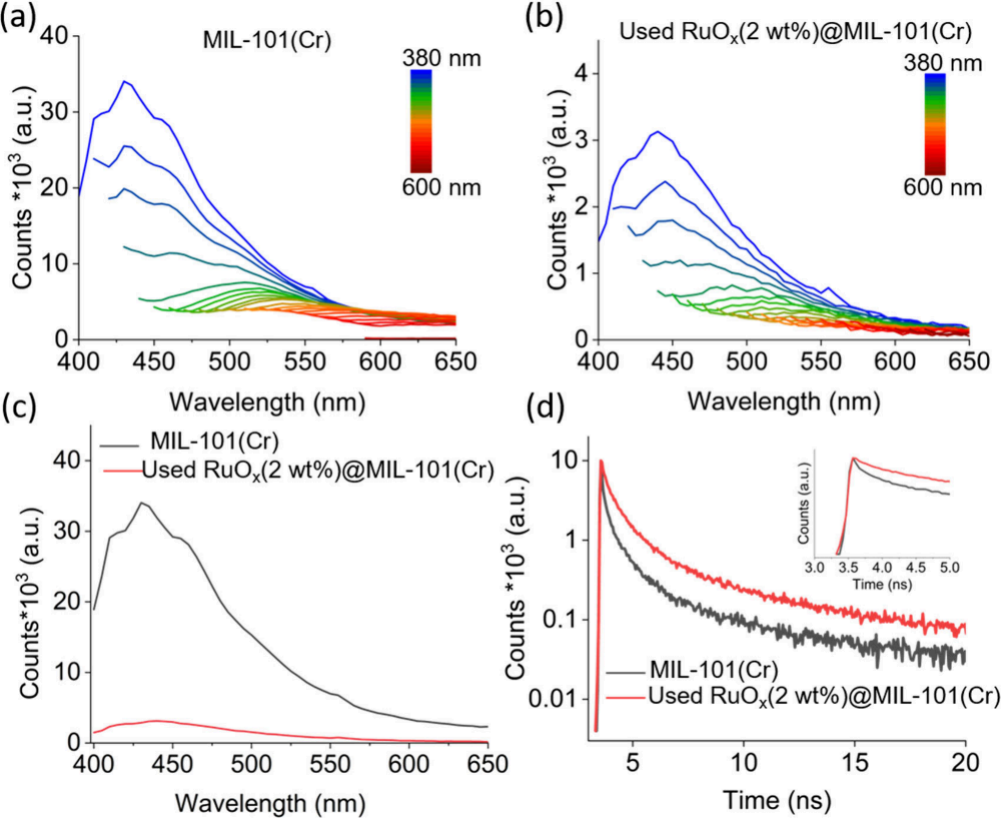
PL spectra of (a) MIL-101­(Cr) and (b) used RuO_*x*_(2 wt %)@MIL-101­(Cr) upon excitation from 380 to 600 nm. (c) PL spectra of MIL-101­(Cr) and used RuOx­(2 wt %)@MIL-101­(Cr). (d) Time-resolved decay of the emissions at 430 and 440 nm of MIL-101­(Cr) and used RuO_*x*_(2 wt %)@MIL-101­(Cr) pulsed by a 343 nm laser, respectively. Inset: zoomed-in view of the first 5 ns time scale.

Previous characterization by transient photocurrent, EIS, PL, and TAS showed that irradiating an MIL-101­(Cr) sample with and without RuO_*x*_ NPs generates electrons and holes, while the presence of increasing amounts of RuO_*x*_ NPs from 0.2 to 2 wt % favors the efficiency of the process, as shown by transient photocurrent and EIS measurements. Overall, these results agree with the operation of a photochemical pathway in which incident photons with sufficient energy can be transformed into charge carriers. Specifically, photogenerated holes and electrons are responsible for H_2_ oxidation and CO_2_ reduction, respectively. It is noteworthy to mention that transient photocurrent, EIS, PL, and LFP data to probe charge carrier behavior of a photocatalyst are meaningful for a photochemical reaction pathway only in the presence of an oxidizable substrate such as H_2_ and H_2_O among others. Otherwise, these techniques primarily reflect the inherent photophysics of the material.

In addition to the photochemical pathway, due to the presence of plasmonic RuO_*x*_ NPs within MIL-101­(Cr), the possibility of a photothermal mechanism during the photocatalytic process in which the photons are transformed into heat should be investigated.^51^

Attempts to measure possible temperature changes during photocatalyst irradiation under operating reaction conditions using an infrared thermometer revealed that the macroscopic temperature is maintained at 200 °C. It should be noted that the photothermally induced effect is concentrated on the surface of irradiated plasmonic RuO_*x*_ NPs (<3 nm) with temporal scales that can range from a femtosecond to a sub-nanosecond.^56^ To evaluate any potential photothermal effect, an *operando* FT-IR experiment was conducted on preactivated RuO_*x*_(2 wt %)@MIL-101­(Cr) (H_2_ flow at 200 °C for 1 h) under varying temperatures. As the temperature increased, the molecular vibrations of various species intensified, leading to potential shifts in the positions of FT-IR bands^57^ under irradiation with respect to dark condition, indicating an increase in local temperature.^58^ A calibration curve correlating the structural band position with the temperature was thus established (Figure 12a). The analysis revealed a linear shift of the band attributed to the asymmetric stretching of the C–O–C of the ligand molecules^59^ probably adjacent to an Ru site, from 1823.4 to 1819.5 cm^–1^, as the temperature increased from 30 to 250 °C under dark conditions (inset of Figure 12b). The photothermal behavior was then explored under irradiation at room temperature (RT), on which a significant band shift from 1823.4 to 1822.8 cm^–1^ was observed (Figure 12b). Based on these observations and the calibration curve, a localized temperature increase from RT to 102 ± 14 °C was determined. However, at higher temperatures (200 °C), the perturbation of the sample surface spectrum became more intense under irradiation, and we were not able to estimate the band shift with precision. It should be mentioned that no catalytic activity was found under irradiation at RT, suggesting that the photothermal effect is not the only factor driving the sample’s activity. The increased 5-fold activity in the presence of light, showing that it is driven by a combination of the photocatalytic, thermal and photothermal behavior.

**Figure 12:**
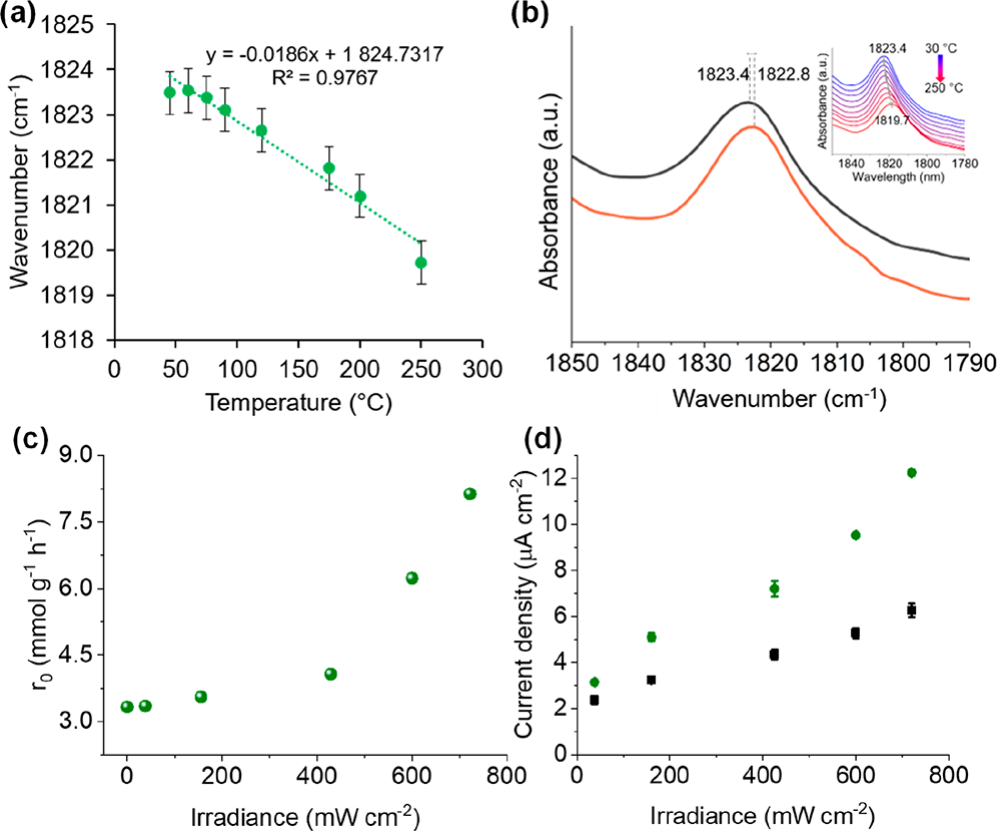
(a) Variation of the position of the 1823 cm^–1^ structural band of preactivated RuO_*x*_(2 wt %)@MIL-101­(Cr) under H_2_ at 200 °C for 1 h versus temperature under dark conditions. (b) Direct surface FT-IR spectra of preactivated RuO_*x*_(2 wt %)@MIL-101­(Cr) in the 1850–1780 cm^–1^ region in dark and under irradiation at 30 °C, respectively. Inset: FT-IR spectra of the same band at different temperatures in the dark. Spectra collected under continuous flow of Ar (10 cm^3^ min^–1^). (c) Influence of simulation sunlight irradiance intensity on photocatalytic CH_4_ formation. Reaction conditions: photocatalyst (15 mg), irradiation as indicated, and 200 °C. (d) Transient photocurrent intensity using RuO_*x*_(2 wt %)@MIL-101­(Cr).

As previous related studies proposed that the observation of an exponential relationship between either photocatalytic activity^51^ or transient photocurrent^60^ and light intensity can be ascribed to the operation of a photothermal pathway, the influence of light intensity on the photoassisted catalytic activity of RuO_*x*_(2 wt %)@MIL-101­(Cr) for CO_2_ hydrogenation to CH_4_ was studied. Figure 12c shows that there is a positive exponential-like relationship of light intensity with CH_4_ production. The quasi-linear relationship up to about 400 mW cm^–2^ can be interpreted by the operation of a photochemical pathway, which would agree with the previous characterization data. In contrast, the exponential relationship of activity versus light intensity can be ascribed to the operation of a photothermal reaction mechanism, as it has been described using similar systems.^51,61,62^ The presence of supported RuO_*x*_ NPs as cocatalysts that have a surface plasmon resonance band in the visible region has been described as being responsible for the operation of a photothermal mechanism in which photons are transformed into heat in these NPs. A observation between transient photocurrent and irradiance power was found when using RuO_*x*_(2 wt %)@MIL-101­(Cr) (Figure 12d). In contrast, previous experiments using pristine MIL-101­(Cr) solid showed a practically linear relationship with all of the irradiance power (Figure 12c). Overall, these experiments reinforce the idea that, on irradiation, the RuO_*x*_(2 wt %)@MIL-101­(Cr) photocatalyst operates under a photothermal mechanism at irradiances above ∼400 mW cm^–2^.

To sum up, RuO_*x*_@MIL-101­(Cr) can operate under a dual photochemical and photothermal reaction mechanism as illustrated in a simplified manner in Scheme 1.

**Scheme 1:**
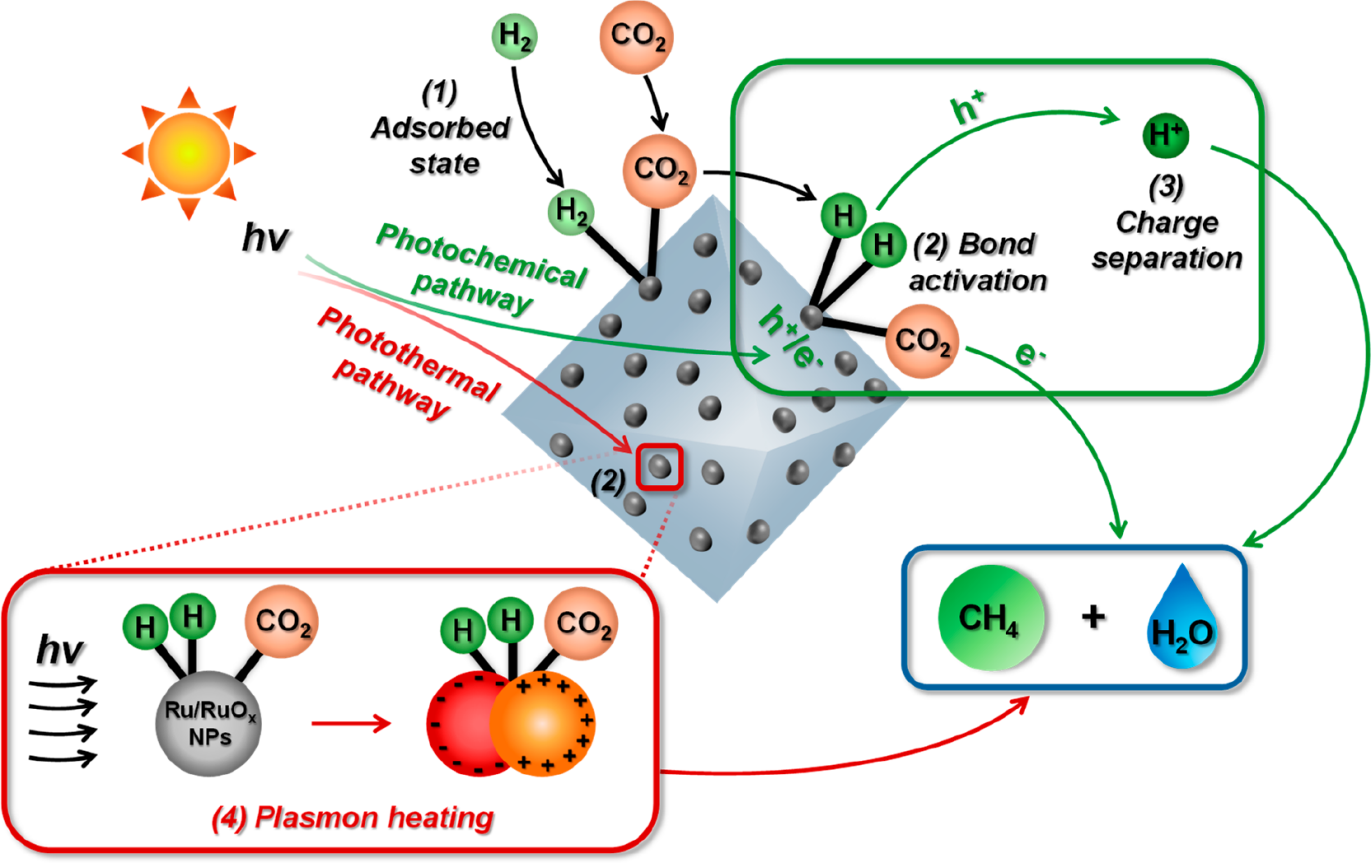
Simplified Illustration of Dual Photochemical and Photothermal Reaction Mechanism during Photocatalytic CO_2_ Hydrogenation to CH_4_ Using RuO_*x*_@MIL-101­(Cr)

### 3.4 Investigation of the Reaction Mechanism

The CO_2_ photomethanation reaction over RuO_*x*_(2 wt %)@MIL-101­(Cr) solid, previously pretreated at 200 °C in the presence of H_2_, was investigated using *operando* Raman and FT-IR spectroscopies to gain deeper insights into the reaction mechanism, with a particular focus on surface transformations occurring on the catalyst. These techniques enable real-time, simultaneous analysis of gas-phase products and photocatalysts’ surfaces under reaction conditions. Additionally, efforts were made to monitor potential modifications of the Ru centers during the reaction. However, due to the low Ru loading (2 wt %), no discernible Ru-related signals were observed. The Raman bands of RuO_*x*_(2 wt %)@MIL-101­(Cr) showed mainly the bands attributed to the BDC ligand from 600 to 1700 cm^–1^ similarly to other related MOFs^63^ as shown in Figure S49. Bands around 400 cm^–1^ are likely to originate from the chromium metal centers. These various bands did not significantly evolve during either activation or reaction, except for a slight shift probably due to the increase in temperature, showing the stability of the structure. However, during the reaction, new broad bands developed upon reaction. These bands at ca. 1330 and 1543 cm^–1^ could be due to the presence of organic adsorbed species on the surface of the catalyst.

To further investigate the nature and role of these surface species in the CO_2_ photoassisted methanation mechanism, *operando* FT-IR spectroscopy was employed.^64^ The surface IR spectra recorded during CO_2_ photomethanation on RuO_*x*_(2 wt %)@MIL-101­(Cr) between 30 and 200 °C are given in Figure 13a, with baseline correction applied using spectra collected after H_2_ activation at 30 °C. Direct spectra are found in Figure S50. It is important to note that these FT-IR spectra were obtained via online analysis, with representative steady-state spectra confirmed by the consistent presence of key peaks across at least 20 consecutive measurements to ensure reliability and minimize signal-to-noise artifacts. Gas-phase product analysis under similar conditions is shown in Figure 13b. *Operando* FT-IR analysis of the catalyst surface revealed critical intermediates. Notably, CO_2_ adsorption was found in the region of 2400 to 2250 cm^–1^, characterized by a chemisorbed CO_2_ band centered at 2236 cm^–1^ alongside contributions from gas-phase CO_2_. An exponential decrease in chemisorbed CO_2_ concentration was found with increasing temperature, which disappeared at approximately 30 °C. It is noteworthy that MIL-101­(Cr) showed significantly enhanced CO_2_ adsorption (Figure S51) compared to RuO_*x*_(2 wt %)@MIL-101­(Cr) (Figure 13a). However, despite this high adsorption capacity, the CO_2_ could not be converted to CH_4_ as a final product since the MIL-101­(Cr) sample recorded a very low activity with only traces of CO production.

**Figure 13:**
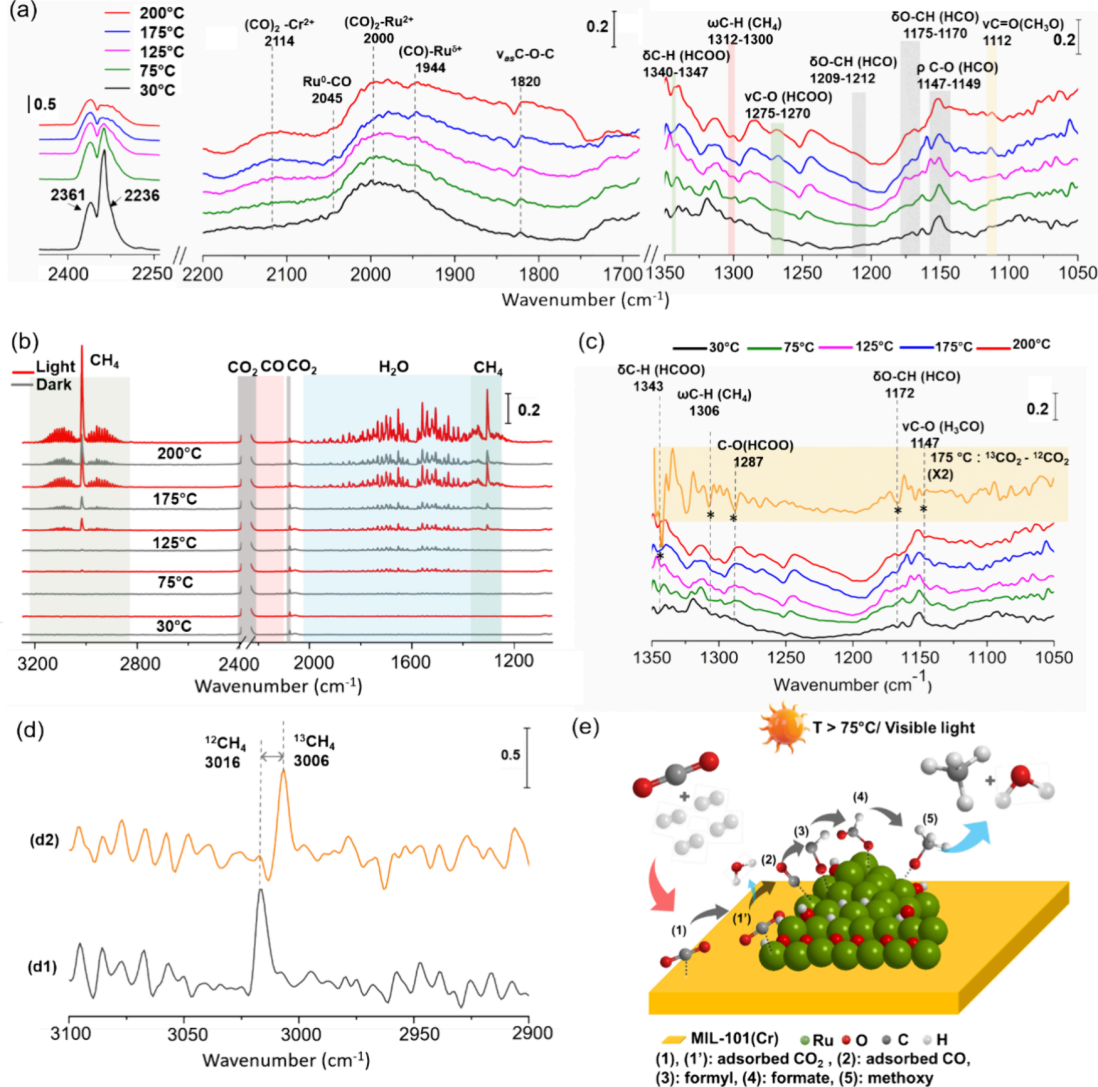
(a)*Operando* FT-IR spectra of RuO_*x*_(2 wt %)@MIL-101­(Cr) during photocatalytic CO_2_ methanation versus temperature: in the 2400–2250, 2200–1800, and the 1200–1000 cm^–1^ vibrational regions. (b) Corresponding FT-IR spectra of the reaction gas phase in dark and after irradiation at steady state. (c) FT-IR spectra of the catalyst with ^12^CO_2_ and the corresponding subtracted spectra: ^13^CO_2_ – ^12^CO_2_ (intensity was multiplied by two for clarification were noted). The * corresponds to the bands formed in the ^12^CO_2_ reaction resulting in a negative peak in the (^13^CO_2_ - ^12^CO_2_) spectrum. (d) FT-IR gas phase spectra of gaseous CH_4_ produced with (d1) ^12^CO_2_ and (d2) ^13^CO_2_. The arrow corresponds to the shift of the FT-IR bands due to the isotopic exchange from ^12^CO_2_ to ^13^CO_2_. The assignments of the different IR bands are summarized in Tables S5 and S6. (e) Proposed mechanism of the photoassisted CO_2_ methanation over RuO_*x*_(2 wt %)@MIL-101­(Cr) based on the assignment of the characteristic FT-IR bands of the different species.

Various vibrational bands were seen in the CO region, although assigning specific band positions to the different adsorption sites remains challenging, as shown by the diverse interpretations reported in the literature.^65,66^ This variability is probably attributed to several factors that influence the precise band positions, including the coverage levels of the CO and the oxidation state of the adsorption sites. For instance, a band at 2114 cm^–1^, attributed to the bridging carbonyls adsorbed on Cr^2+^, emerged with increasing temperature in both MIL-101­(Cr) (Figure S52) and RuO_*x*_(2 wt %)@MIL-101­(Cr) (Figure 13a). This band is primarily associated with CO production, which is the dominant product in the pristine MIL-101­(Cr) sample. However, while no significant evolution of surface bands was observed for MIL-101­(Cr), RuO_*x*_(2 wt %)@MIL-101­(Cr) revealed the presence of key intermediates critical for the methanation process. For example, a distinct band was found at 2000 cm^–1^, attributed to the asymmetric stretching vibrations of Ru^2+^(CO)_2_ species.^67^ Moreover, a peak at a lower wavenumber, i.e., at 1944 cm^–1^, appeared at 75 °C, coinciding with the onset of CH_4_ production (Figures 13a,b). This peak intensified with increasing temperature and is assigned to the monocarbonyls adsorbed on less oxidized Ru^δ+^ sites.^67^ A less intense peak at 2045 cm^–1^ was also detected, possibly corresponding to CO linearly bound to metallic Ru^0^. A comprehensive summary of these spectral assignments is provided in Table S5. These findings suggest that CO is formed at lower temperatures and serves as a potential intermediate, subsequently converting to CH_4_ at high temperatures. Significant changes were seen in the 1300 to 1000 cm^–1^ region as illustrated in Figure 13a. The band at 1172 cm^–1^, attributed to formyl species, intensified with increasing temperature.^68^ At higher temperatures, bands at 1343 and 1286 cm^–1^ grew more prominently, corresponding to the C–H bending and C–O stretching vibrations of the monodentate formate species, respectively.^69^ Methoxy species were identified by a band at 1147 cm^–1^, associated with their characteristic rocking vibration.^70^ These findings highlight the critical role of the CO, formyl, formate, and methoxy species as intermediates in the methanation reaction.

To confirm the identity of intermediates, steady-state isotopic transient kinetic analysis (SSITKA) was conducted at 175 °C by using *operando* FT-IR spectroscopy. The isotopic transient experiment involved substituting ^12^CO_2_ with its isotope ^13^CO_2_ under steady-state conditions under identical reaction parameters. This isotopic exchange causes a measurable shift in the FT-IR bands, corresponding to both the final products and the intermediate species adsorbed on the catalyst surface. Importantly, this approach ensured that the observed spectral shifts arose exclusively from the isotopic exchange between ^13^CO_2_ and ^12^CO_2_, eliminating the influence of temperature variations on the results. Given the high cost of pure ^13^CO_2_, the experiment was conducted under diluted conditions, using 1% ^12^CO_2_ in Ar flow initially, followed by a switch to 1% ^13^CO_2_ in Ar. Remarkably, ^13^CH_4_ was produced from ^13^CO_2_ in quantities comparable to those obtained with ^12^CO_2_ under the same diluted conditions (Figure 13d). This indicates that the produced CH_4_ comes solely from CO_2_ reduction. This also induced shifts of 3–4 cm^–1^ in IR bands corresponding to formyl, formate, and methoxy (Figure 13c). It is important to highlight that no discernible shift was observed in the CO region during the isotopic transient experiment. The broad nature of the CO bands, combined with the diluted experimental conditions, hindered the detection of these bands under the specified operating conditions of our equipment. These results are summarized in Table S6, and to confirm that CO could be considered as the first intermediate of the reaction, two reactions were conducted separately: one under CO flow only and the other under a CO and H_2_ flow (Figure S53). The results showed that RuO_*x*_(2 wt %)@MIL-101­(Cr) catalyzed CO oxidation on its surface, with significant CO_2_ production under CO or CO/H_2_ flow conditions. This behavior could be attributed to the water–gas shift reaction facilitated by surface-bound H_2_O:

1 $$\mathrm{CO}\left(g\right)+H_{2}O⇌{\mathrm{CO}}_{2}\left(g\right)+H_{2}\left(g\right)$$

CH_4_ production was also detected, probably due to the following reactions:

2 $$\mathrm{CO}\left(g\right)+3H_{2}\left(g\right)⇌{\mathrm{CH}}_{4}\left(g\right)+H_{2}O$$

3 $$2\mathrm{CO}\left(g\right)+2H_{2}\left(g\right)⇌{\mathrm{CH}}_{4}\left(g\right)+{\mathrm{CO}}_{2}\left(g\right)$$

These findings highlight the possible role of CO as an intermediate in the CO_2_ methanation reaction.

Based on the above-mentioned spectral investigations, a proposed photoassisted overall mechanism for the reaction unfolds, as shown in Figure 13e. Initially, CO_2_ is adsorbed on the surface of MIL-101­(Cr) and Ru species in the presence of H_2_. CO is then generated as the primary intermediate, which exhibits strong surface adsorption, as evidenced from the very low CO production in the gas phase and surface analysis, further confirmed by FT-IR (Figure 13b). As the temperature increases, CO is transformed to formyl, followed by the formation of formate as a third intermediate through the interaction with surface oxygen. This process eventually leads to the formation of methoxy as the final intermediate species before CH_4_ and H_2_O are generated as final products through further reduction. This study emphasizes the dual role of RuO_*x*_ and reduced Ru in the production of CH_4_ from CO_2_ and H_2._

## 4 Conclusions

This work describes the development of highly active and selective solar-driven photocatalysts for gaseous CO_2_ methanation using Cr-based MIL-101 materials supported by RuO_*x*_ NPs. The activity of RuO_*x*_(1 wt %)@MIL-101­(Cr) is between 3 and 50 times higher than related MOF-based photocatalysts under similar reaction conditions. An optimized RuO_*x*_(2 wt %)@MIL-101­(Cr) photocatalyst afforded 98.1% CO_2_ conversion with 98.8% CH_4_ selectivity with a production rate of 7.85 mmol g^–1^ h^–1^ with 720 mW cm^–2^ at 200 °C. This photocatalyst achieved AQY of 9.2% with 600 nm at 200 °C after subtracting the thermal activity contribution. To the best of our knowledge, this AQY value ranks this material as the most active heterogeneous photocatalysts under visible-light irradiation described for this purpose so far. RuO_*x*_(2 wt %)@MIL-101­(Cr) was found to be a stable and reusable photocatalyst after five consecutive uses with an accumulated operation time of 110 h. In terms of reaction pathway for CO_2_ photomethanation, a dual photochemical and photothermal reaction pathway is evidenced based on the photocatalytic results together with transient photocurrent, EIS, FT-IR, and PL and LFP spectroscopies combined with additional photocatalytic experiments. *Operando* FT-IR spectroscopy revealed some information on the basic steps for selective photocatalytic CO_2_ hydrogenation to CH_4_ via methoxy intermediates.

## Supplementary Material



## Notes

Complete characterizations using SEM, TEM, XPS, and TGA for the pristine MOFs, RuOx@MIL-101­(Cr), and RuOx@MIL-101­(Fe), *operando* FT-IR, Raman characterizations, comparison of the photocatalytic performance along with AQY values, GC-MS, and laser flash photolysis experiments (PDF)
