# Supplementary material for: Photo-thermal Catalytic CO2 Methanation by RuO x @MIL-101(Cr) with 9.2% Apparent Quantum Yield under Visible Light Irradiation
Source: ACS Appl Mater Interfaces. 2025 Aug 25;17(35):49485–99. doi: 10.1021/acsami.5c10215 (PMC12412102; doi:10.1021/acsami.5c10215)
Supplement: Supplementary file 1 [file am5c10215_si_001.pdf]

# Supporting Information

# Photo-thermal Catalytic $CO_2$ Methanation by RuOx@MIL-101(Cr) with 9.2 % Apparent Quantum Yield under Visible Light Irradiation

Juan José Ramírez-Hernández,[1] Vitor Fernandes de Almeida, [1] Zahraa Abou-Khalil,[2] Belén Ferrer,[1] Francesc X. Llabrés i Xamena,[4] Marco Daturi,[2] Guillaume Clet,[2] Ignacio Vayá,[1,4] Herme G. Baldoví,[1] Amarajothi Dhakshinamoorthy,[1,3,*] Mohamad El-Roz,[2,*] Sergio Navalón[1,*]

[1] Departamento de Química, Universitat Politècnica de València, Camino de Vera s/n, Valencia 46022, Spain.

[2] Université de Caen Normandie, ENSICAEN, CNRS, LCS, 14000 Caen, France.

[3] School of Chemistry, Madurai Kamaraj University, Madurai, 625021, Tamil Nadu, India.

[4] Instituto de Tecnología Química (CSIC-UPV), Universitat Politècnica de València, Consejo Superior de Investigaciones Científicas, Av. de los Naranjos s/n, 46022 Valencia, Spain.

E-mail: dhaam@qim.upv.es (AD); mohamad.elroz@ensicaen.fr (ME); sernaol@doctor.upv.es (SN)

## S1. Reagents and reactants

The compounds used for the preparation of MOFs as well as solvents and reagents in this work were obtained from MERCK with analytical or HPLC grade. Terephthalic acid (ref. 185361), chromium(III) nitrate nonahydrate ($Cr(NO_3)_3·9H_2O$, ref. 239259), hydrofluoric acid (HF, ref. 695068), iron(III) chloride hexahydrate ($FeCl_3·6H_2O$, ref. F2877), dimethylformamide (DMF, ref. 319937), acetic acid (ref. 695092), methanol (ref. 34860), $Cr_2O_3$ (ref. 203068), γ-$Fe_2O_3$ (ref. 544884), potassium perrhuthenate ($KRuO_4$, ref. 334537), terpineol (ref. 77663), tetrabutylammonium hexafluorophosphate (ref. 86879), acetone (ref. 270725) and acetonitrile (ref. 34851).

## S2. Photocatalyst preparation

### Synthesis of MIL-101(Cr)

The synthesis of MIL-101(Cr) was carried out following modified procedures.[1] Firstly, terephthalic acid (665 mg, 4 mmol) and $Cr(NO_3)_3$ (1600 mg, 4 mmol) were added into a round-bottom flask with 19.2 mL (1.06 mol) of deionized $H_2O$ and sonicated in an ultrasound bath for 30 minutes. Then, the solution and hydrofluoric acid (0.8 mL, 4 mmol) were incorporated inside a 30 mL Teflon-lined stainless-steel autoclave and the hydrothermal reaction occurs at 220 ºC for 8 h. After this time, the autoclave was cool down to room temperature and the dark green solution with unreacted reagents was centrifuged. Then, the solution was washed several times with DMF at 120 ºC overnight and hot filtered at each time to remove unreacted organic ligand and, successively, washed with MeOH at 60 ºC several times. Finally, the greenish powder was dried in an oven at 100 ºC and activated using vacuum at 150 ºC overnight.

### Synthesis of MIL-101(Fe)

The synthesis of MIL-101(Fe) was made following steps reported in the bibliography.[2] Firstly, terephthalic acid (658 mg, 3.96 mmol) and $FeCl_3·6H_2O$ (1.07 g, 3.96 mmol) were dissolved in DMF (90 mL) using a round-bottom flask (250 mL). Subsequently, acetic acid (3.6 mL) was added and the system heated at 110 ºC for 24 h. After this time, the system was cooled down to room temperature and the obtained light orange solution with unreacted reagents was centrifuged. Then, the solution was washed several times with DMF at 120 ºC overnight and hot filtered at each time to remove unreacted organic ligand and, successively, washed with MeOH at 60 ºC several times.

Finally, the orangish powder was dried in an oven at 100 ºC and activated using vacuum at 150 ºC overnight.

**Deposition of $RuO_x$ nanoparticles**

$RuO_x$ nanoparticles (NPs) were deposited in the as-prepared MOF or commercial metal oxide ($Cr_2O_3$ or $\gamma$-$Fe_2O_3$) solids using photodeposition method.[3] The solid (50 mg) was charged into a quartz tube containing a mixture of Milli-Q water (8 mL) and methanol (13 mL). Subsequently, the respective amount of potassium perruthenate (1wt% Ru) previously dissolved in water (1 mL) was added to the MOF dispersion. Then, the mixture was purged with Ar for 30 min and the system was immediately irradiated with UV-Vis lamp for 4 h. Finally, the resulting solid was filtered, washed several times with Milli-Q water and placed in an oven at 100 ºC overnight and then, left under vacuum at 150 ºC, 24 h to remove the solvents.

**S.3. Characterization methods**

Powder X-ray diffraction (XRD) measurements were collected from a Philips XPert diffractometer equipped with a graphite monochromator (40 kV and 45 mA) employing Ni filtered $CuK_\alpha$ radiation (0.15418 nm). UV-Vis diffuse reflectance spectroscopy (DRS) measurements were performed on a Varian spectrometer model Cary 5000. X-ray photoelectron spectroscopy (XPS) were acquired on a SPECS spectrometer equipped with an MCD-9 detector using a monochromatic Al X-ray source ($K_\alpha$= 1486.6 eV). The C 1s peak at 284.4 eV was employed as reference binding energy. CASA software has been employed for spectra deconvolution. The highest occupied crystal orbital (HOCO) energy maximum value of the photocatalyst respect to the Fermi level ($E^f_\upsilon$) was determined by XPS. In addition, the HOCO value versus NHE ($E_\upsilon^{NHE}$) was determined from the equation: $E_\upsilon^{NHE} = E_\upsilon^f + \varphi_{sp} + E^0_{SHE}$, where $\varphi_{sp}$ is the work function of the XPS instrument used for the measurements (4.244 eV) and $E^0_{SHE}$ is the energy of the SHE with respect to vacuum level of the electron (-4.44 eV). The lowest unoccupied crystal orbital (LUCO) band energy minimum of the photocatalyst was estimated by the difference between the $E^\upsilon_{NHE}$ value and the optical band gap.

In situ XPS hydrogenations of $RuO_x$(2 wt%)@MIL-101(Cr) were carried out on a SPECS GmbH photoelectron spectroscopy instrument with UHV system and PHOIBOS 150 9MCD energy analyzer.

Scanning electron microscopy (SEM) images were carried out using a scanning electron microscope (SEM, Zeiss instrument, AURIGA Compact) equipped with an energy-dispersive X-ray (EDX) detector. Transmission electron microscopy images were collected on a JEOL JEM2100F instrument (200 kW). Isothermal $N_2$ adsorption measurements were performed at 77 K using a Micromeritics station (ASAP 2010). Thermogravimetric analyses were carried out on a TGA/SDTA851e METTLER TOLEDO station.

Photoelectrochemical analysis including electrochemical impedance spectroscopy (EIS) and photocurrent measurements were performed using a Gamry Instruments potentiostat (model Interface 5000E). A standard three-electrode configuration was employed in a home-made quartz electrochemical cell with a platinum wire as counter electrode and a saturated Ag/AgCl electrode as the reference. The working electrode was prepared as given below. Firstly, a paste was prepared from each material by mixing 20 mg of a photocatalyst with 0.2 mL of terpineol and 0.5 mL of acetone. Secondly, the mixture was left stirring until complete dispersion. Afterwards, the mixture was left under stirring and incubation at 90 ºC overnight. After letting cool down, 25 µL of each sample was spread onto an area of 1.0 x 1.0 $cm^2$ using doctor blade technique though a conductive carbon Toray paper with dimensions of 2.0 x 1.0 $cm^2$. Finally, the electrode was thermally treated at 150 °C for 1 h. The photocurrent generated by the electrodes was measured by chopped linear sweep voltammetry (LSV) with an electrolyte of tetrabutylammonium hexafluorophosphate (0.1 M; $TBAPF_6$) acetonitrile solution. In some cases, the applied potential was +0.9 V and for some LSV measurements, MeOH (0.3 mL) or $H_2$ ($CH_3CN$ purged solution with $H_2$ at 10 mL/min for 5 min) was added as a hole scavenger.

EIS measurements were carried out with frequencies ranging from 0.1 Hz to 100 kHz with (+0.2 V) or without polarization of the working electrode. Prior to these measurements, the electrolyte solutions were purged with argon for 10 min. UV-Vis irradiation of the working electrodes was carried out with a spot light Hamamatzu Xe lamp (Lightningcure LC8 model, 800–200 nm, 1000 W $m^{-2}$, fiber optic light guide with a spot size of 0.5 cm). A Randles equivalent circuit was employed to fit each Nyquist spectrum and consists of a solution resistance, a double layer capacitor and a charge transfer resistance (Rct).[4] In some experiments, $H_2$ purged $CH_3CN$ solutions ($H_2$ at 10 mL/min for 5 min) were performed.

Laser flash photolysis (LFP) measurements were performed using a pulsed Nd:YAG L52137 V LOTIS TII at $\lambda_{exc}$ = 266 nm. The single pulses were *ca*. 10 ns duration, and the energy was ~10 mJ/pulse. The LFP system consisted of the pulsed laser, a 77250 Oriel monochromator and an oscilloscope DP04054 Tektronix. The output signal from the oscilloscope was transferred to a personal computer. Absorbances of all solutions were adjusted at ~0.30 at the excitation wavelength in acetonitrile (HPLC grade). Measurements were done using 10 × 10 $mm^2$ quartz cuvettes at room temperature in different atmospheres: argon or oxygen (25 min bubbling). Control experiments indicated that the degree of decomposition of the samples after photolysis was lower than 2%. The LFP decay traces were fitted using a monoexponential function following the Levenberg-Marquardt iteration algorithm (equation 1):

$$\boldsymbol{F(t,\lambda) = \sum_{i=1}^{n} a_i(\lambda) e^{(-t/\tau_i)} + y_0}$$

Solid state emission spectra were recorded using an FS1000 spectrometer (Edinburgh, UK) equipped with an N-J03 as the sample holder with a front-face geometry, which also used a Xenon lamp to excite the sample, which were placed between the sample holder and the emission monochromator. The time-resolved decay was collected using the same spectrometer equipped with a femtosecond laser as excitation source (Satasuma Display AMPLITUDE) and a high-speed hybrid photodetector.

## S.4. Photocatalytic tests

### S4.1. Photocatalytic experiments under batch conditions

Photocatalytic experiments under batch reaction conditions were performed using a quartz reactor (50 mL) placed in an aluminum heating block equipped with a thermocouple. The photocatalyst (15 mg) was placed at the bottom of the reactor. Then, the reactor was pressurized with $H_2$ for 15 min and followed by $CO_2$ to attain a $H_2$:$CO_2$ ratio 4:1 and 1.5 bar pressure. Irradiations were performed using a Hg-Xe lamp (150 W, Hamamatsu ref. L8253; Hamamatsu spotlight source L9566-04 and light guide A10014-50–0110) with or without an AM 1.5G type filter (Lasing ref. 81094) to obtain simulated sunlight irradiation. The influence of radiation intensity on the photocatalytic activity was carried out by using transmittance filters (Newport, ref. FSQ-OD30, FSQ-OD15 or FSQ-

QD05). The irradiance intensity was measured using a commercially available thermal photodiode 919P-003-10 Therm, 3 W, 10 mm with 843-R-USB handheld power meter as hardware. The course of the reaction was monitored by analysing gas phase reaction aliquots in an Agilent 490 MicroGC equipped with a thermal conductivity detector. A MolSieve 5Å column was used to analyse $H_2$, $O_2$, $N_2$, and CO while a Pore Plot Q column to analyse the $CO_2$, $CH_4$, and short-chain hydrocarbons (ethane, ethylene, propane, propylene, and butane). Quantification was done by calibration plots with commercially available gas mixtures.

### S.4.2. Operando FT-IR photocatalytic tests

Photocatalytic activity was evaluated using an advanced operando Fourier-transform infrared (FT-IR) setup, enabling simultaneous real-time monitoring of both the catalyst surface and gas-phase reaction products. The experiments were conducted in a specialized "sandwich" IR cell reactor under the following conditions: a 4:1 molar ratio of $H_2$ to $CO_2$ with a total flow rate of 10 $cm^3$ $min^{-1}$, visible light irradiation (Xe lamp, LC8 Hamamatsu, $\lambda > 390$ nm, irradiance = 70 mW $cm^{-2}$), and 20 mg of the photocatalyst. Prior to testing, the photocatalyst was preactivated under $H_2$ at 200 °C, with a heating rate of 5 °C $min^{-1}$. Reactions were then carried out at varying temperatures both in the dark and under illumination. More technical details can be found elsewhere.[5-7] he surface of the catalyst and the composition of the reactor's output gas were concurrently analyzed using a FT-IR spectrometer (Thermo Nicolet NEXUS 670 FT-IR) equipped with a mercury cadmium telluride (MCT) detector (spectral resolution: 4 $cm^{-1}$, 64 scans accumulated). Additionally, a quadrupole mass spectrometer (Pfeiffer Omnistar GSD 301) was employed to continuously monitor gas composition during the reaction. The identity and quantity of the produced gases were further validated using an online gas chromatograph (Compact-GC) equipped with an Rtx-1-5u (30 m × 0.32 mm) capillary column and a flame ionization detector (FID).

It should be noted that while the FT-IR instrumental resolution used was 2 $cm^{-1}$, this value refers to the minimum separation between two spectral features that the instrument can distinguish as distinct. Although the instrumental resolution is 2 $cm^{-1}$, the center position of a peak can be determined with much finer precision, often below 1 $cm^{-1}$, depending on the signal-to-noise ratio and spectral stability. In our analysis, the 0.6 $cm^{-1}$ peak shift was extracted from high signal-to-noise ratio. The peak position

reproducibility was evaluated through repeated measurements, which yielded standard deviations within ±0.1 cm$^{-1}$, confirming that sub-wavenumber shifts can be reliably detected under our experimental conditions.

### S.4.3. Operando Raman experiments

Raman spectra were acquired using a Horiba Labram HR Evolution Raman microscope with a 532 nm laser excitation, while the catalyst (~30 mg) was placed in a Linkam CCR-1000 heating cell. Prior to the reaction, the sample was pretreated under a gas flow of 2 mL min$^{-1}$ Ar and 8 mL min$^{-1}$ $H_2$. For the reaction conditions, Ar was replaced by $CO_2$, maintaining a 2:8 mL min$^{-1}$ $CO_2$:$H_2$ gas mixture, and the system was exposed to visible light irradiation (Xe lamp, LC8 Hamamatsu, λ > 390 nm, irradiance = 71 mW cm$^{-2}$).

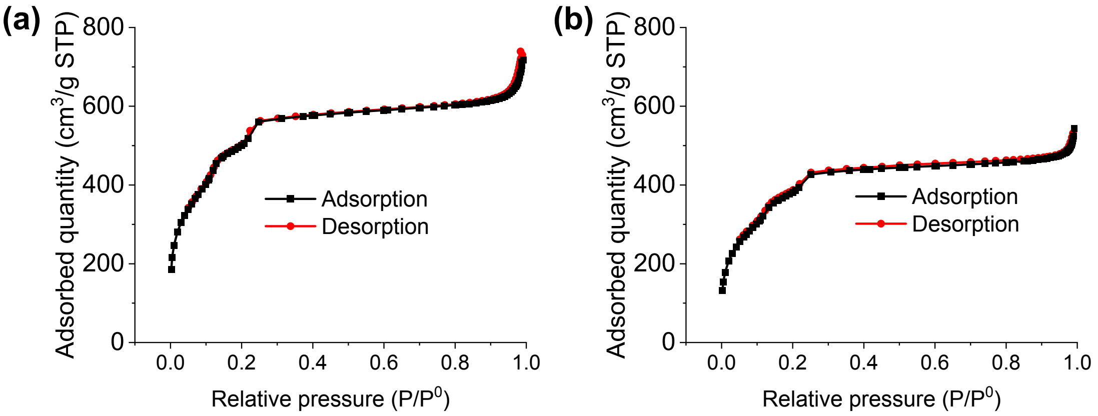


Figure S1. Isothermal $N_2$ adsorption measurement for (a) MIL-101(Cr) and (b) MIL-101(Fe).

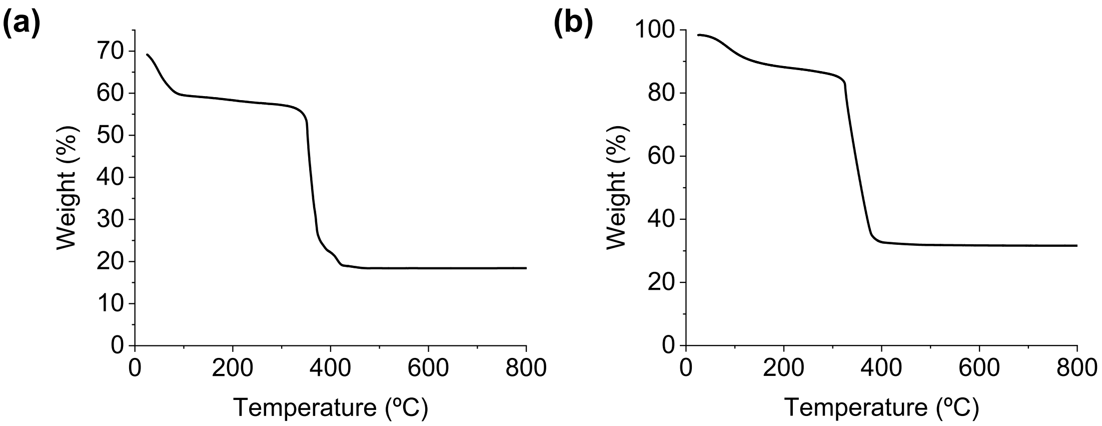

Figure S2. TGA profile of (a) MIL-101(Cr) and (b) MIL-101(Fe).

Table S1. Theoretical and experimental metal content in MIL-101(Cr) and MIL-101(Fe).

| Material | Theoretical metal content (%) | Experimental metal content from thermogravimetric analysis (%) |
|---|---|---|
| MIL-101(Cr) | 21.70 | 22.21 |
| MIL-101(Fe) | 23.03 | 25.57 |

| Table S2. Photocatalytic $CO_2$ hydrogenation to $CH_4$ using MOF photocatalysts reported in literature. | | | | | | |
|---|---|---|---|---|---|---|
| Entry | Photocatalyst | Co-catalyst | Irradiation conditions | Reaction conditions | $CH_4$ production (mmol $g^{-1}$ $h^{-1}$) | Ref. |
| 1 | MIL-101(Cr) | $RuO_x$ (1 wt%) | Simulated solar light irradiation (150 W Hg-Xe lamp equipped with an AM 1.5 filter; 420 mW $cm^{-2}$) | 1.3 bar $H_2$, 0.2 bar $CO_2$, 200 ºC | 1.99 | This work |
| 2 | UiO-66(Zr/Ti)-$NO_2$ | $RuO_x$ (1 wt%) | Simulated solar light irradiation (150 W Hg-Xe lamp equipped with an AM 1.5 filter; 390 mW $cm^{-2}$) | 1.3 bar $H_2$, 0.2 bar $CO_2$, 200ºC | 0.68 | [8] |
| 3 | UiO-66(Zr/Ce/Ti) | $RuO_x$ (1 wt%) | Simulated solar light irradiation (150 W Hg-Xe lamp equipped with an AM 1.5 filter; 350 mW/$cm^2$) | 1.3 bar $H_2$, 0.2 bar $CO_2$, 200ºC | 0.05 | [9] |
| 4 | UiO-66(Zr/Ce/Ti) | $RuO_x$ (1 wt%) | Simulated solar light irradiation (150 W Hg-Xe lamp equipped with an AM 1.5 filter; 450 mW/$cm^2$) | 1.3 bar $H_2$, 0.2 bar $CO_2$, 200ºC | 0.105 | [9] |
| 5 | UiO-66(Zr/Ce/Ti) | $RuO_x$ (1 wt%) | Simulated solar light irradiation (150 W Hg-Xe lamp equipped with an AM 1.5 filter; 500 mW/$cm^2$) | 1.3 bar $H_2$, 0.2 bar $CO_2$, 200ºC | 0.14 | [9] |

| | | | | | | |
|---|---|---|---|---|---|---|
| 6 | UiO-66(Zr/Ti) | $RuO_x$ (1 wt%) | Simulated solar light irradiation (150 W Hg-Xe lamp equipped with an AM 1.5 filter; 500 $mW/cm^2$) | 1.3 bar $H_2$, 0.2 bar $CO_2$, 200ºC | 0.088 | 9 |
| 7 | UiO-66(Zr/Ce) | $RuO_x$ (1 wt%) | Simulated solar light irradiation (150 W Hg-Xe lamp equipped with an AM 1.5 filter; 500 $mW/cm^2$) | 1.3 bar $H_2$, 0.2 bar $CO_2$, 200ºC | 0.063 | 9 |
| 8 | UiO-66(Ce) | $RuO_x$ (1 wt%) | Simulated solar light irradiation (150 W Hg-Xe lamp equipped with an AM 1.5 filter; 500 $mW/cm^2$) | 1.3 bar $H_2$, 0.2 bar $CO_2$, 200ºC | 0.052 | 9 |
| 9 | UiO-66(Zr) | $RuO_x$ (1 wt%) | Simulated solar light irradiation (150 W Hg-Xe lamp equipped with an AM 1.5 filter; 500 $mW/cm^2$) | 1.3 bar $H_2$, 0.2 bar $CO_2$, 200ºC | 0.049 | 9 |
| 10 | MIP-208(Ti) | $RuO_x$ (0.76 wt%) | Simulated solar light irradiation (150 W Hg-Xe lamp equipped with an AM filter; 100 $mW/cm^2$) | 1.05 bar $H_2$, 0.25 bar $CO_2$, 200ºC | 0.036 | 10 |
| 11 | MOF-Zn(1) | $Cu_2O$ (1 wt%) | UV-Vis light irradiation (300 W Xe lamp, 224 $mW/cm^2$) | 1.05 bar $H_2$, 0.25 bar $CO_2$, 215ºC | 0.01 | 11 |
| 12 | MIL-101(Cr) | $RuO_x$ (2 wt%) | Simulated solar light irradiation (150 W Hg-Xe lamp equipped with an AM 1.5 filter; 420 mW $cm^{-2}$) | 1.3 bar $H_2$, 0.2 bar $CO_2$, 200 ºC | 4.07 | This work |

| | | | | | | |
|---|---|---|---|---|---|---|
| 13 | MIL-101(Cr) | $RuO_x$ (2 wt%) | Simulated solar light irradiation (150 W Hg-Xe lamp equipped with an AM 1.5 filter; 720 mW $cm^{-2}$) | 1.3 bar $H_2$, 0.2 bar $CO_2$, 200 ºC | 7.85 | This wor k |
| 14 | MIL-125(Ti)-$NH_2$ | $RuO_x$ (10 wt%) | Simulated solar light irradiation (150 W Hg-Xe lamp equipped with an AM filter; 1120 $mW/cm^2$) | 1.05 bar $H_2$, 0.25 bar $CO_2$, 200ºC | 0.98 | 12 |

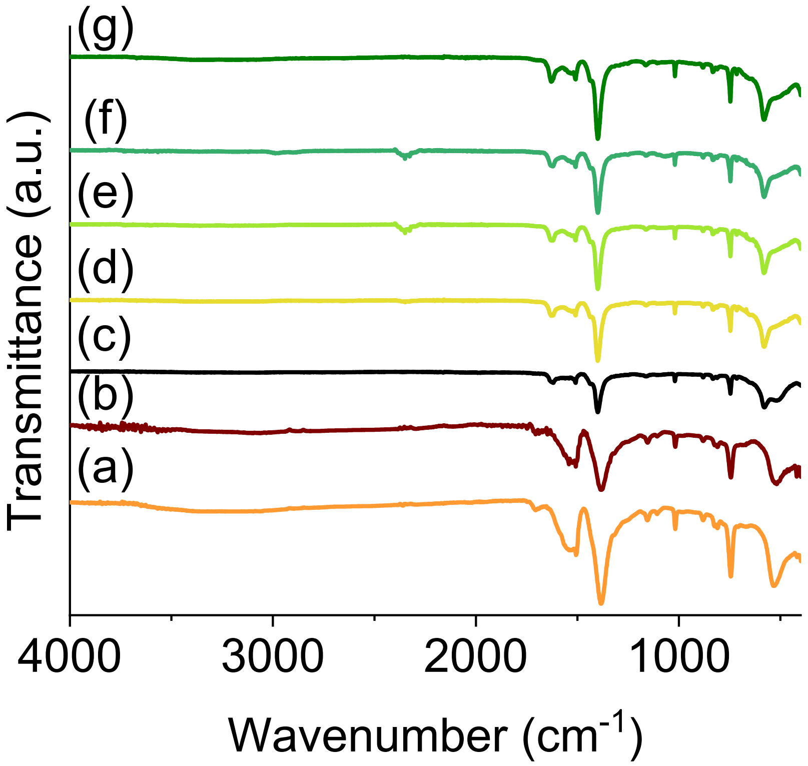


Figure S3. ATR FT-IR spectroscopy of (a) MIL-101(Fe), (b) $RuO_x$(2 wt%)@MIL-101(Fe), (c) MIL-101(Cr), (d) $RuO_x$(0.2 wt%)@MIL-101(Cr), (e) $RuO_x$(0.5 wt%)@MIL-101(Cr), (f) $RuO_x$(1 wt%)@MIL-101(Cr) and (g) $RuO_x$(2 wt%)@MIL-101(Cr).

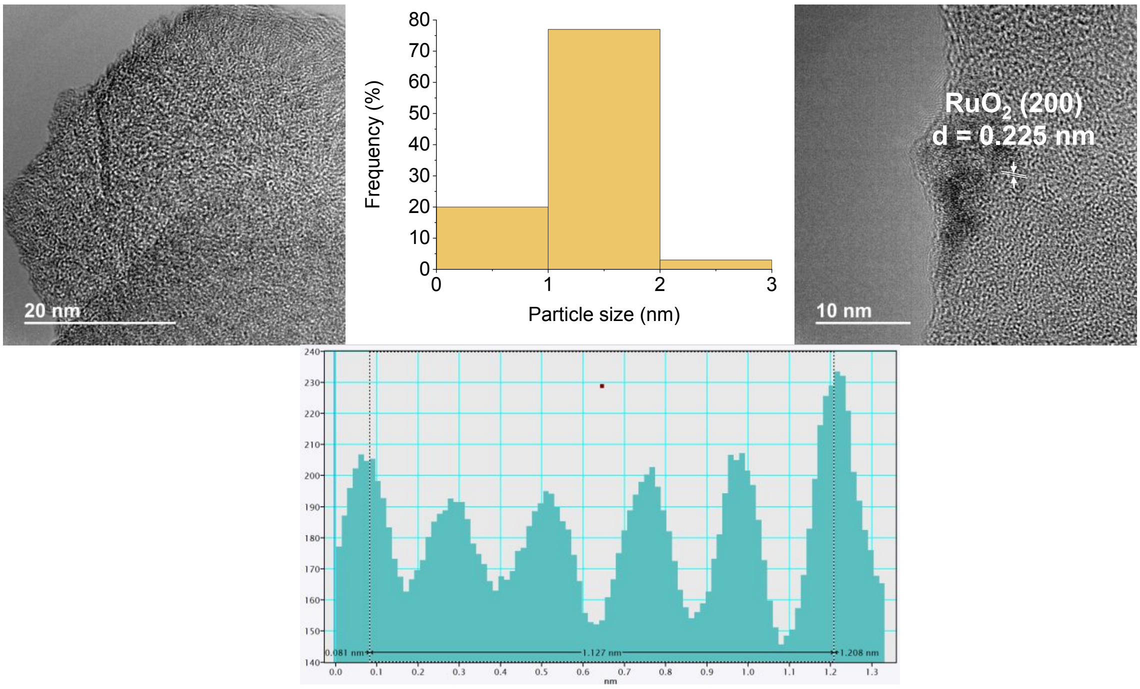


Figure S4. TEM images of used $RuO_x$(0.2 wt%)@MIL-101(Cr), $RuO_x$ NPs size distribution and interplane distance measurement. Average particle size is 1.25 ± 0.33 nm.

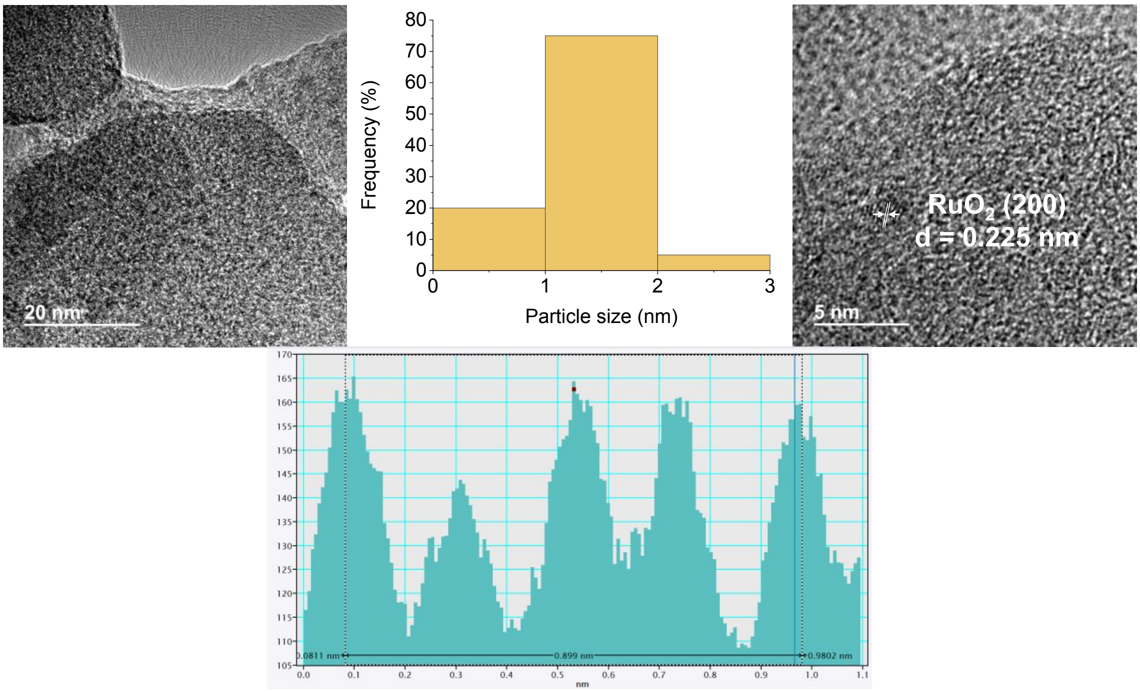

Figure S5. TEM images of used $RuO_x$(0.5 wt%)@MIL-101(Cr), $RuO_x$ NPs size distribution and interplane distance measurement. Average particle size is 1.30 ± 0.36 nm.

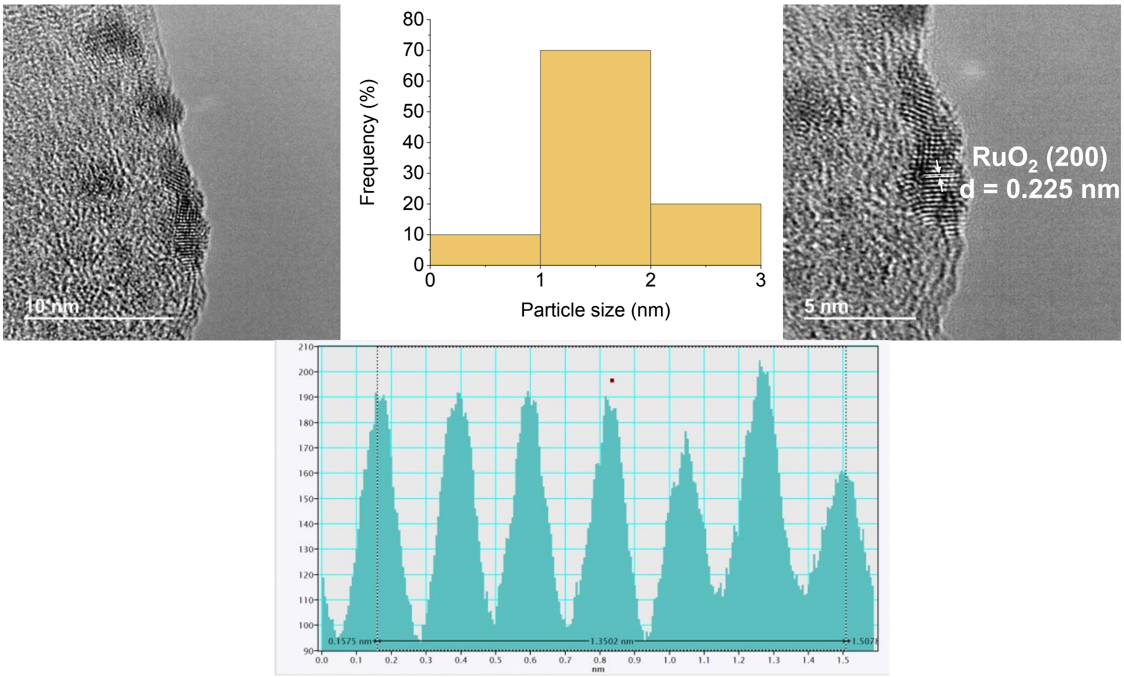


Figure S6. TEM images of used $RuO_x$(1 wt%)@MIL-101(Cr), $RuO_x$ NPs size distribution and interplane distance measurement. Average particle size is 1.53 ± 0.44 nm.

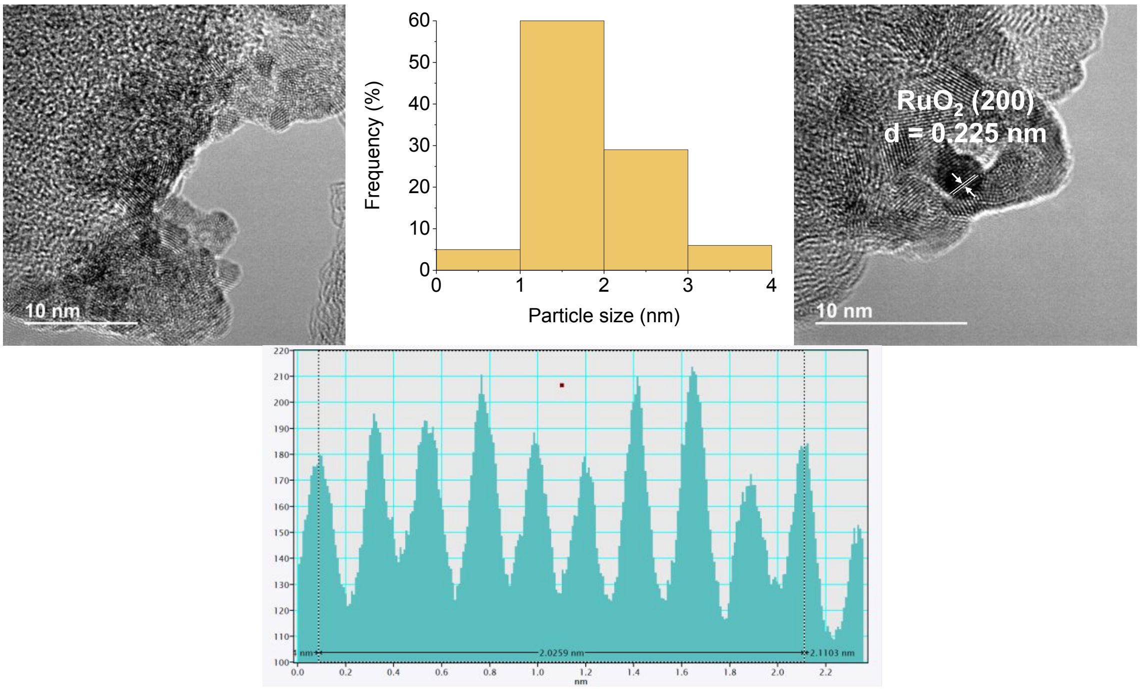

Figure S7. TEM images of $RuO_x$(2 wt%)@MIL-101(Cr), $RuO_x$ NPs size distribution and interplane distance measurement. Average particle size is 1.56 ± 0.51 nm.

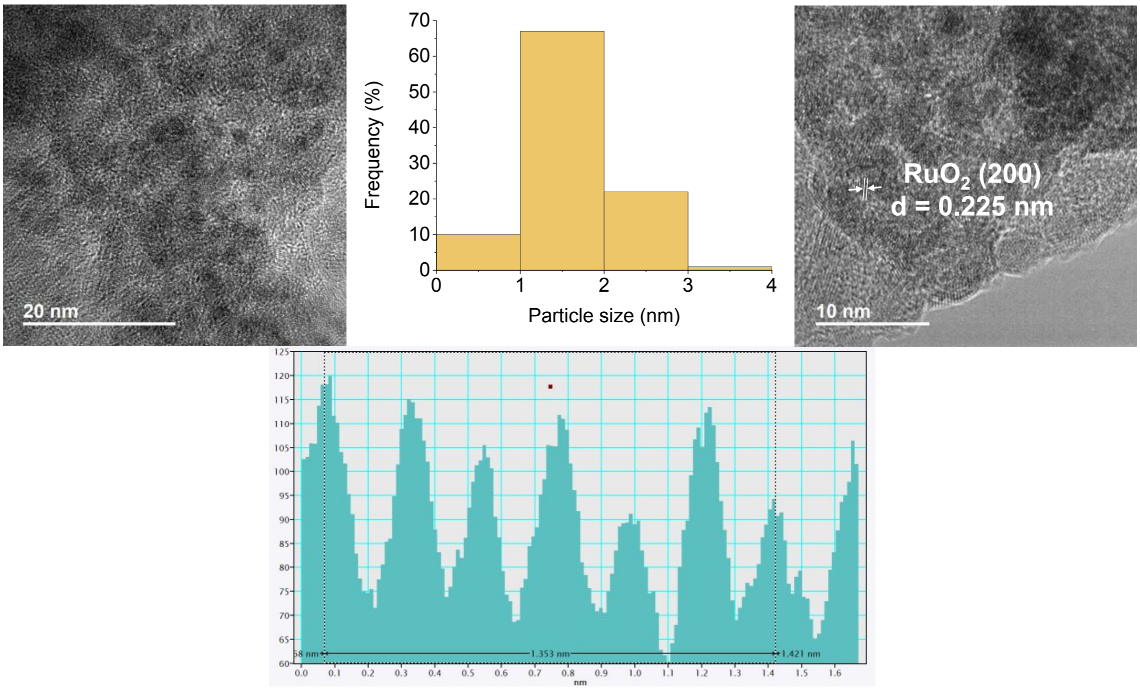


Figure S8. TEM images of $RuO_x$(2 wt%)@MIL-101(Fe), $RuO_x$ NPs size distribution and interplane distance measurement. Average particle size is 1.70 ± 0.52 nm.

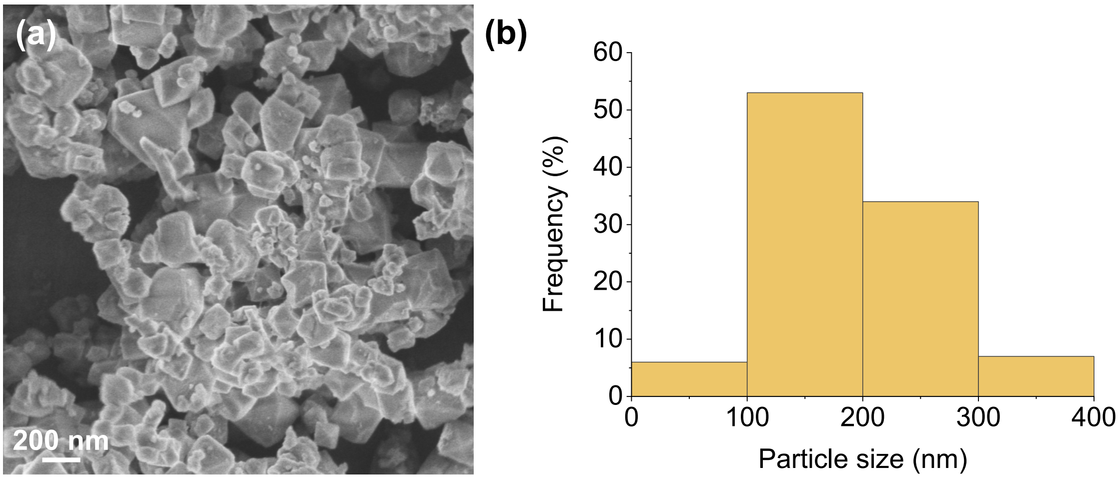


Figure S9. (a) SEM images and (b) particle size distribution of MIL-101(Cr). Average particle size is 192.3 ± 63.1 nm.

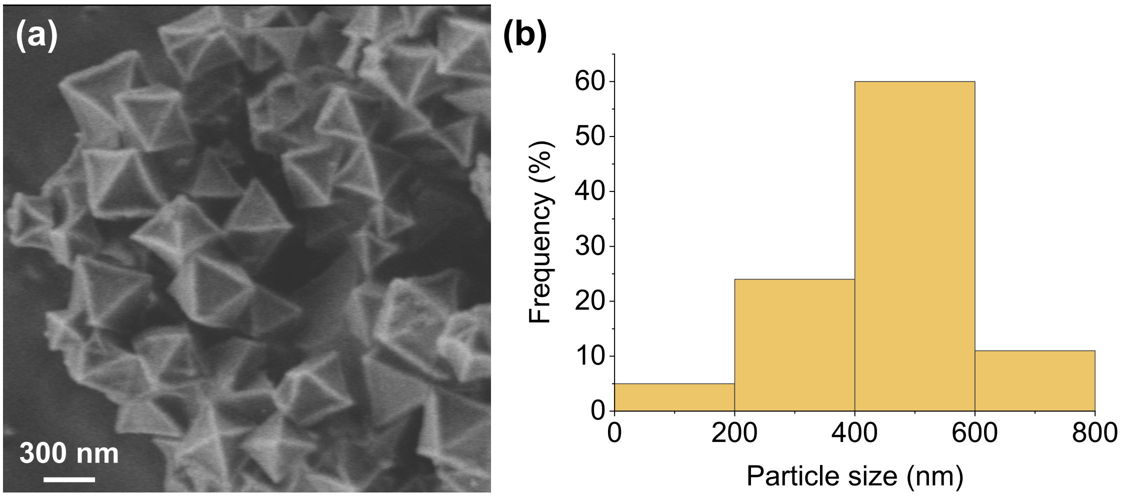


Figure S10. (a) SEM images and (b) particle size distribution of MIL-101(Fe). Average particle size is 451.4 ± 128.0 nm.

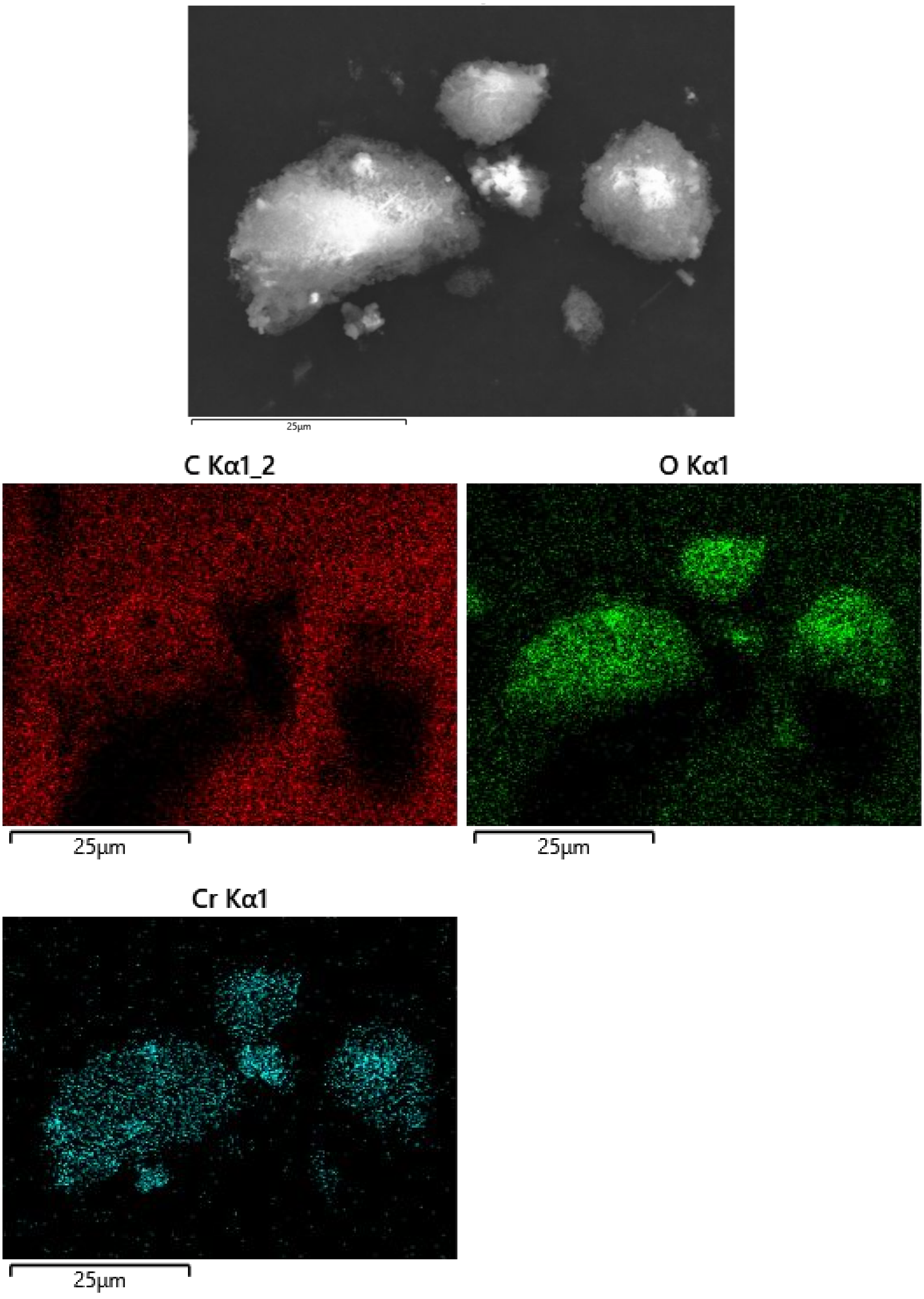


Figure S11. SEM-EDX of MIL-101(Cr).

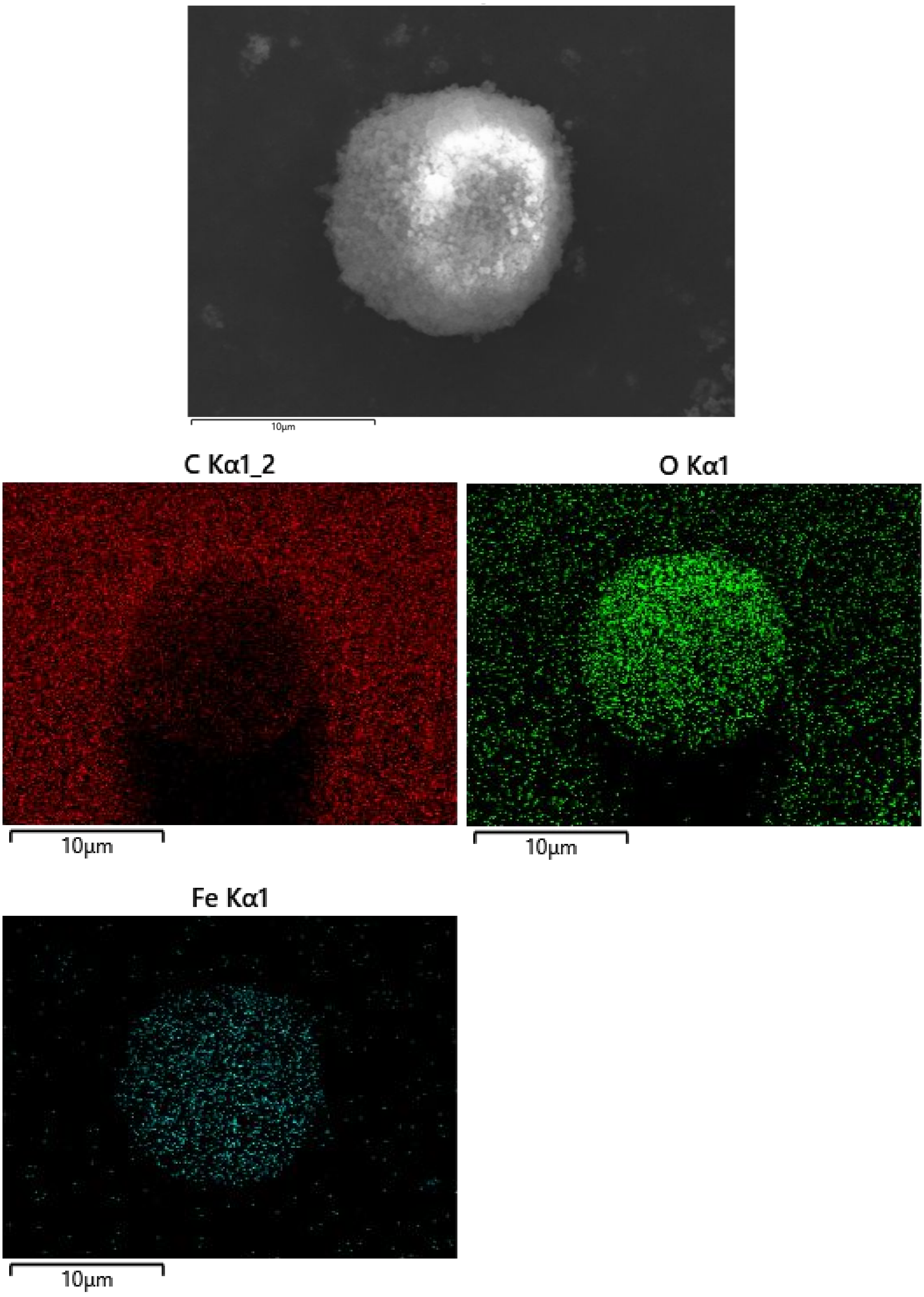


Figure S12. SEM-EDX of MIL-101(Fe).

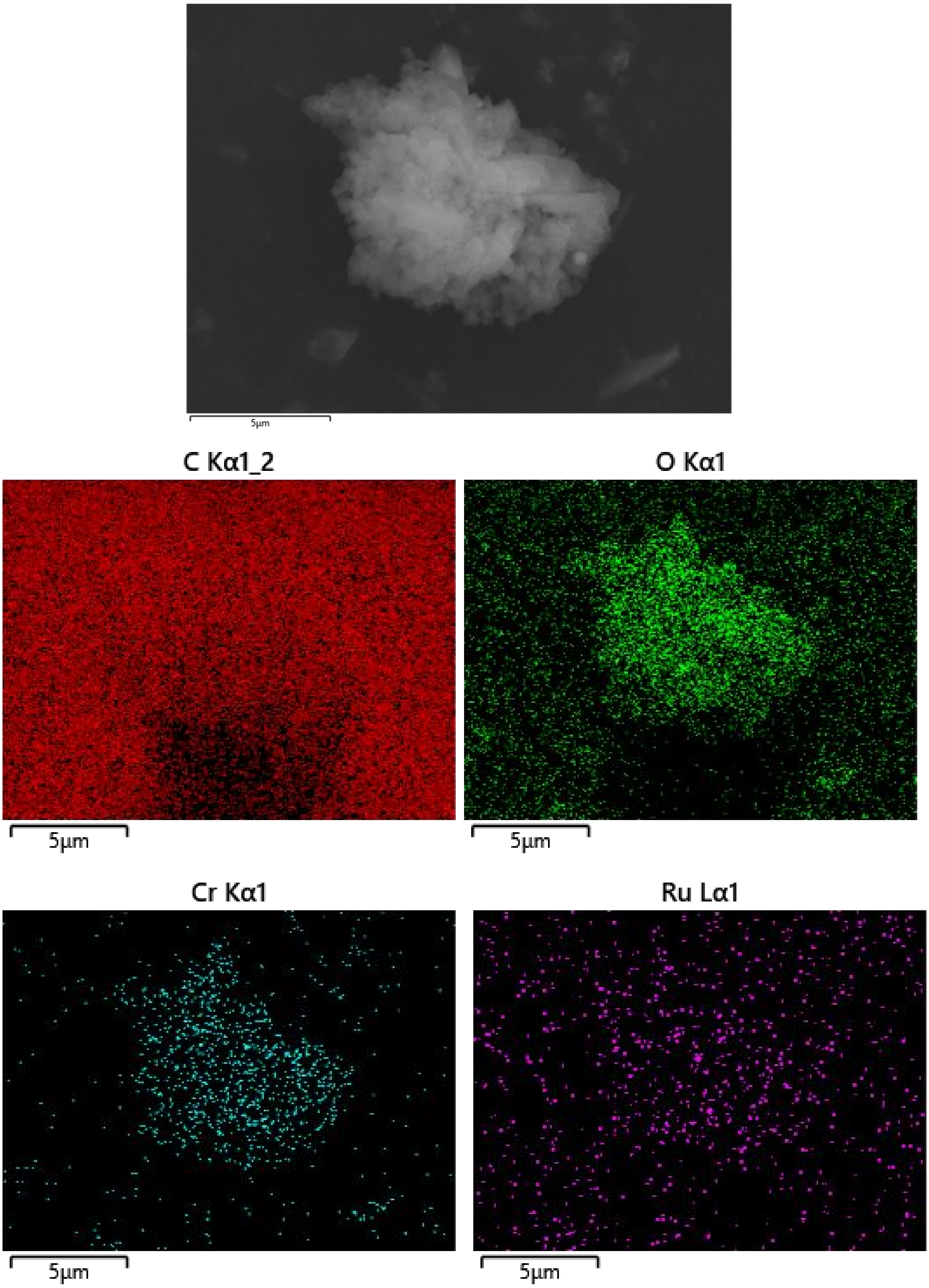


Figure S13. SEM-EDX of $RuO_x$(2 wt%)@MIL-101(Cr).

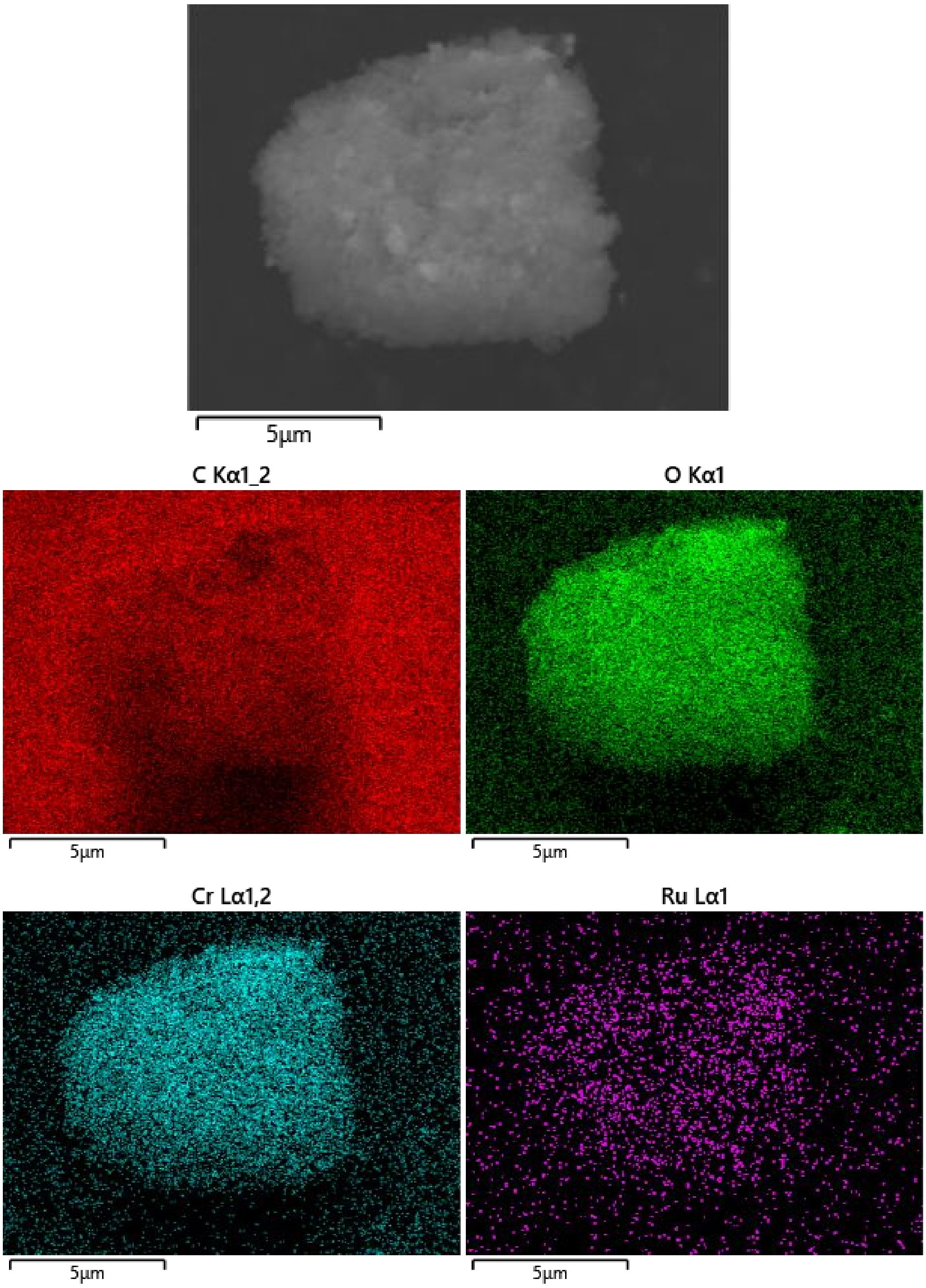


Figure S14. SEM-EDX of $RuO_x$(1 wt%)@MIL-101(Cr).

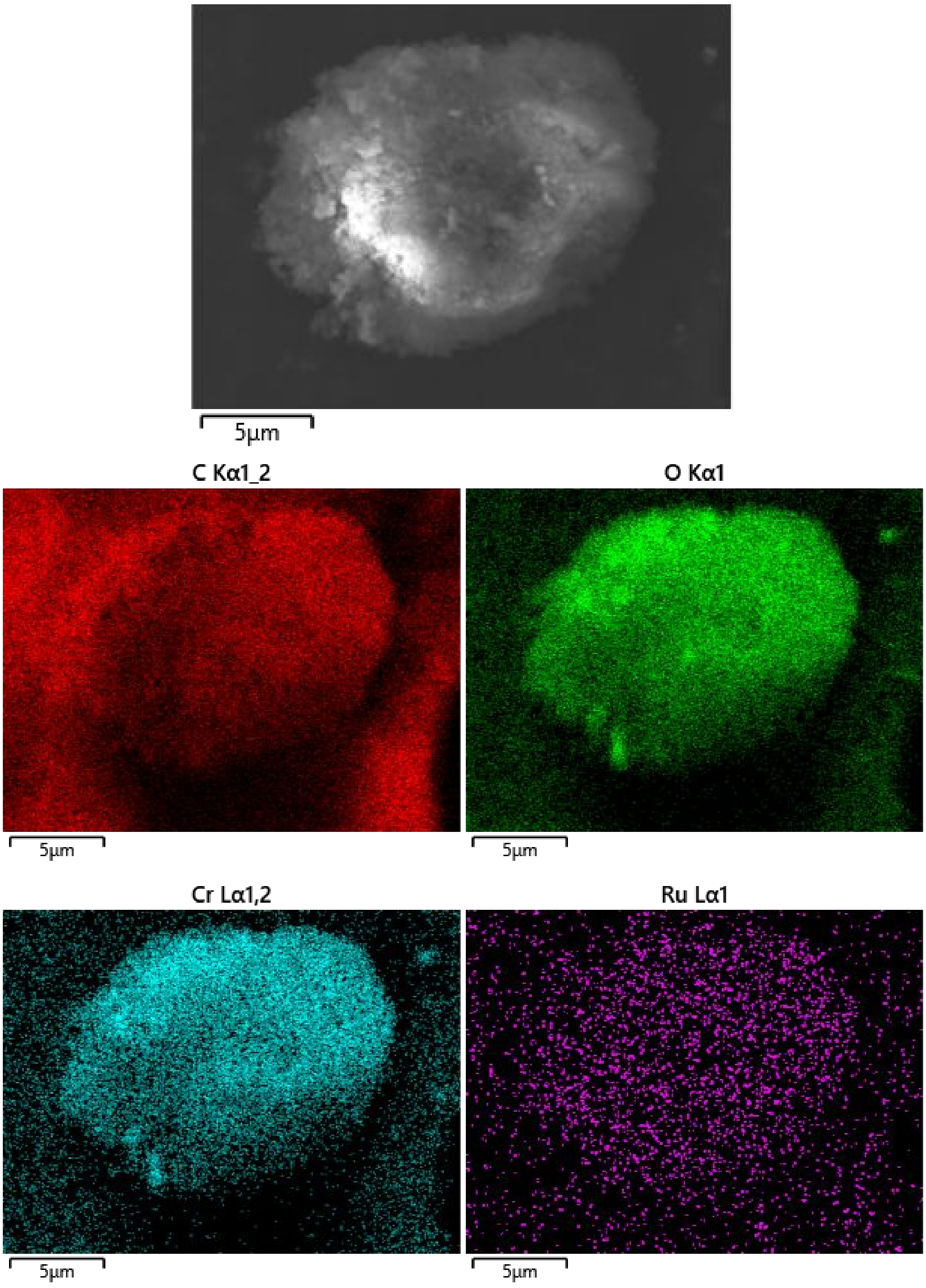


Figure S15. SEM-EDX of $RuO_x$(0.5 wt%)@MIL-101(Cr).

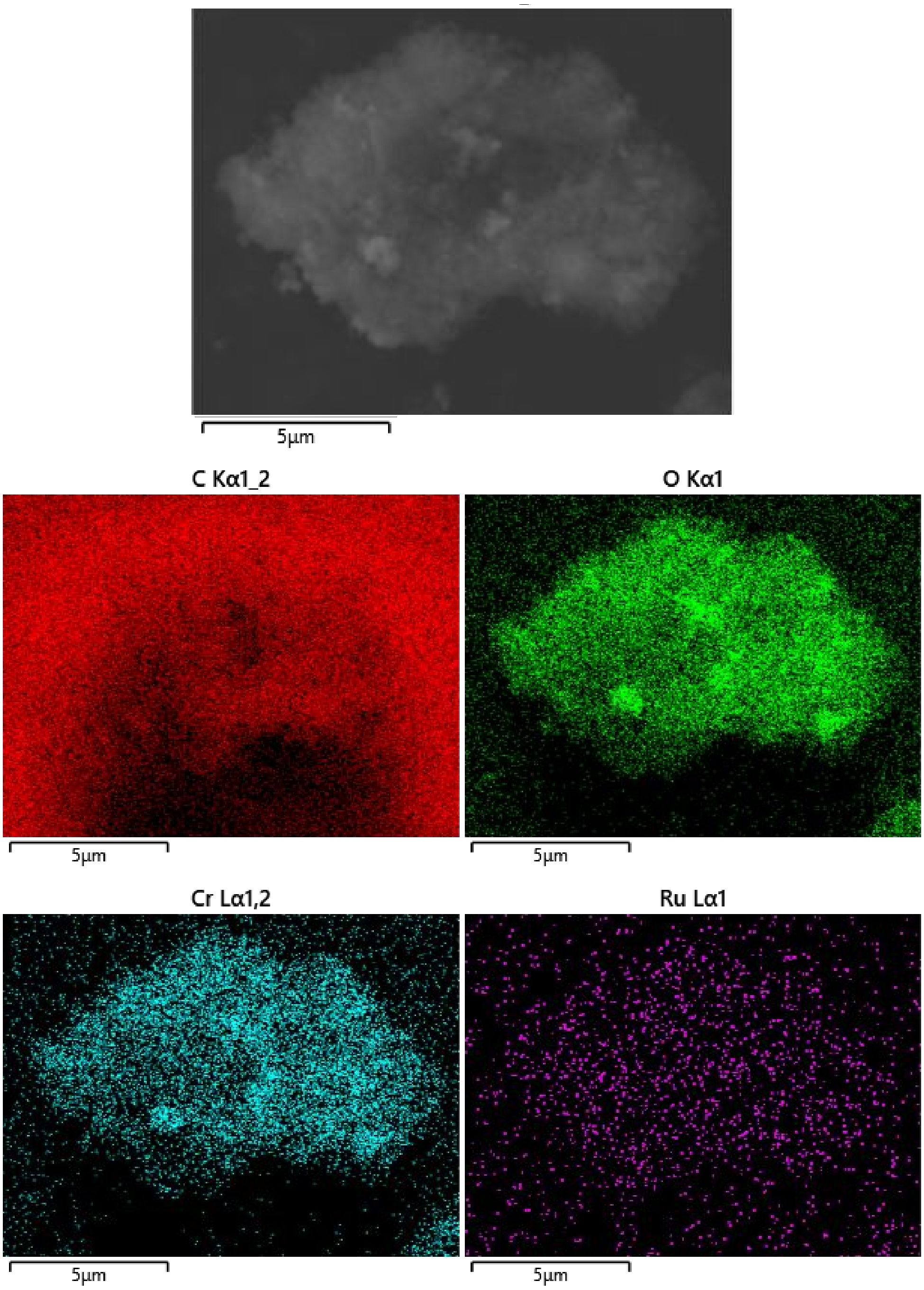


Figure S16. SEM-EDX of $RuO_x$(0.2 wt%)@MIL-101(Cr).

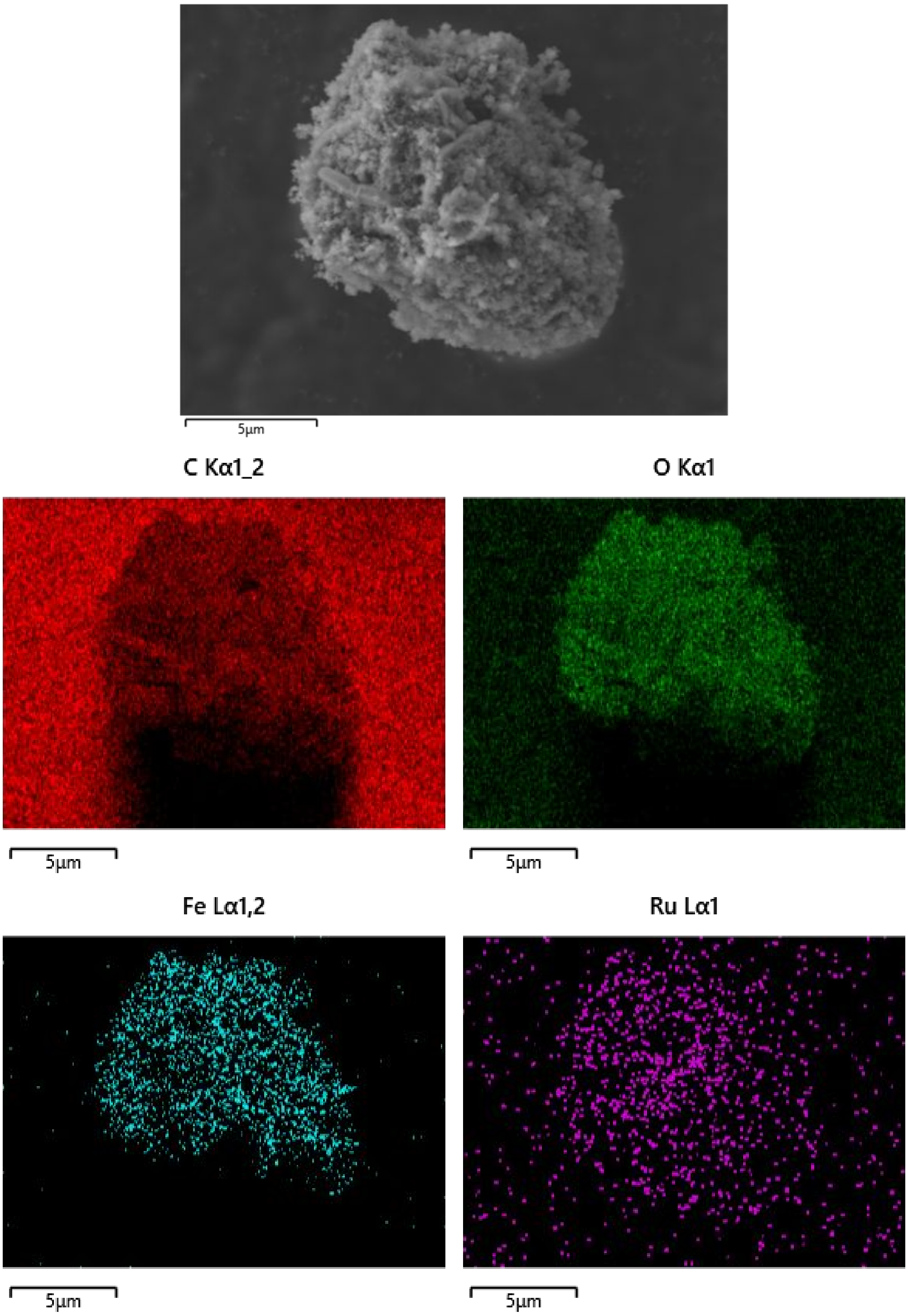


Figure S17. SEM-EDX of $RuO_x$(2 wt%)@MIL-101(Fe).

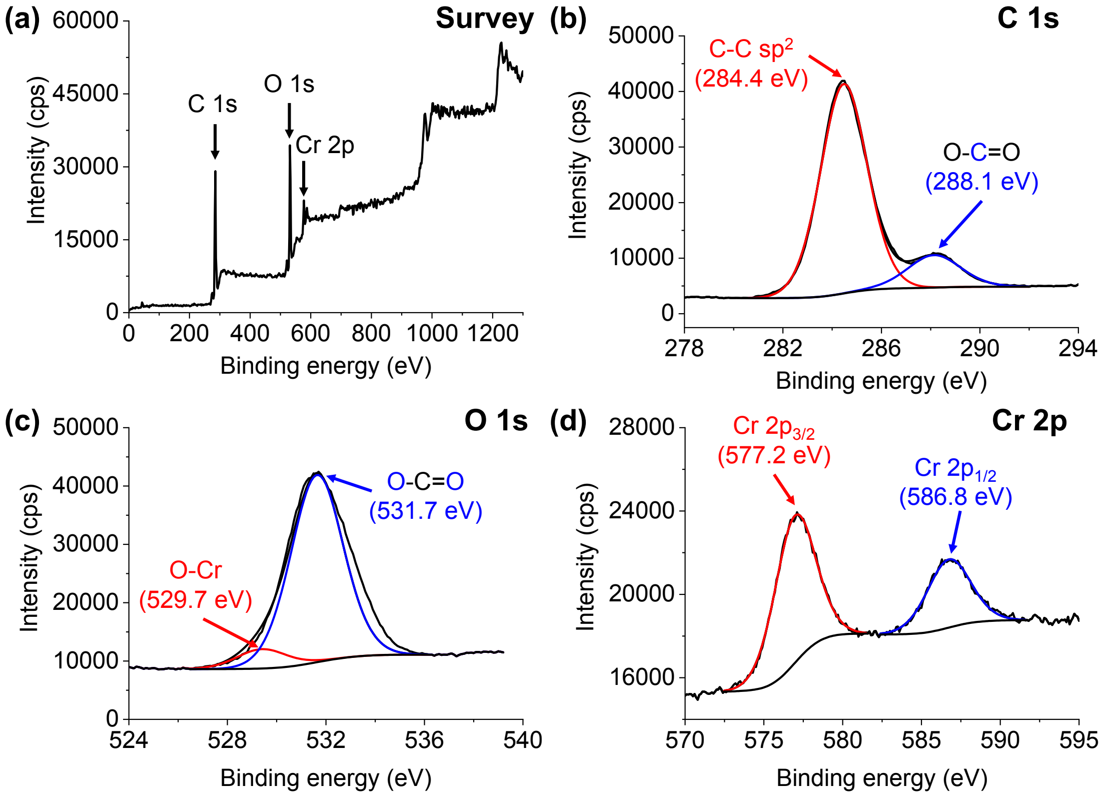


Figure S18. XPS (a) Survey, (b) C 1s, (c) O 1s and (d) Cr 2p of MIL-101(Cr).

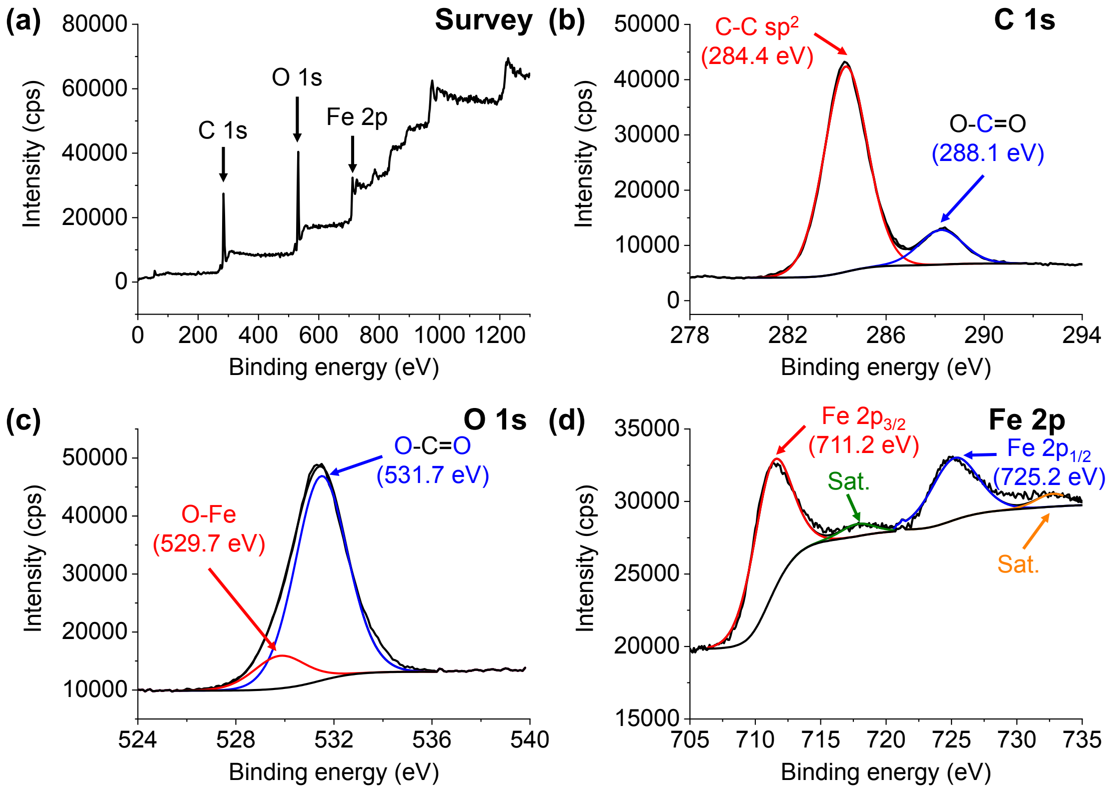


Figure S19. XPS (a) Survey, (b) C 1s, (c) O 1s and (d) Fe 2p of MIL-101(Fe).

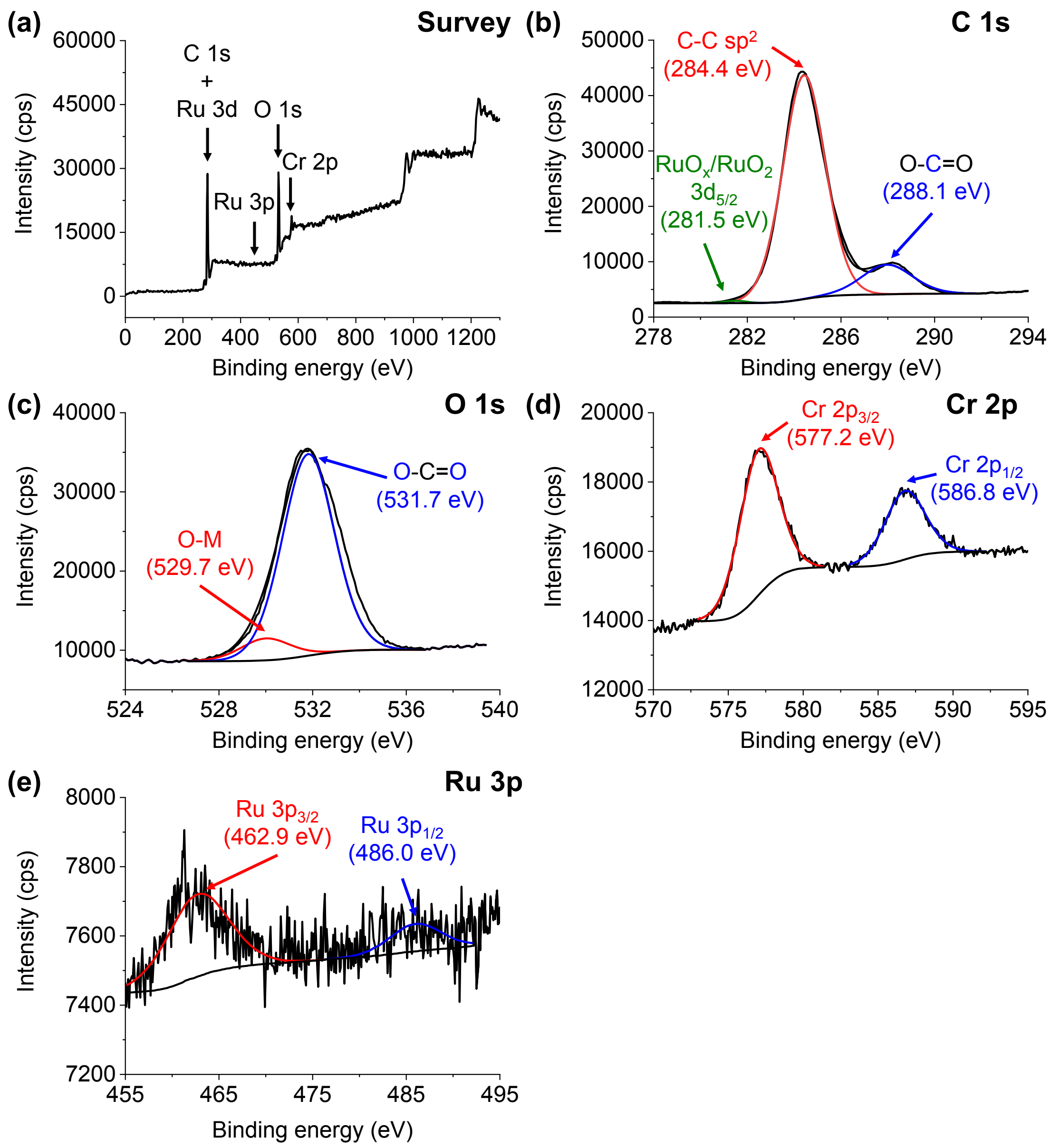


Figure S20. XPS (a) Survey, (b) C 1s, (c) O 1s, (d) Cr 2p and (e) Ru 3p for $RuO_x$(0.2 wt%)@MIL-101(Cr).

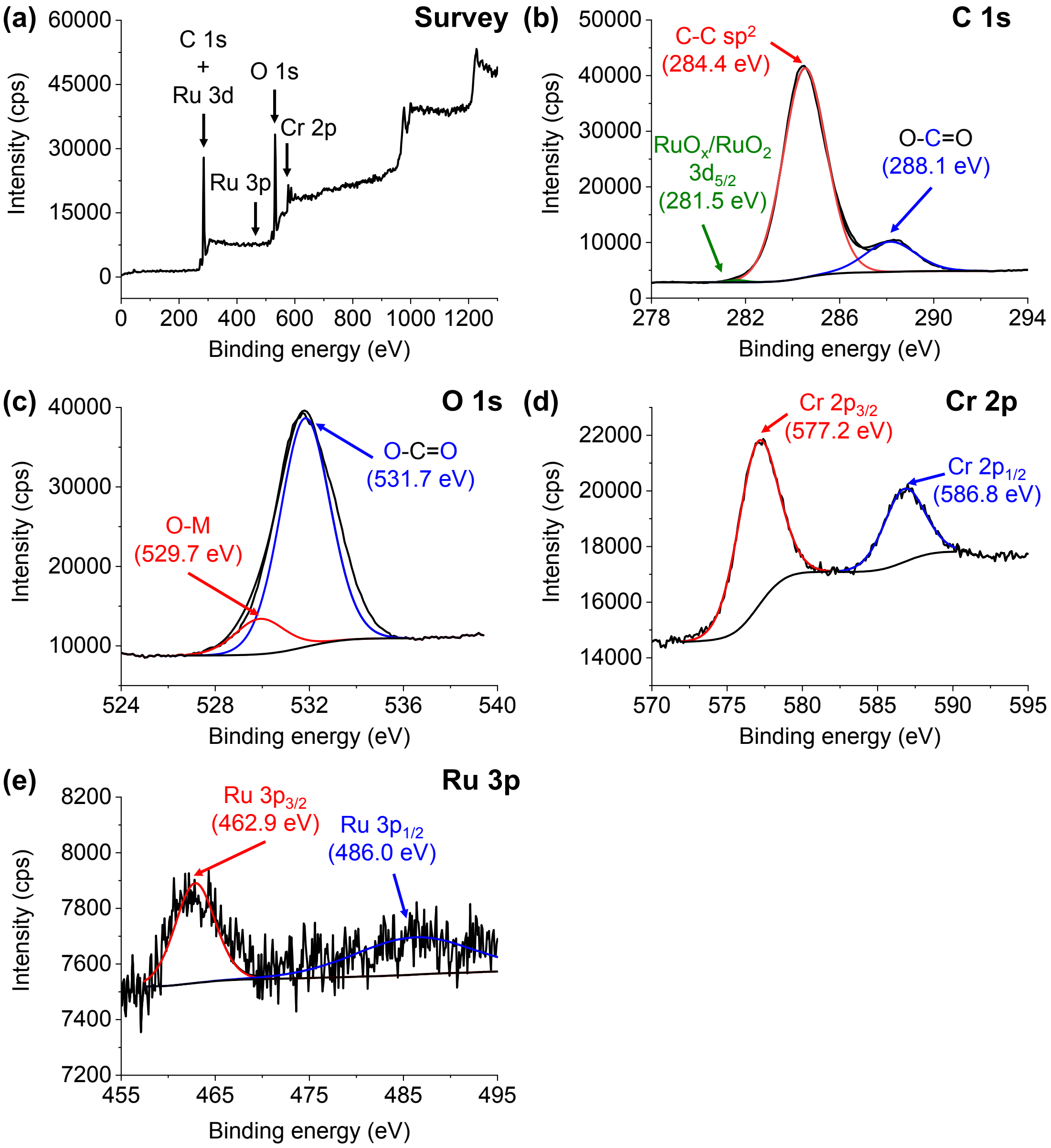


Figure S21. XPS (a) Survey, (b) C 1s, (c) O 1s, (d) Cr 2p and (e) Ru 3p for $RuO_x$(0.5 wt%)@MIL-101(Cr).

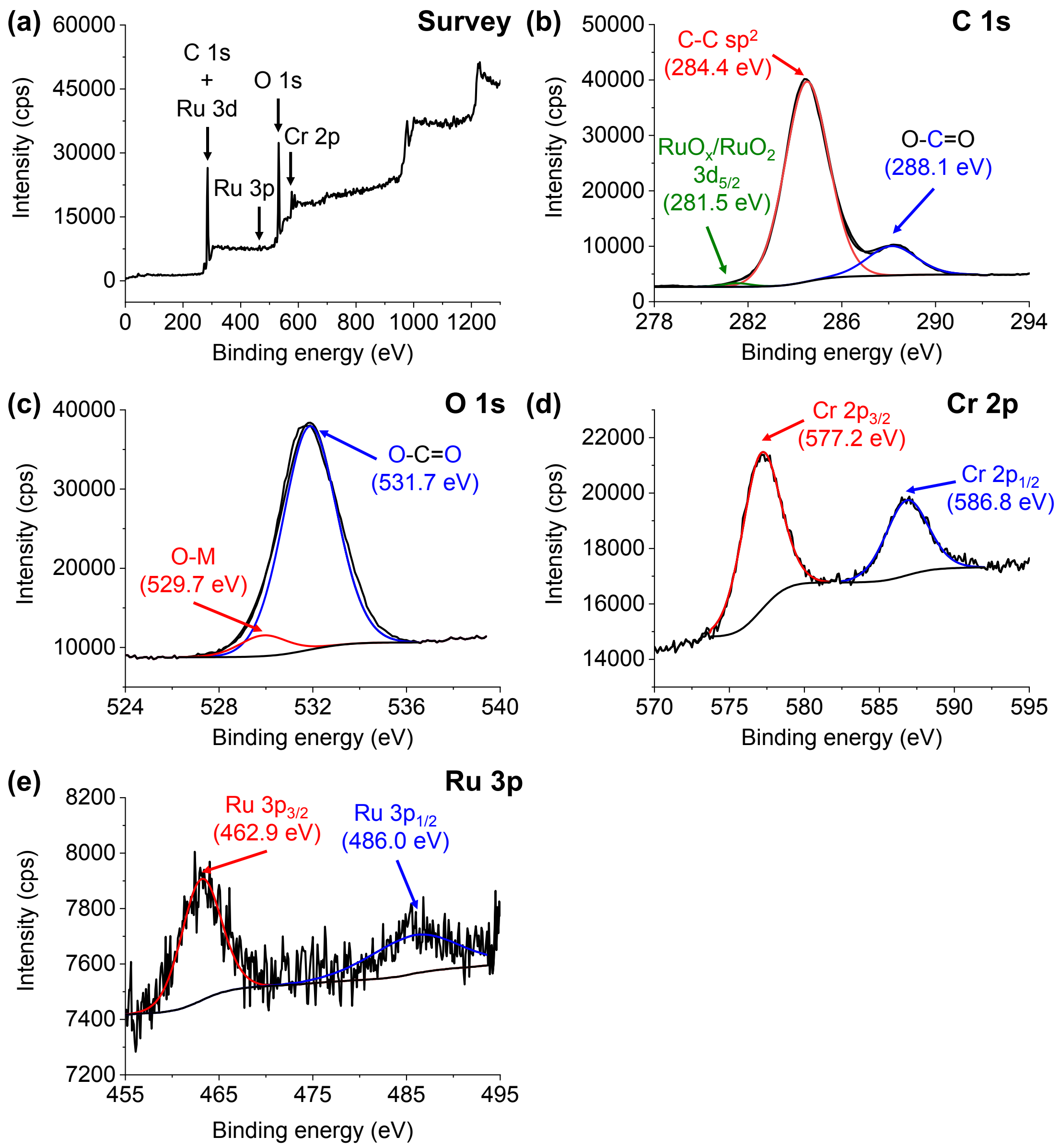


Figure S22. XPS (a) Survey, (b) C 1s, (c) O 1s, (d) Cr 2p and (e) Ru 3p for $RuO_x$(1 wt%)@MIL-101(Cr).

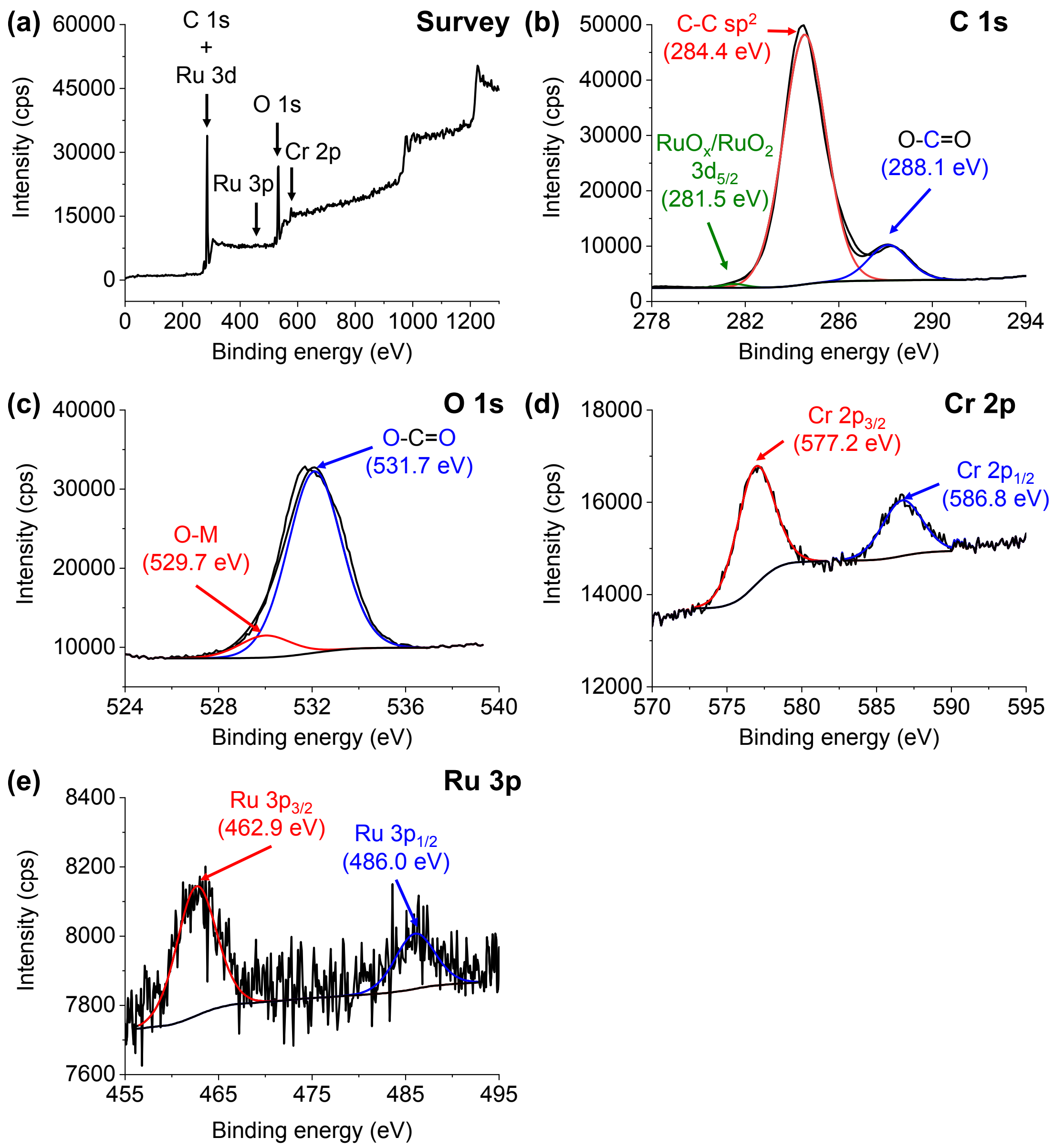


Figure S23. XPS (a) Survey, (b) C 1s, (c) O 1s, (d) Cr 2p and (e) Ru 3p for $RuO_x$(2 wt%)@MIL-101(Cr).

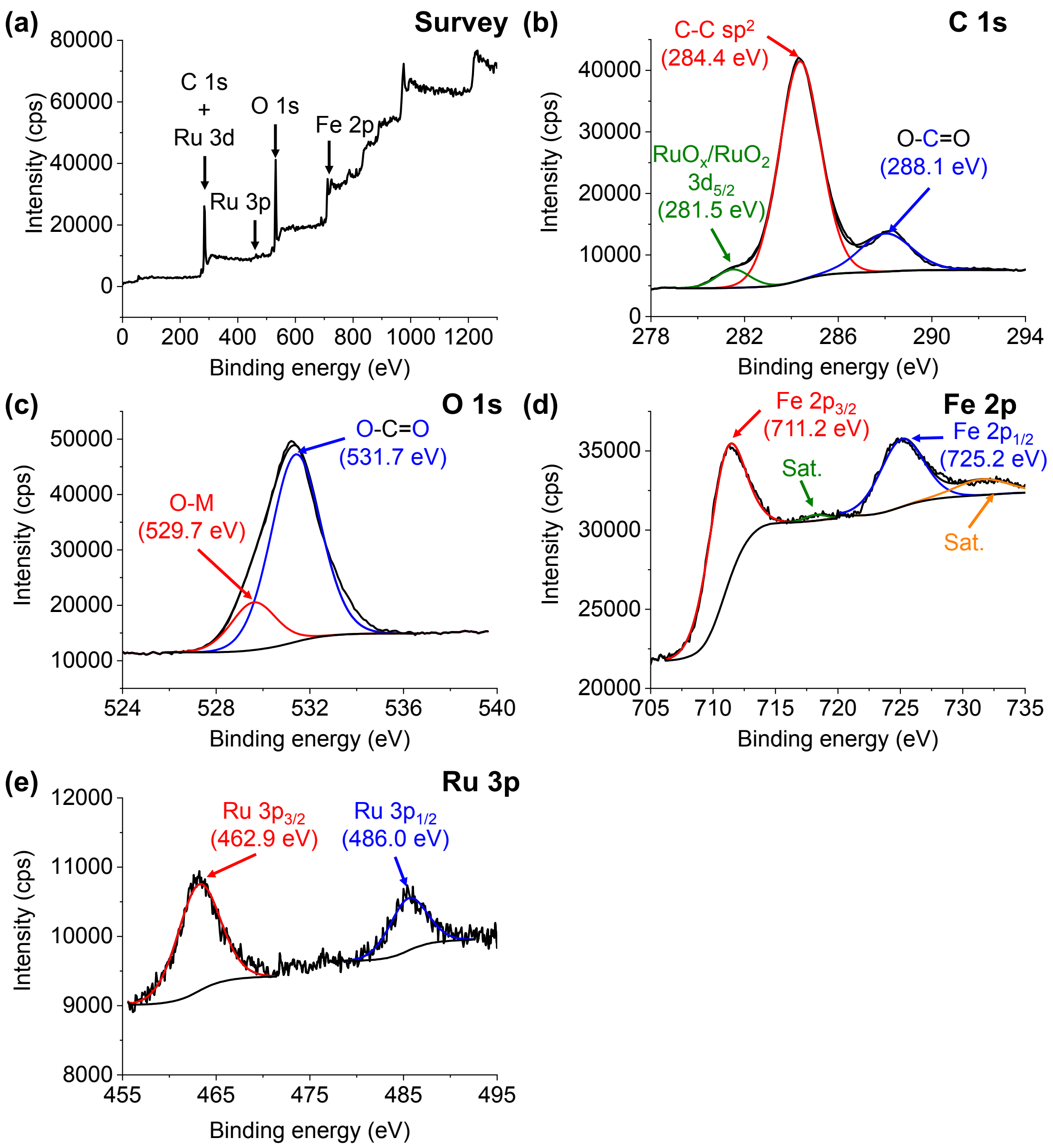


Figure S24. XPS (a) Survey, (b) C 1s, (c) O 1s, (d) Fe 2p and (e) Ru 3p for $RuO_x$(2 wt%)@MIL-101(Fe).

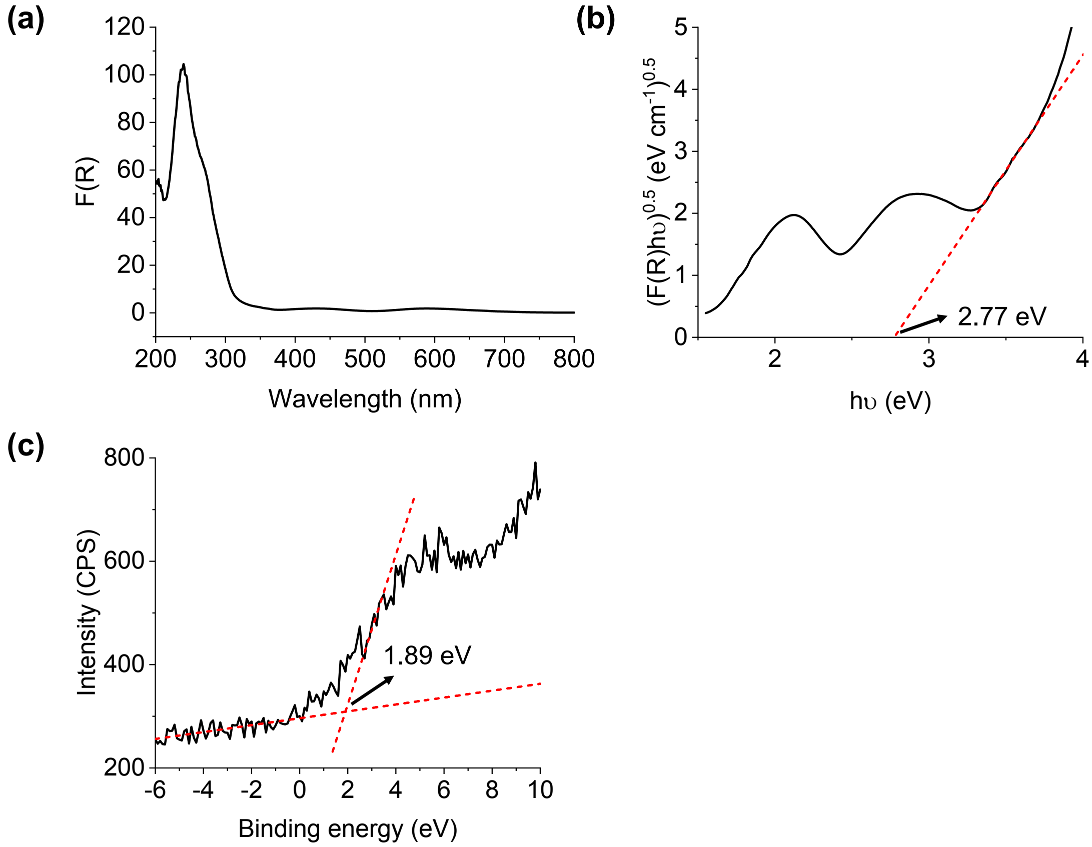


Figure S25. (a) UV-Visible diffuse reflectance spectra of (a1) MIL-101(Cr), (a2) $RuO_x$(2 wt%)@MIL-101(Cr), (b) Tauc plot and (c) valence band of MIL-101(Cr). Note: The bands at 420 and 620 nm are due to $Cr^{3+}$ d-d transition and have not been considered to estimate optical band gap of MIL-101(Cr).

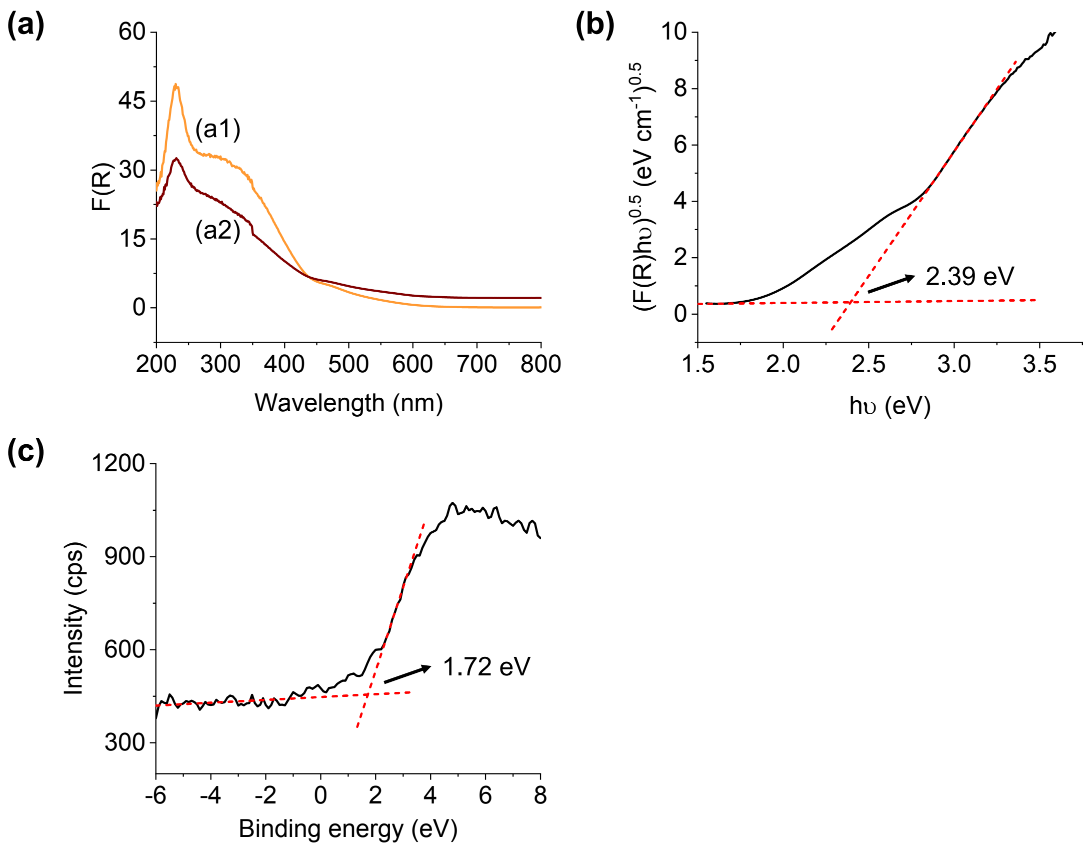


Figure S26. (a) UV-Visible diffuse reflectance spectra of (a1) MIL-101(Fe), (a2) $RuO_x$(2 wt%)@MIL-101(Fe), (b) Tauc plot and (c) valence band of MIL-101(Fe).

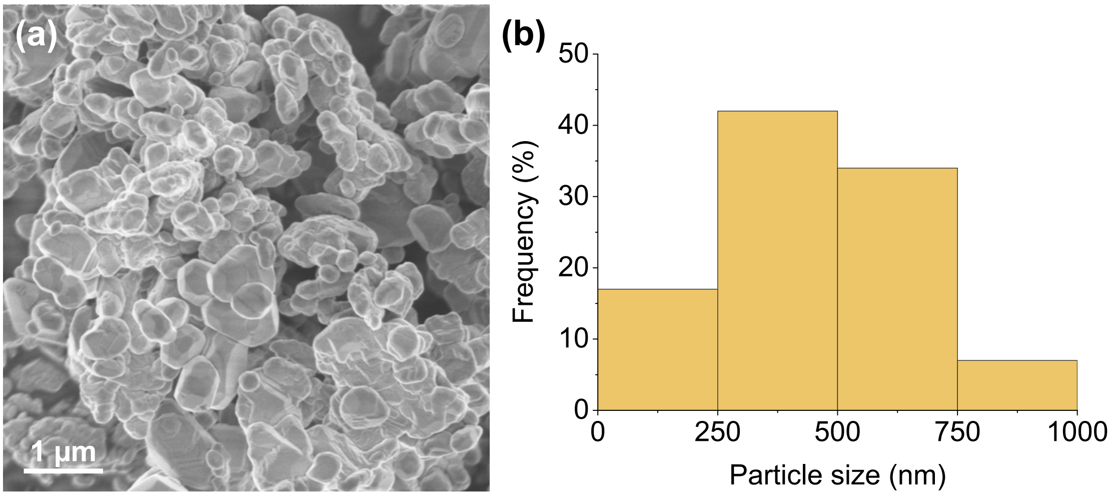


Figure S27. (a) SEM images and (b) particle size distribution of $Cr_2O_3$. Average particle size is 458.8 ± 200.6 nm.

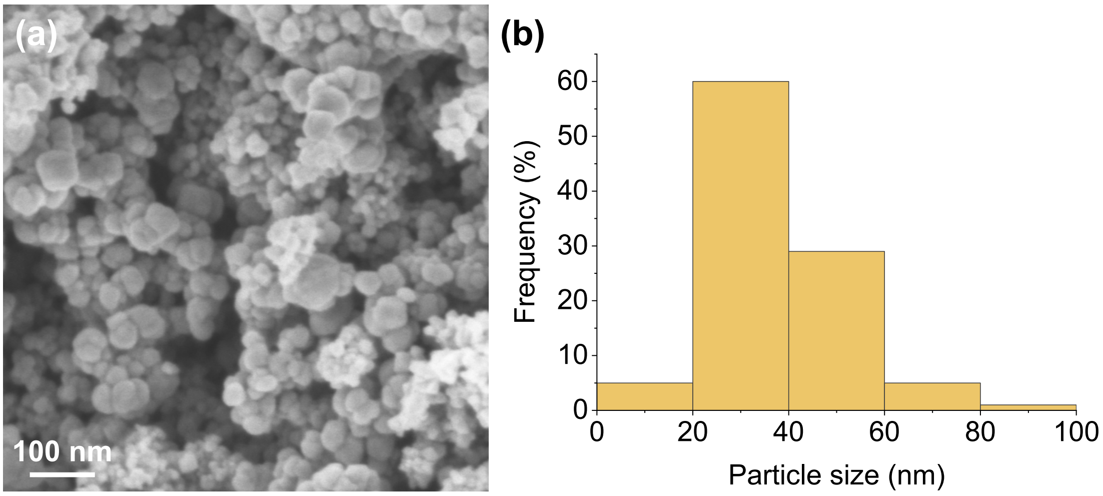


Figure S28. (a) SEM images of $\gamma$-$Fe_2O_3$ and (b) particle size distribution. Average particle size is 36.9 ± 13.8 nm.

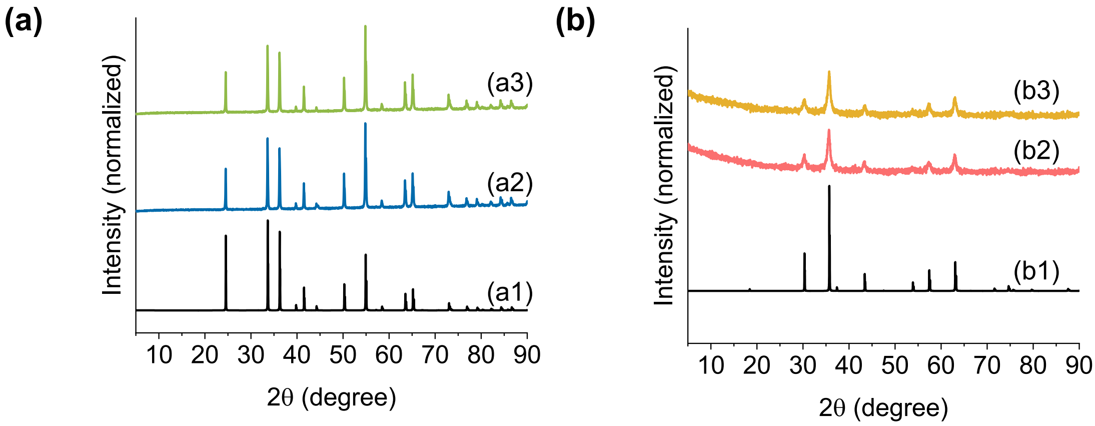


Figure S29. PXRD of (a1) simulated $Cr_2O_3$, (a2) $Cr_2O_3$, (a3) $RuO_x$(2 wt%)@$Cr_2O_3$ and (b1) simulated γ-$Fe_2O_3$, (b2) γ-$Fe_2O_3$, (b3) $RuO_x$(2 wt%)@ γ-$Fe_2O_3$.

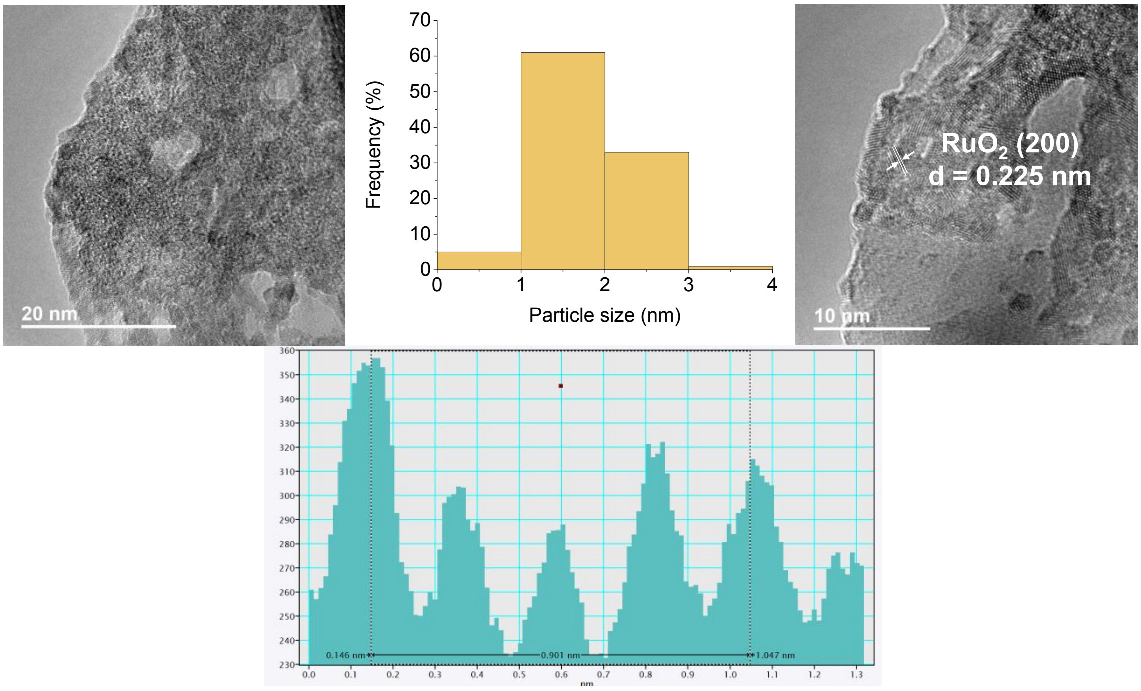


Figure S30. TEM images of $RuO_x$(2 wt%)@$Cr_2O_3$, $RuO_x$ NPs size distribution and interplane distance measurement. Average particle size is 1.79 ± 0.49 nm.

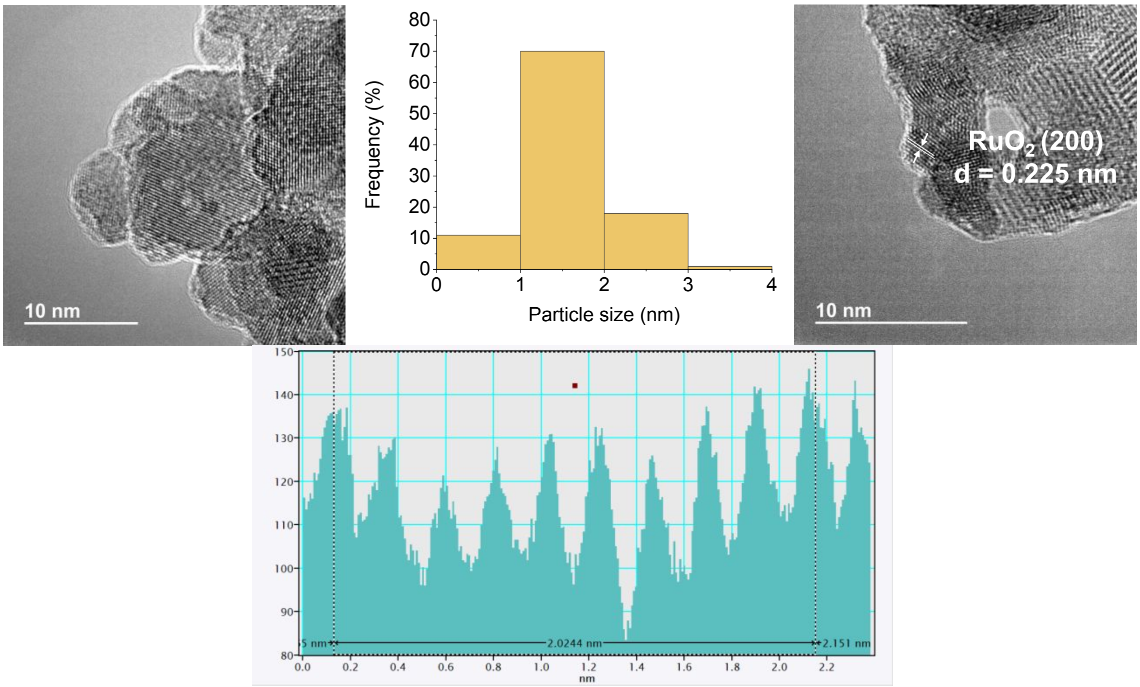


Figure S31. TEM images of $RuO_x$(2 wt%)@γ-$Fe_2O_3$, $RuO_x$ NPs size distribution and interplane distance measurement. Average particle size is 1.56 ± 0.53 nm.

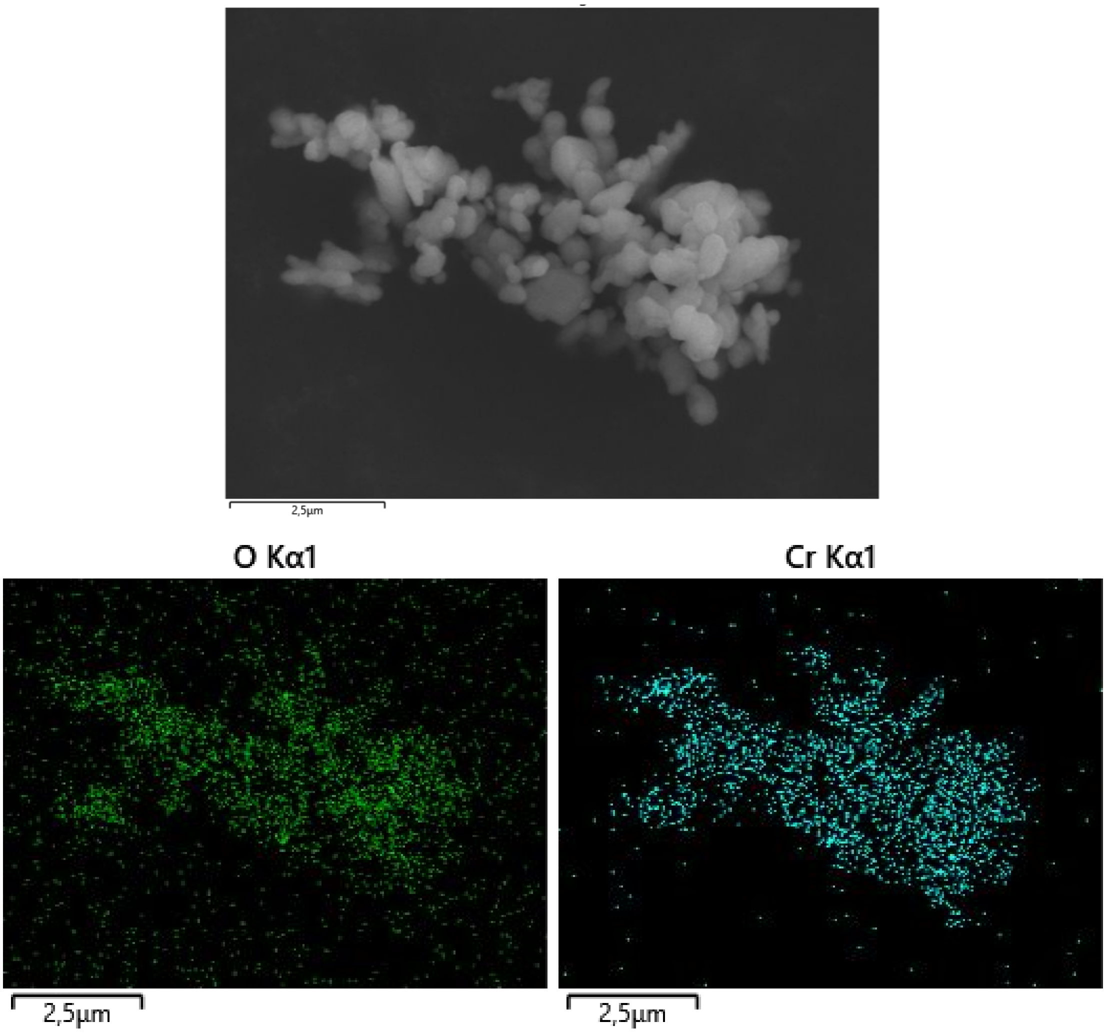


Figure S32. SEM-EDX of $Cr_2O_3$.

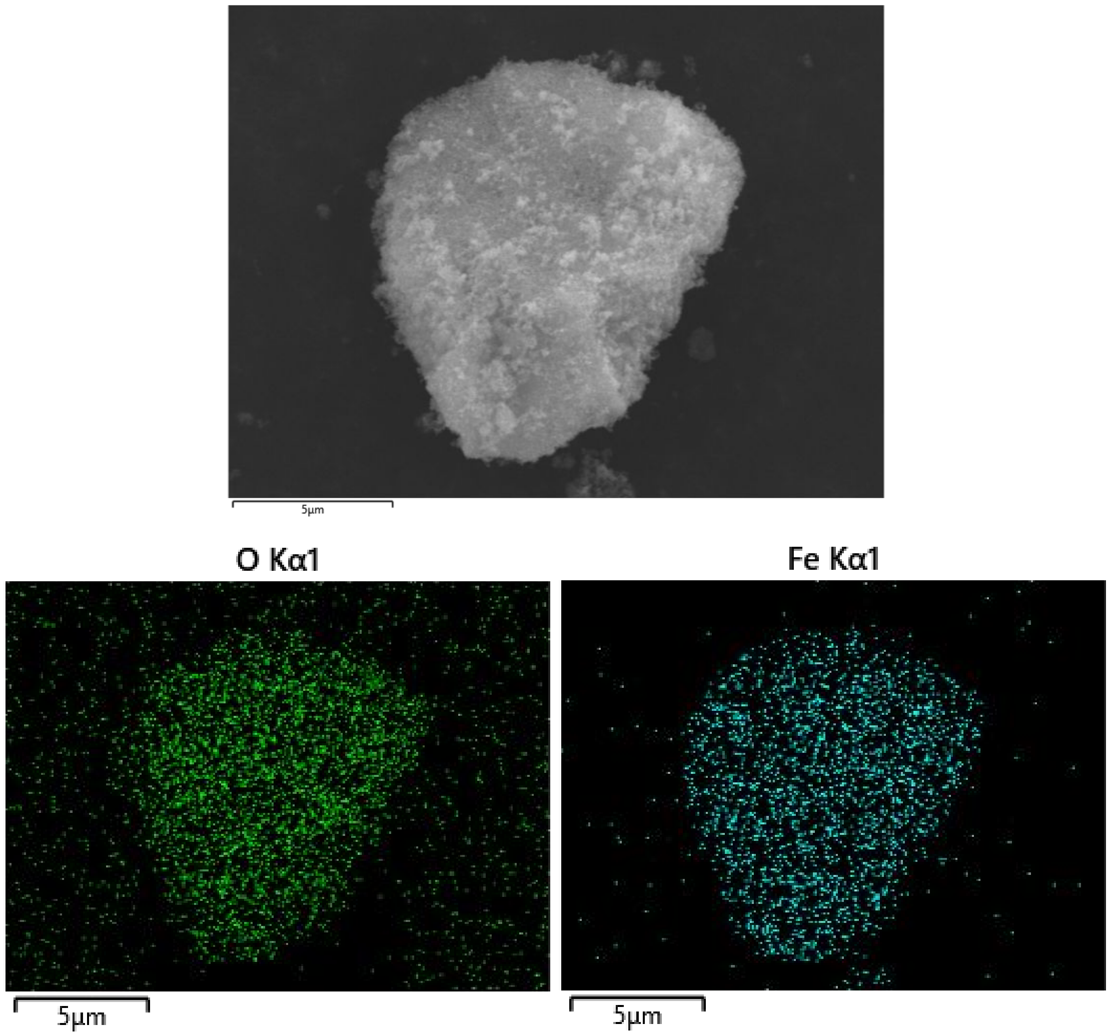


Figure S33. SEM-EDX of γ-$Fe_2O_3$.

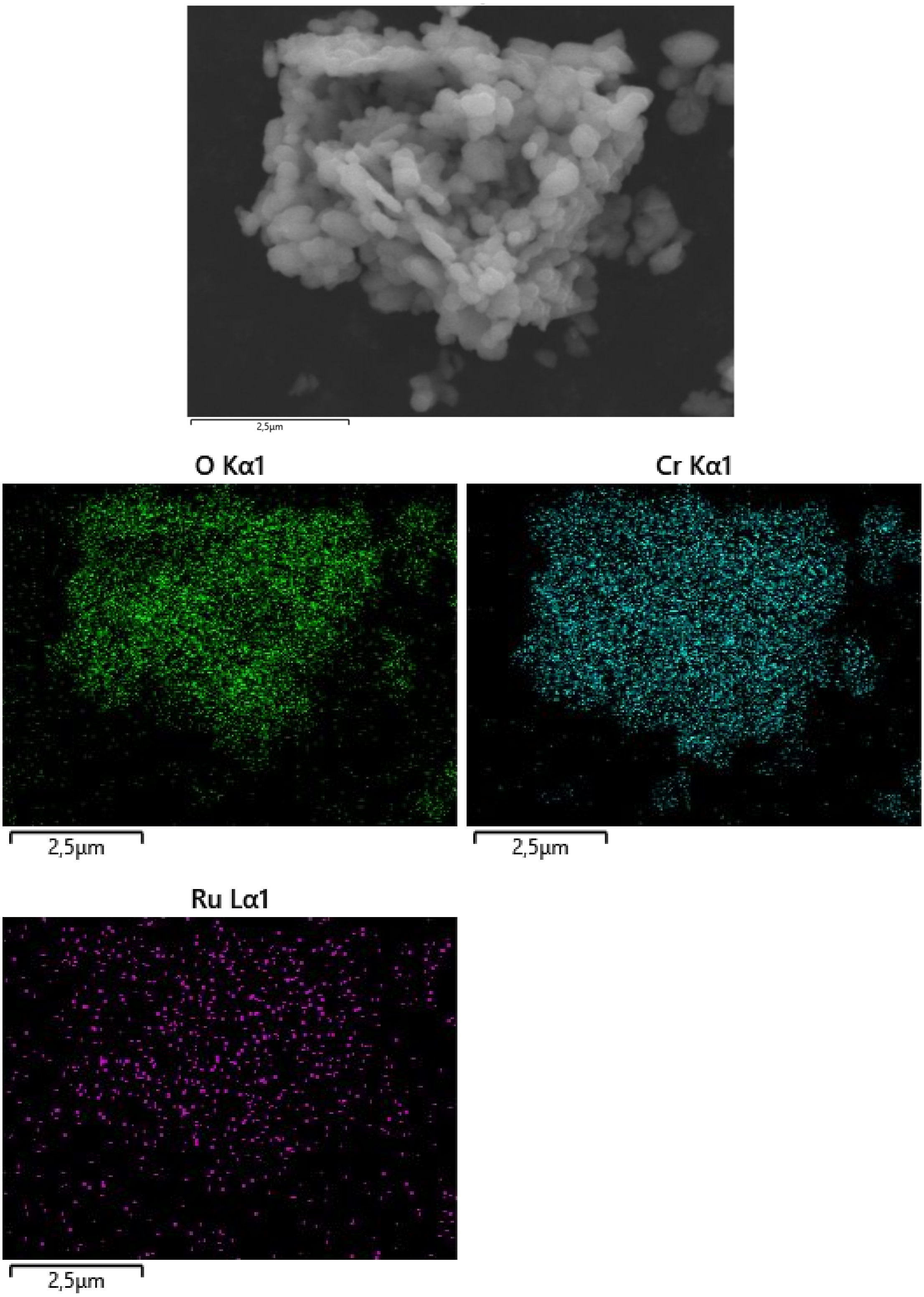


Figure S34. SEM-EDX of $RuO_x$(2 wt%)@$Cr_2O_3$.

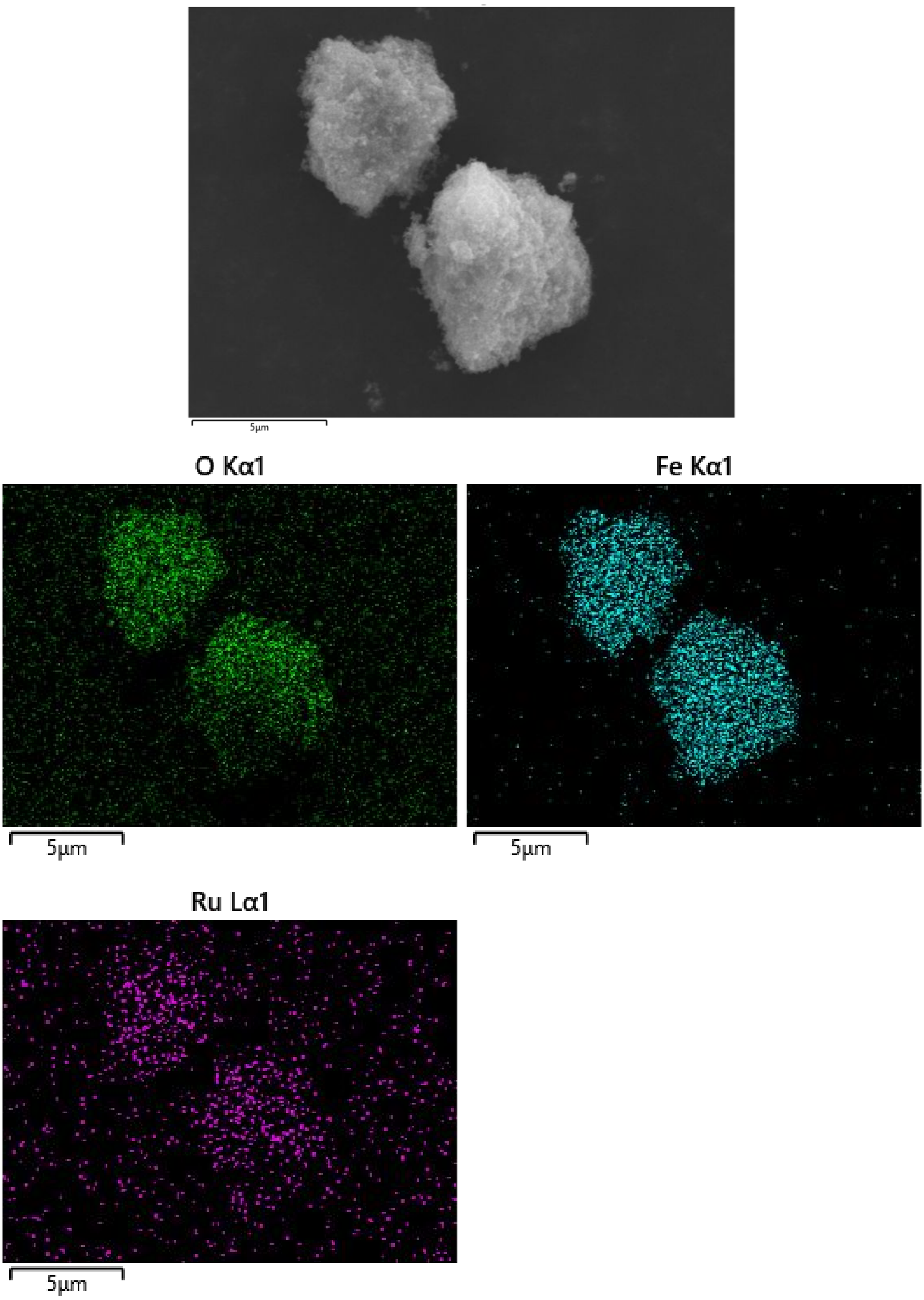


Figure S35. SEM-EDX of $RuO_x$(2 wt%)@γ-$Fe_2O_3$.

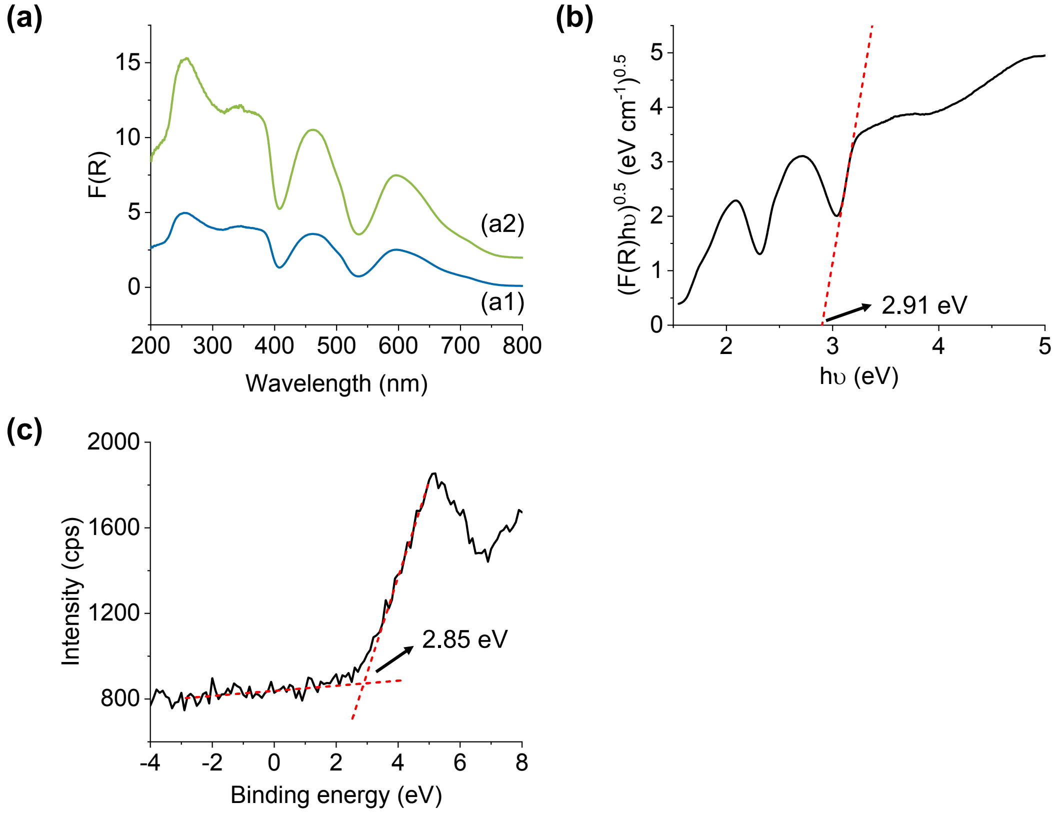


Figure S36. (a) UV-Visible diffuse reflectance spectra of (a1) $Cr_2O_3$, (a2) $RuO_x$(2 wt%)@$Cr_2O_3$, (b) Tauc plot and (c) valence band of $Cr_2O_3$.

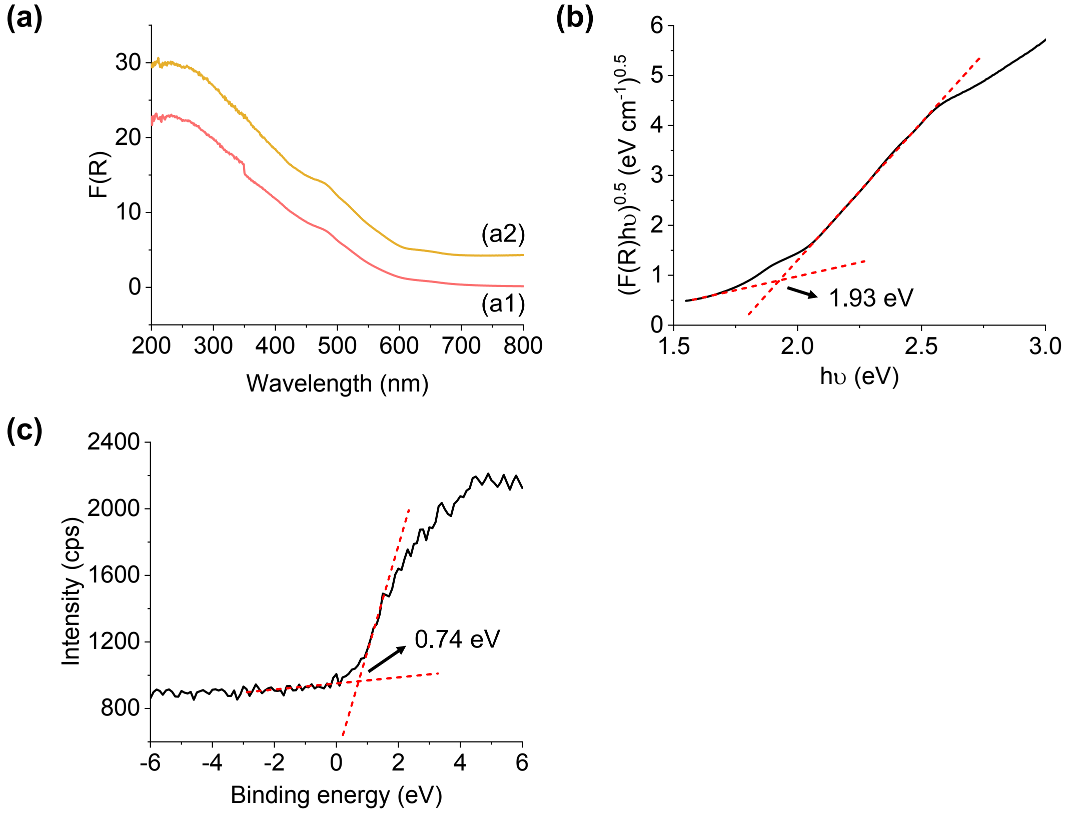


Figure S37. (a) UV-Visible diffuse reflectance spectra of (a1) γ-$Fe_2O_3$, (a2) $RuO_x$(2 wt%)@ γ-$Fe_2O_3$, (b) Tauc plot and (c) valence band of γ-$Fe_2O_3$.

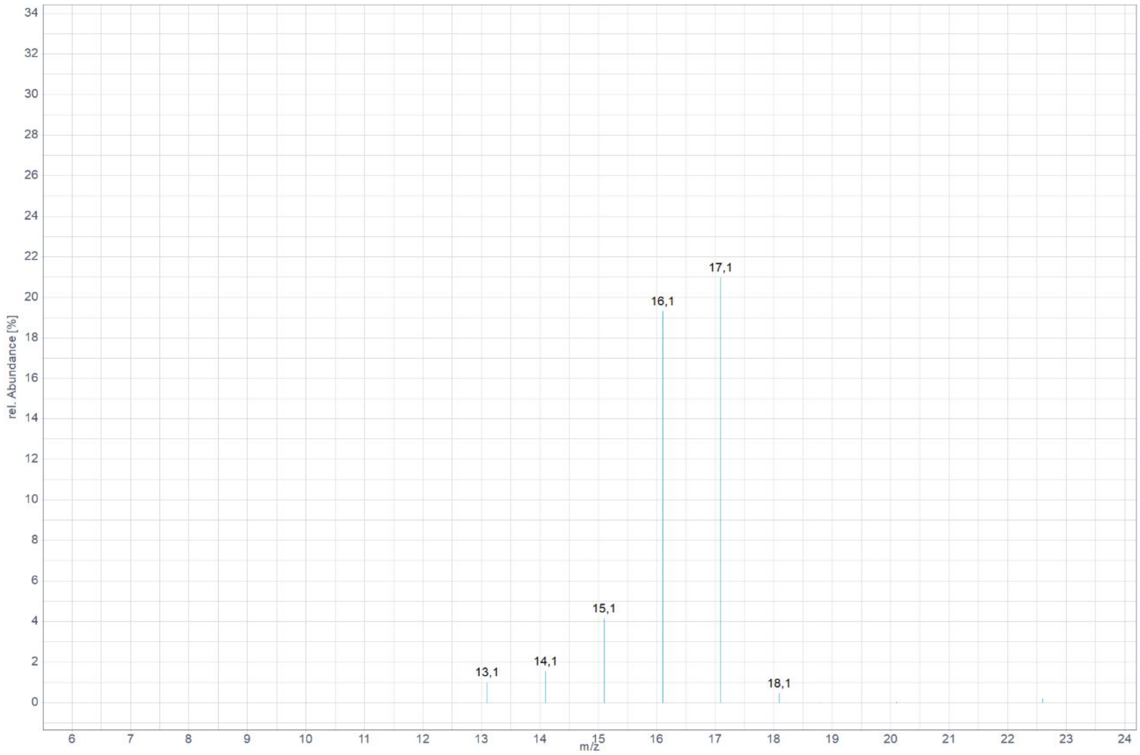


Figure S38. GC-MS result after photocatalytic $^{13}CO_2$ reduction using $RuO_x$(2 wt%)@MIL-101(Cr).

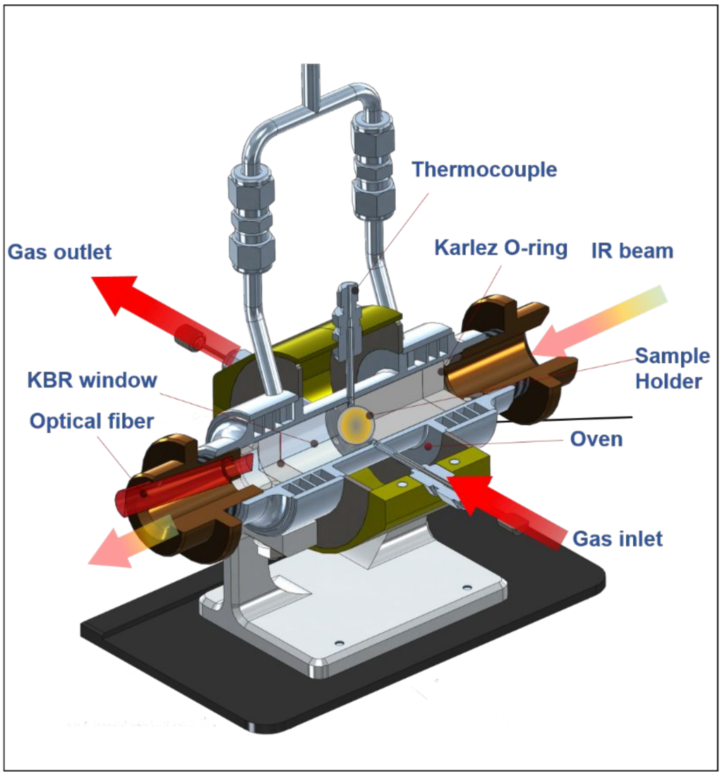


**Scheme S1.** IR sandwich cell reactor for the operando FTIR photocatalytic $CO_2$ methanation reaction.

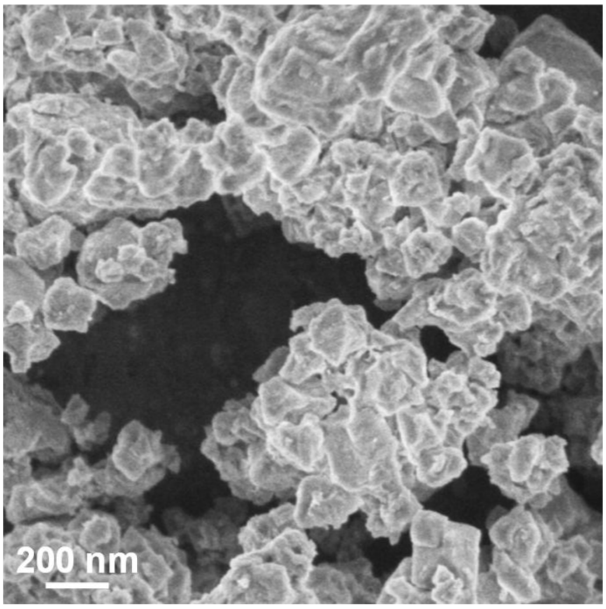


Figure S39. SEM of used $RuO_x$(2 wt%)@MIL-101(Cr).

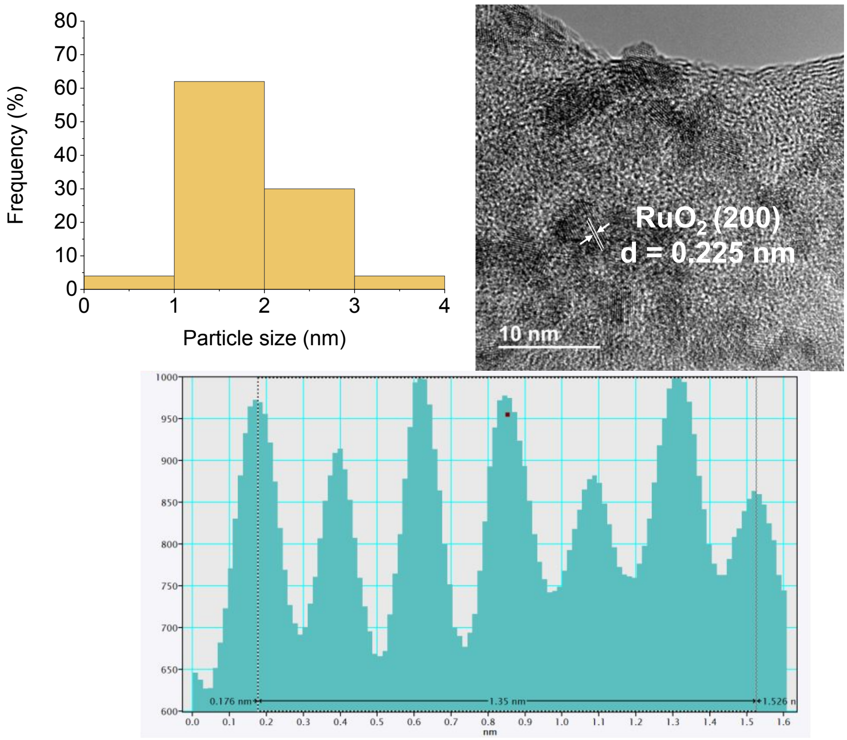


Figure S40. TEM images of used $RuO_x$(2 wt%)@MIL-101(Cr), $RuO_x$ NPs size distribution and interplane distance measurement. Average particle size is 1.89 ± 0.50 nm.

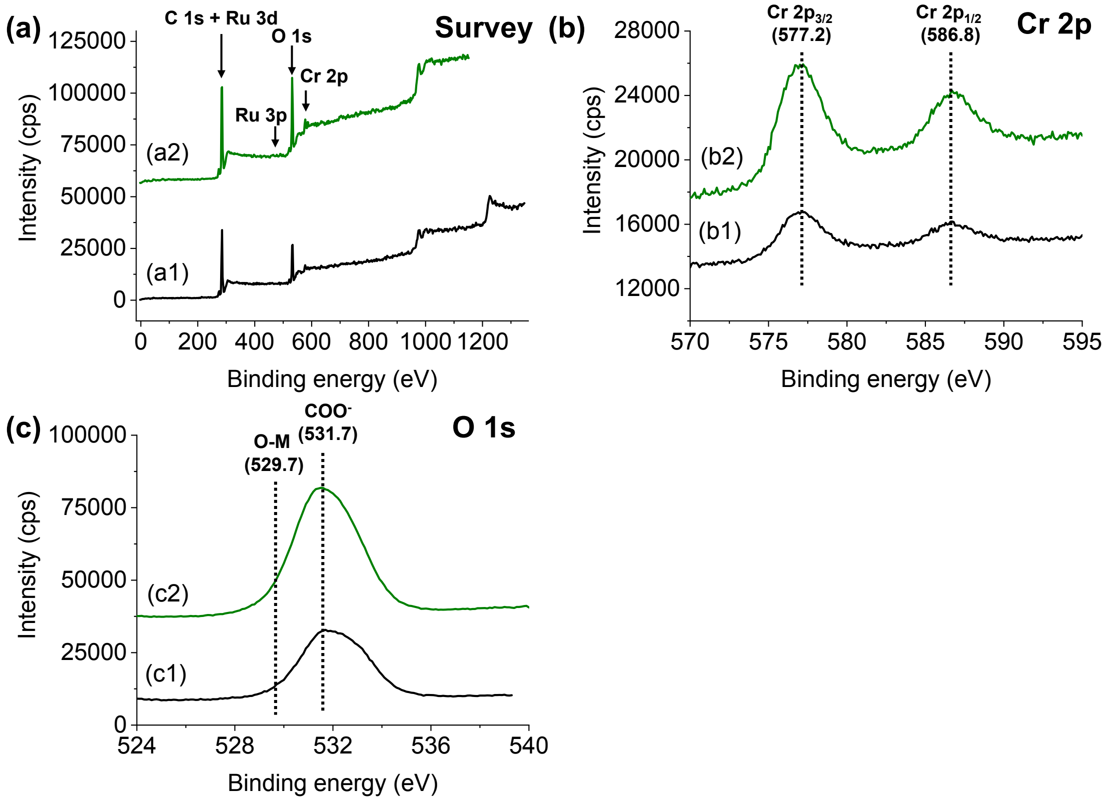


Figure S41. XPS comparison between fresh (black: a1, b1 and c1) and used 5 times (green: a2, b2 and c2) of $RuO_x$(2 wt%)@MIL-101(Cr).

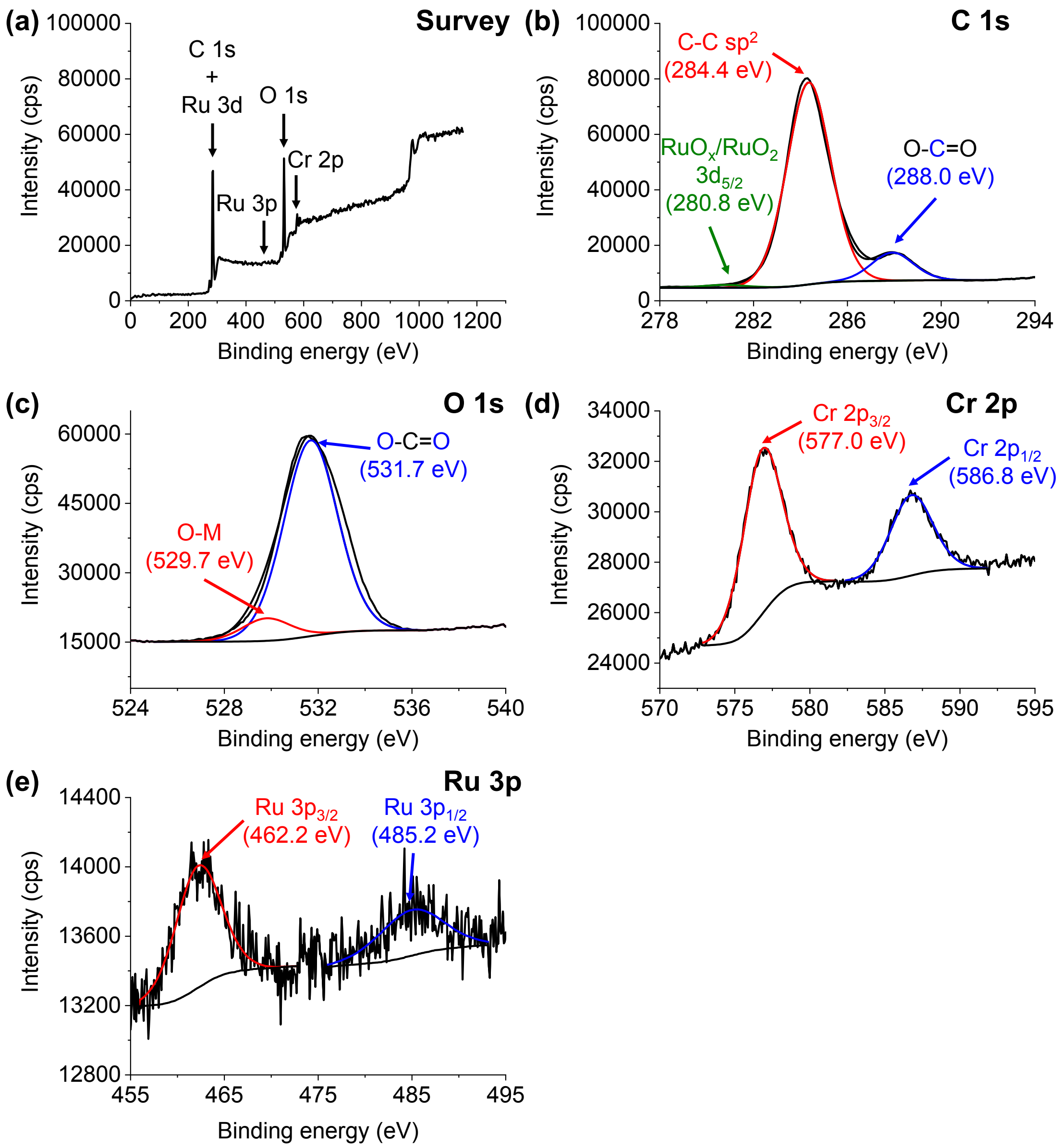


Figure S42. (a) XPS survey, (b) C 1s, (c) O 1s, (d) Cr 2p and (e) Ru 3p of five times used $RuO_x$(2 wt%)@MIL-101(Cr).

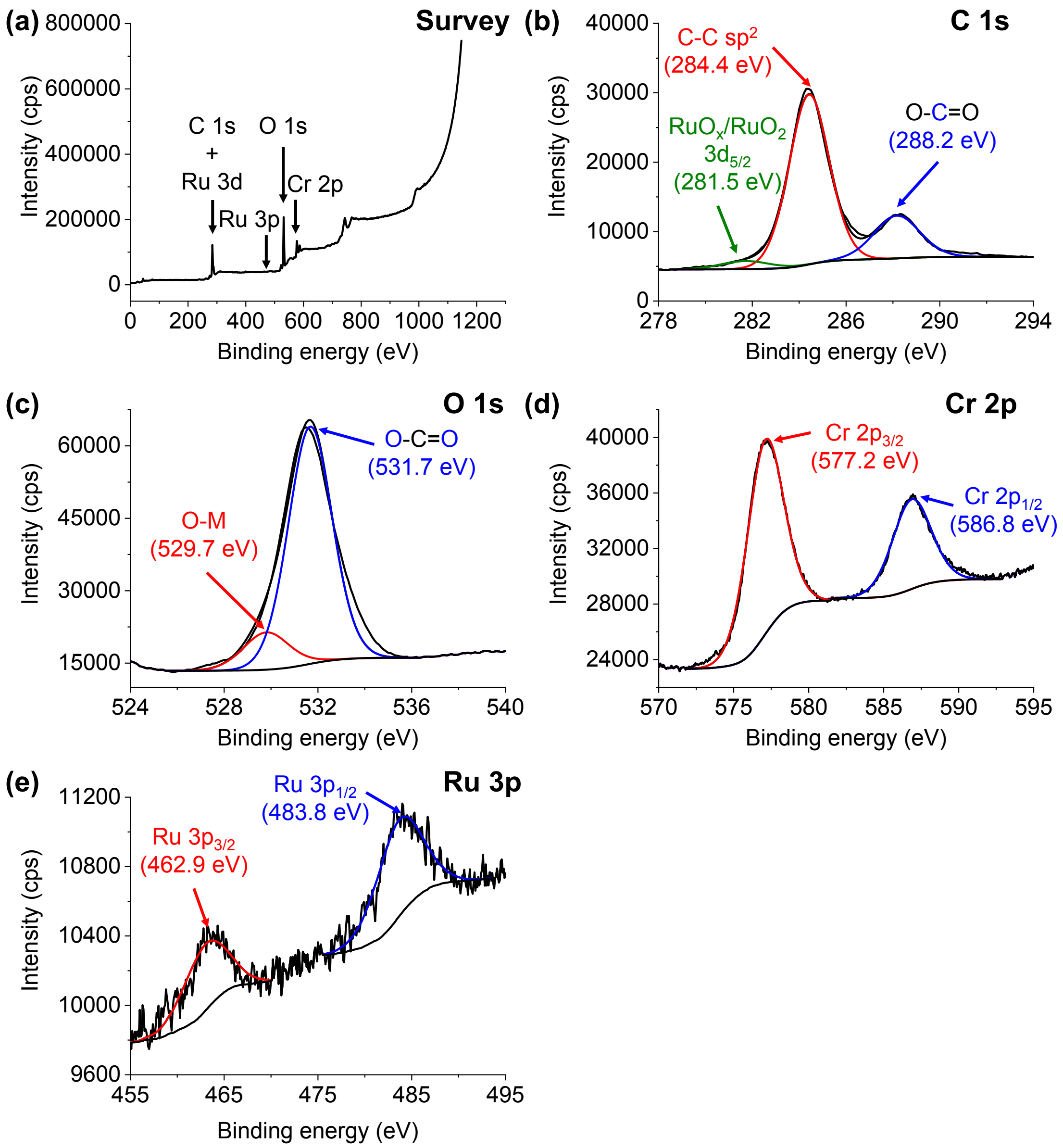


Figure S43. (a) XPS survey, (b) C 1s, (c) O 1s, (d) Cr 2p and (e) Ru 3p of the fresh $RuO_x$(2 wt%)@MIL-101(Cr). Note: For comparison, these XPS data have been performed under identical conditions as of Figure S42 and both are compared in Figure S43.

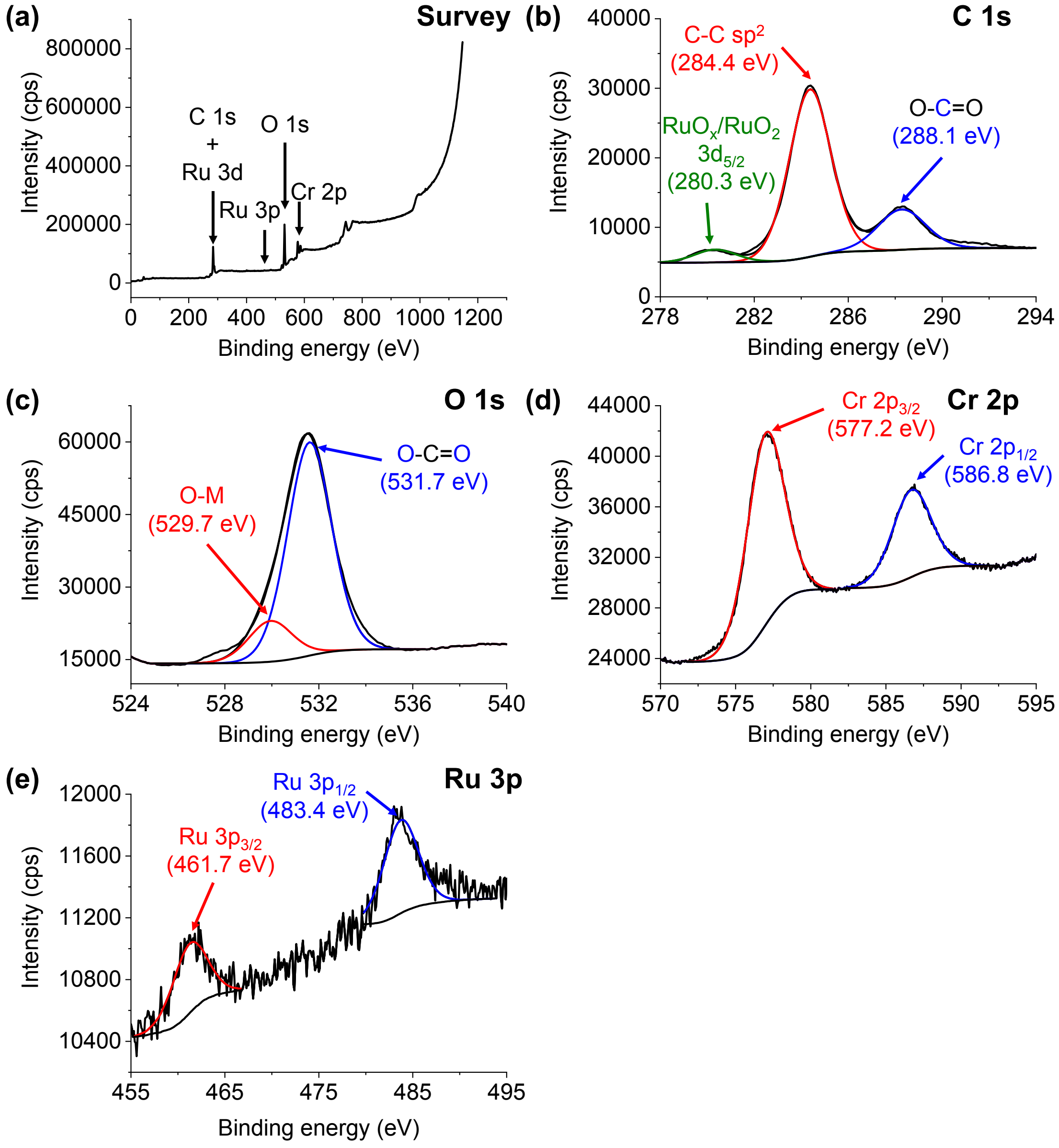


Figure S44. (a) XPS survey, (b) C 1s, (c) O 1s, (d) Cr 2p and (e) Ru 3p of the $RuO_x$(2 wt%)@MIL-101(Cr) previously reduced with $H_2$ flow at 200 ºC for 1 h. Note: These XPS analyses were performed after measuring the XPS of fresh sample (Figure S41). Then the sample was hydrogenated *in situ* and subsequently measured again.

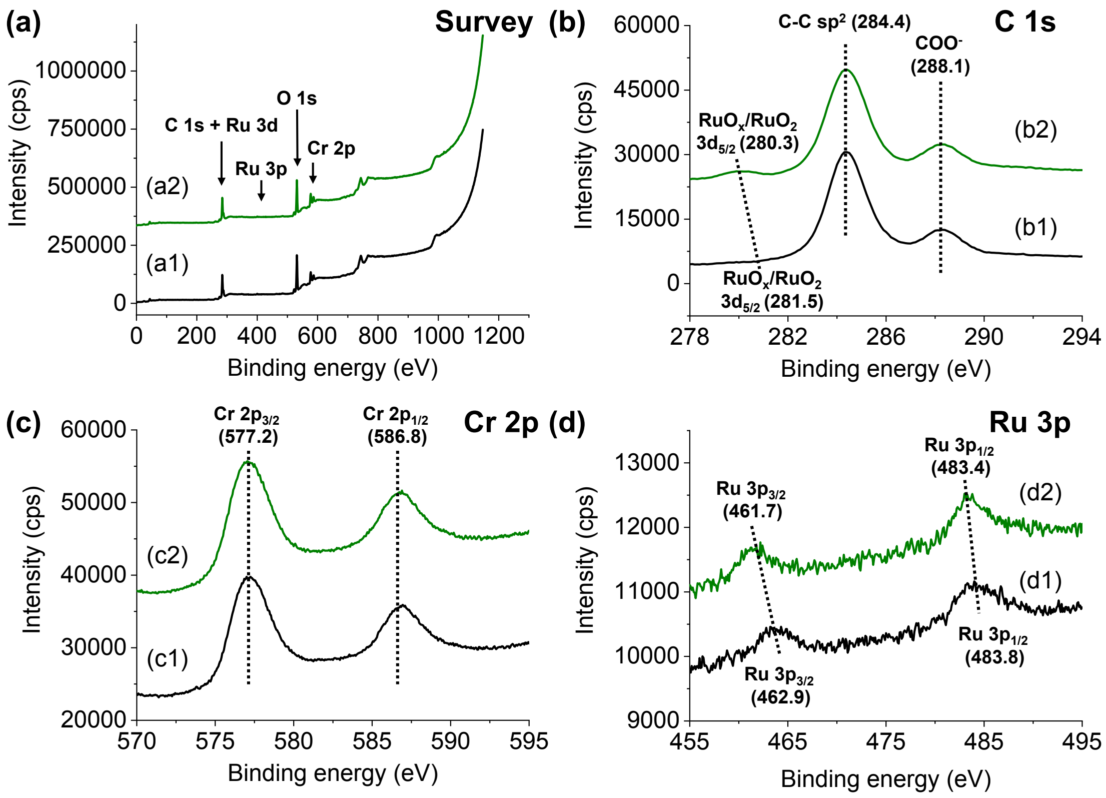


Figure S45. Comparison of XPS between fresh (a1, b1, c1, d1) and hydrogenated (a2, b2, c2, d2) $RuO_x$(2 wt%)@MIL-101(Cr) solids.

| Table S3. Parameters from Randles circuit employed to fit the Nyquist plots of used RuOx@MIL-101(Cr) solids. | | | |
|---|---|---|---|
| **Material** | **$R_p$ (ohm)** | **$R_u$(ohm)** | **$C_f$ (F)** |
| MIL-101(Cr) | 5450.0 ± 29.6 | 45.4 ± 0.1 | 1.3231e-04 ± 5.3156e-07 |
| Used $RuO_x$(0.2 wt.%)@MIL-101(Cr) | 4392.7 ± 21.9 | 13.8 ± 0.1 | 9.1764e-05 ± 3.2896e-07 |
| Used $RuO_x$(0.5 wt.%)@MIL-101(Cr) | 3746.1 ± 19.0 | 25.8 ± 0.1 | 1.1780e-04 ± 4.5602e-07 |
| Used $RuO_x$(1 wt.%)@MIL-101(Cr) | 3068.4 ± 14.8 | 13.0 ± 0.1 | 1.0393e-04 ± 3.8193e-07 |
| Used $RuO_x$(2 wt.%)@MIL-101(Cr) | 1971.5 ± 10.1 | 10.4 ± 0.1 | 9.5409e-05 ± 3.6019e-07 |
| Used $RuO_x$(2 wt.%)@MIL-101(Cr) after irradiation | 1550.6 ± 8.0 | 9.9 ± 0.1 | 1.0776e-04 ± 4.1537e-07 |

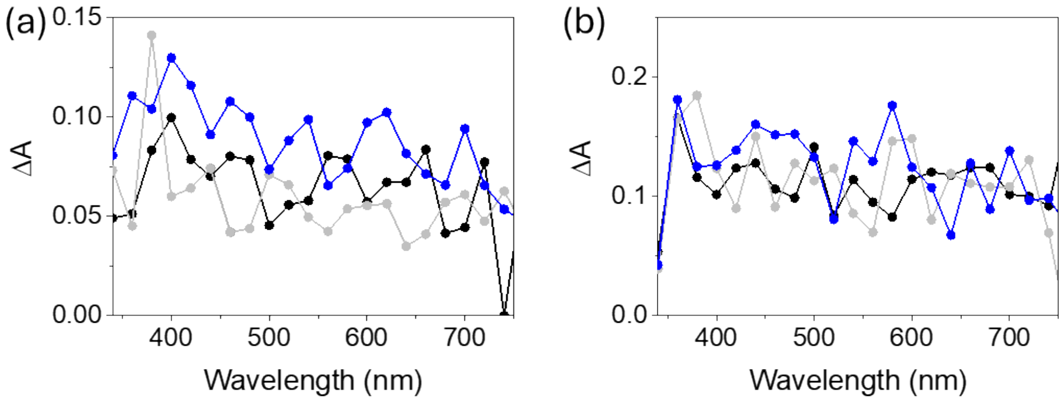


Figure S46. LFP spectra 0.02 μs after the laser pulse in Ar (black), $O_2$ (gray) and in the presence of MeOH (blue) for $RuO_x$(0.2 wt%)@MIL-101(Cr) (a) and pristine MIL-101(Cr) (b) in acetonitrile suspensions after excitation at 266 nm.

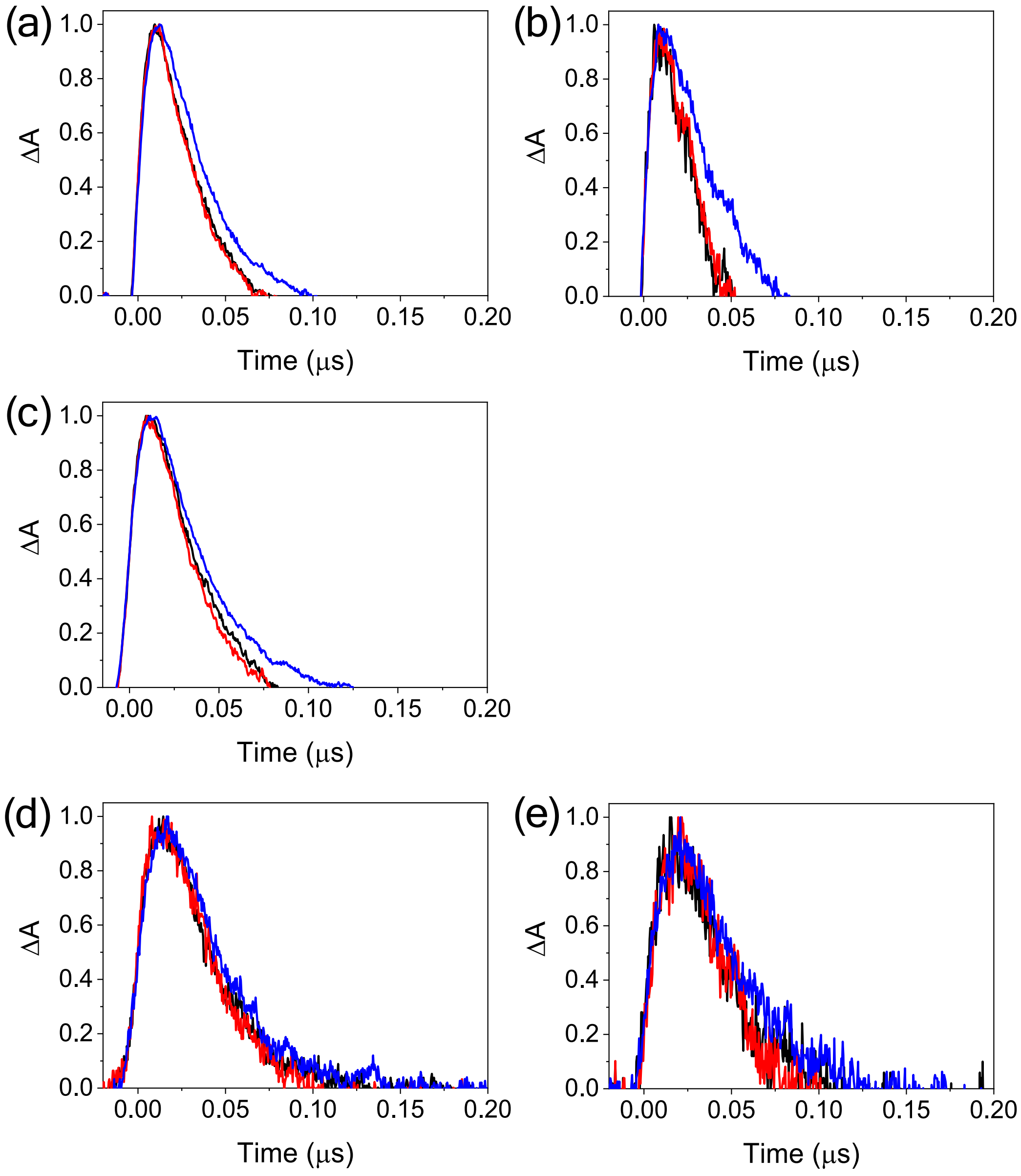


Figure S47. LFP decay traces in Ar (black), $O_2$ (red) and in the presence of MeOH (blue) for $RuO_x$(0.2 wt%)@MIL-101(Cr) at 350 nm (a), 450 nm (c) and 650 nm (d), and for $RuO_x$(2 wt%)@MIL-101(Cr) at 350 nm (b) and 650 nm (e). All measurements were performed at $\lambda_{exc}$ = 266 nm.

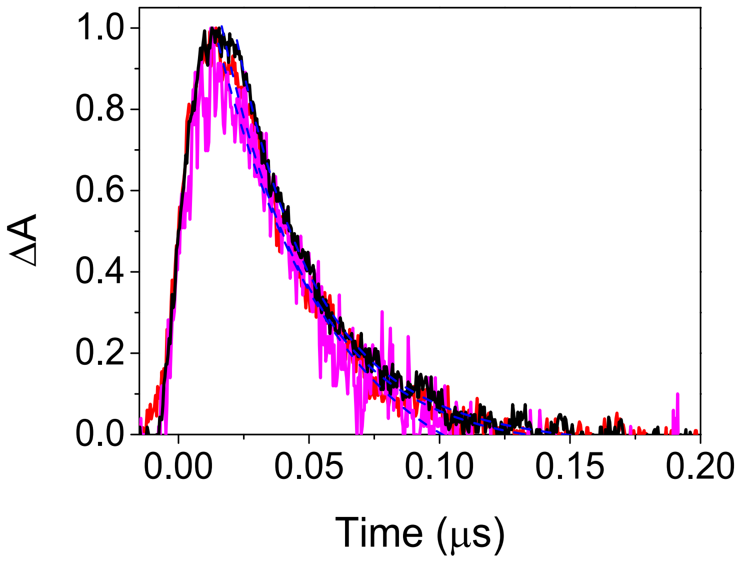


Figure S48. LFP decay traces for pristine MIL-101(Cr) (black), RuOx(0.2 wt%)@MIL-101(Cr) (red) and $RuO_x$(2 wt%)@MIL-101(Cr) (magenta) monitored at 650 nm under Ar. The fitted curves are shown in dashed blue.

Table S4. The lifetime and the percentage of the excited species present in MIL-101(Cr) and used $RuO_x$(2 wt%)@MIL-101(Cr).

| **MOF** | **Species** | **Lifetime (ns)** | **Percentage (%)** |
|---|---|---|---|
| MIL-101(Cr) | 1 | $\tau_1 = 0.381$ | 23.74 |
| | 2 | $\tau_2 = 1.688$ | 37.68 |
| | 3 | $\tau_3 = 12.05$ | 38.58 |
| Used $RuO_x$(2 wt%)@MIL-101(Cr) | 1 | $\tau_1 = 0.489$ | 26.33 |
| | 2 | $\tau_2 = 1.886$ | 37.91 |
| | 3 | $\tau_3 = 10.26$ | 35.75 |

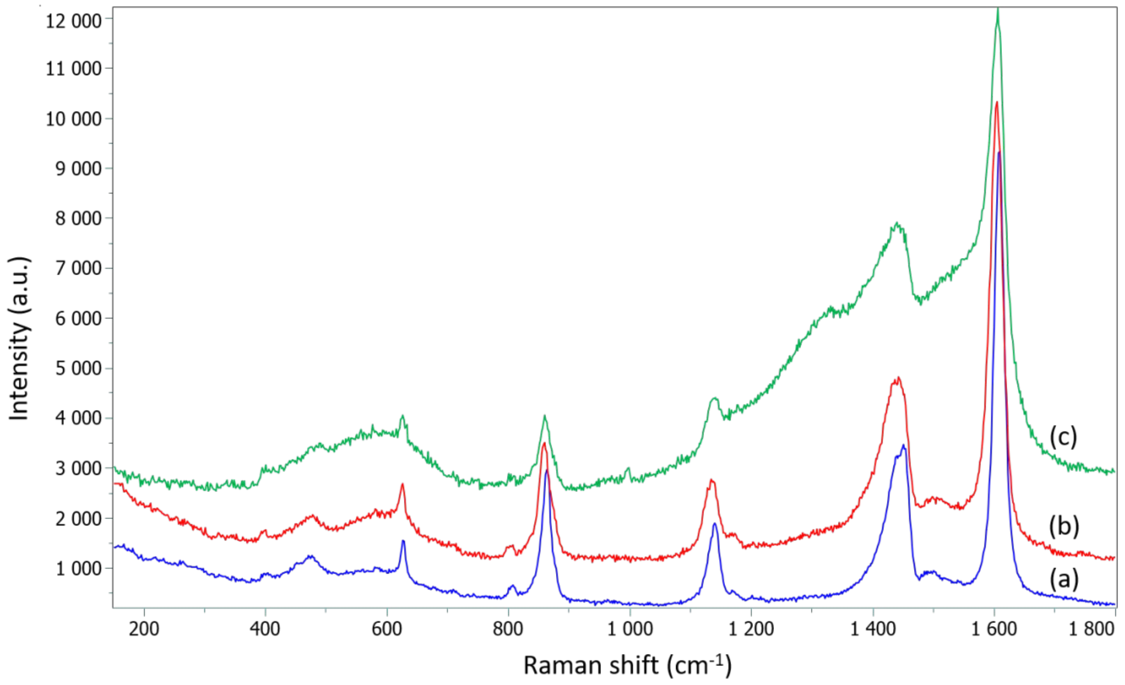


Figure S49. *Operando* Raman spectra of $RuO_x$(2 wt%)@MIL-101(Cr) (a) before activation , (b) after activation under $H_2$ at 200°C for 1h, and (c) after photocatalytic $CO_2$ methanation reaction at 200°C.

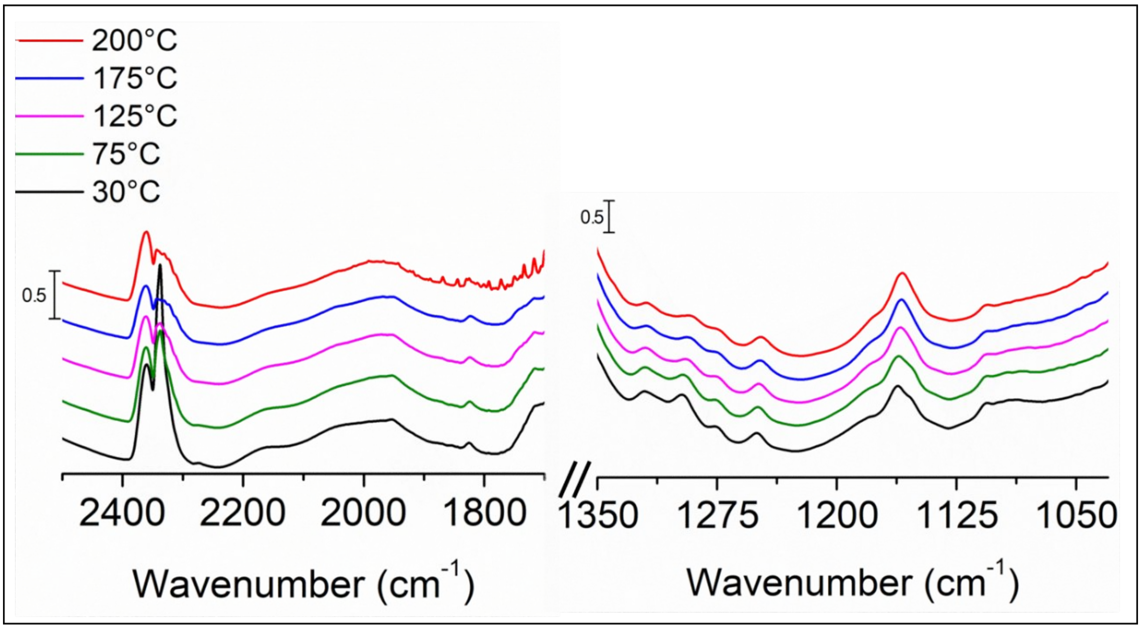


Figure S50. *Operando* direct FT-IR spectra of used RuOx(2wt%)@MIL-101(Cr) versus temperature: in the 2450-1800 $cm^{-1}$ and the 1200-1000 $cm^{-1}$ vibrational regions.

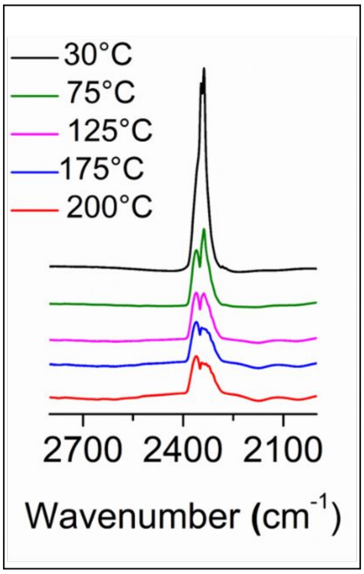


Figure S51. *Operando* FT-IR spectra of $CO_2$ adsorption on MIL-101(Cr) versus temperature.

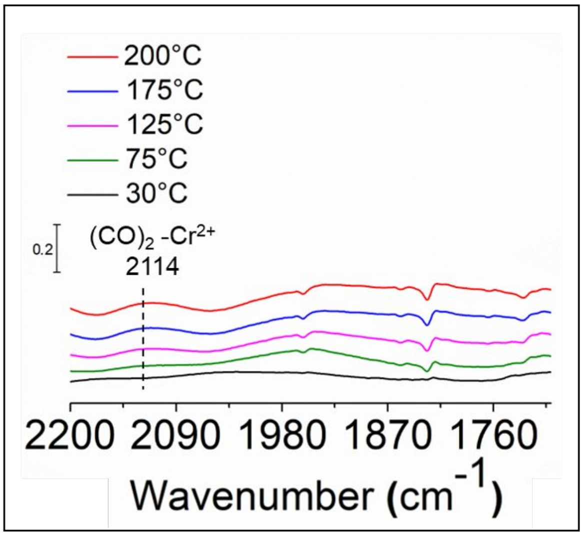


Figure S52. *Operando* FTIR spectra of MIL-101(Cr) versus temperature: in the CO region (2200-1800 $cm^{-1}$) and the 1200-1000 $cm^{-1}$ vibrational regions.

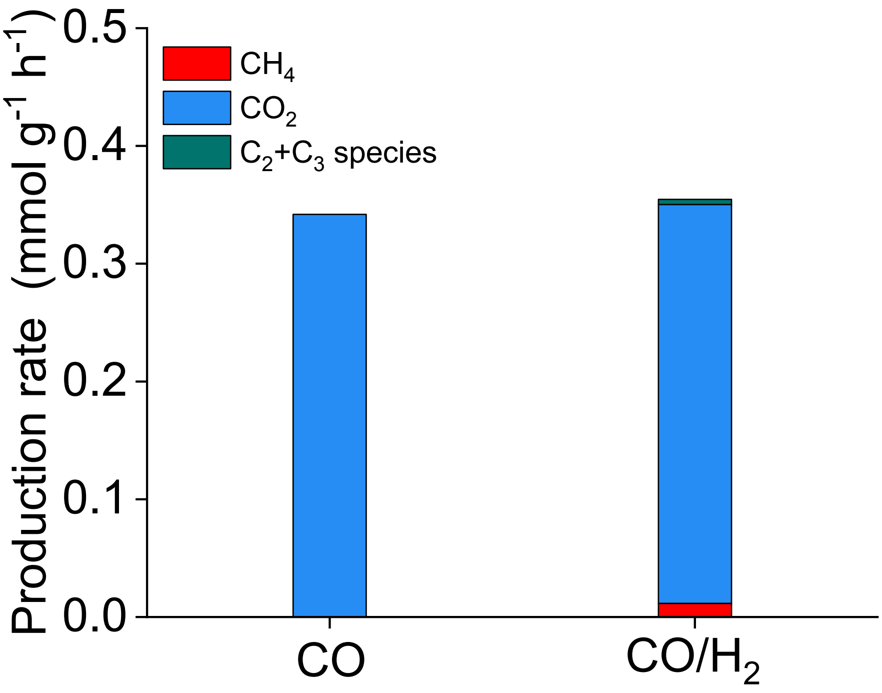


Figure S53. The activity of RuOx(2 wt%)@MIL-101(Cr) at 200 °C under visible light irradiation with CO or with a $H_2$:CO molar ratio of 3:1 with a total flow rate of 10 $cm^3 \cdot min^{-1}$.

| Table S5. Different vibrational modes of adsorbed CO over used $RuO_x$(2 wt%)@MIL-101(Cr) based on literature.[13, 14] | | | |
|---|---|---|---|
| Species | Frequencies ($cm^{-1}$) | Support | Reference |
| $Cr^{2+}(CO)_2$ | 2114 | $SiO_2$ | 15 |
| $Ru^{2+}(CO)_2$ | 2000 | $ZrO_2$ | 13 |
| $Ru^{\sigma+}(CO)$ | 1944 | $TiO_2$ | 15 |
| $Ru^0(CO)$ | 2045 | $SiO_2$ | 15 |

| Table S6. Vibrational modes of different adsorbed species on $RuO_x$(2 wt%)@MIL-101(Cr) over the $CO_2$ methanation reaction based on literature. [15-19] | | | | |
|---|---|---|---|---|
| Band position with $^{12}CO_2$ ($cm^{-1}$) | Band position with $^{13}CO_2$ ($cm^{-1}$) | Δ( $cm^{-1}$) | Possible Assignment | Ref. |
| 1343 | 1339 | 4 | C-H bend (Formate) | 15 |
| 1306 | 1303 | 3 | C-H Torsional ($CH_4$) | 18 |
| 1287 | 1283 | 4 | O-CH stretch. (formate) | 15 |
| 1172 | 1167 | 4 | H-CO bend. (formyl) | 16 |
| 1147 | 1143 | 3 | rocking of methoxys | 17 |

**References**


(1) Férey, G.; Mellot-Draznieks, C.; Serre, C.; Millange, F.; Dutour, J.; Surblé, S.; Margiolaki, I. A chromium terephthalate-based solid with unusually large pore volumes and surface area. *Science* **2005**, *23*, 2040-2042.

(2) Yang, M.; Tang, J.; Ma, Q.; Zheng, N.; Tan, L. High activity Fe-MIL-101 solid acid catalyst for acetalization of aldehydes with methanol and enamination of β-dicarbonyl compounds. *J. Porous Mater.* **2015**, *22*, 1345−1350.

(3) Rueda-Navarro, C. M.; Abou Khalil, Z.; Melillo, A.; Ferrer, B.; Montero, R.; Longarte, A.; Daturi, M.; Vayá, I.; El-Roz, M.; Martínez-Martínez, V.; et al. Solar gas-phase CO2 hydrogenation by multifunctional UiO-66 photocatalysts. *ACS Catal.* **2024**, *14*, 6470-6487.

(4) Rueda-Navarro, C. M.; Cabrero-Antonino, M.; Escamilla, P.; Díez-Cabanes, V.; Fan, D.; Atienzar, P.; B.;, F.; Vayá, I.; Maurin, G.; Baldoví, H. G.; et al. Solar-assisted photocatalytic water splitting using defective UiO-66 solids from modulated synthesis. *Nano Res.* **2024**, *17*, 4134–4150.

(5) Dankar, J.; Rouchon, V.; Rivallan, M.; Pagis, C.; El-Roz, M. Evidence on C–C Coupling to Acetate as Key Reaction Intermediate in Photocatalytic Reduction of CO2 over Pt/TiO2. *ACS Appl. Mater. Interf.* **2024**, *32*, 42210–42220.

(6) Hamoud, H. I.; Wolski, L.; Abdelli, H.; Chtourou, R.; Lebedev, O.; Martin, C.; Fan, D.; Maurin, G.; Maignan, A.; El-Roz, M. Earth-Abundant-Based Photocatalysts for Efficient and Selective H2 Production through Reforming of Formic Acid under Visible Light. *ACS Catal.* **2023**, *13*, 16266–16278.

(7) Wolski, L.; El-Roz, M.; Daturi, M.; Nowaczyk, G.; Ziolek, M. Insight into methanol photooxidation over mono- (Au, Cu) and bimetallic (AuCu) catalysts supported on niobium pentoxide — An operando-IR study. *Appl. Catal. B-Environ.* **2019**, *258*, 117978.

(8) Rueda-Navarro, C. M.; Cabrero-Antonino, M.; Escamilla, P.; Díez-Cabanes, V.; Fan, D.; Atienzar, P.; Ferrer B.; Vayá, I.; Maurin, G.; Baldoví, H. G.; et al. Solar-assisted photocatalytic water splitting using defective UiO-66 solids from modulated synthesis. *Nano Res.* **2024**, *17*, 4134–4150.

(9) Cabrero-Antonino, M.; Melillo, A.; Montero-Lanzuela, E.; Álvaro, M.; Ferrer, B.; Vayá, I.; Baldoví, H. G.; Navalón, S. Solar-driven gas phase photocatalytic CO2 methanation by multimetallic UiO-66 solids decorated with RuOx nanoparticles. *Chem. Eng. J.* **2023**, *468*, 143553.

(10) Wang, S.; Cabrero-Antonino, M.; Navalón, S.; Chen-chen Cao, C.-c.; Tissot, A.; Dovgaliuk, I.; Marrot, J.; Martineau-Corcos, C.; Yu, L.; Wang, H.; et al. A robust titanium isophthalate metal-organic framework for visible-light photocatalytic CO2 methanation. *Chem* **2020**, *6*, 3409-3427.
(11) Cabrero-Antonino, M.; Remiro-Buenamañana, S.; Souto, M.; García-Valdivia, A. A.; Choquesillo-Lazarte, D.; Navalón, S.; Rodríguez-Diéguez, A.; Mínguez-Espallargas, G.; García, H. Design of cost-efficient and photocatalytically active Zn-based MOFs decorated with Cu2O nanoparticles for CO2 methanation. *Chem. Commun.* **2019**, *55*, 10932-10935.
(12) Cabrero-Antonino, M.; Ferrer, B.; Baldoví, H. G.; Navalón, S. Toward solar-driven photocatalytic CO2 methanation under continuous flow operation using benchmark MIL-125(Ti)-NH2 supported ruthenium nanoparticles. *Chem. Eng. J.* **2022**, *445*, 136426.
(13) Guglielminotti, E.; Boccuzzi, F.; Manzoli, M.; Pinna, F.; Scarpa, M. Ru/ZrO2 Catalysts: I. O2, CO, and NO Adsorption and Reactivity. *J. Catal.* **2000**, *192* 149–157.
(14) Hadjiivanov, K.; Lavalley, J.-C.; Lamotte, J.; Maugé, F.; Saint-Just, J.; Che, M. FTIR study of CO interaction with Ru/TiO2Catalysts. *J. Catal.* **1998**, 415–425.
(15) Hadjiivanov, K.; Saint-Just, J.; Che, M.; Tatibouët, J.-M.; Lamotte, J.; Lavalley, J.-C. Preparation and characterization of multiple ion-exchanged Pt/TiO2 catalysts. *J. Chem. Soc., Faraday Trans.* **1994**, *90*, 2277-2281.
(16) Morgan Jr, G. A.; Sorescu, D. C.; Zubkov, T.; Yates Jr, J. T. The formation and stability of adsorbed formyl as a possible intermediate in Fischer− Tropsch chemistry on ruthenium. *J. Phys. Chem. B.* **2004**, *108*, 3614–3624.
(17) Howe, R. F.; Gratzel, M. EPR observation of trapped electrons in colloidal titanium dioxide. *J. Phys. Chem.* **1985**, *89*, 4495–4499.
(18) Solis-Garcia, A.; Zepeda, T. A.; Fierro-Gonzalez, J. C. Spectroscopic evidence of the simultaneous participation of rhodium carbonyls and surface formate species during the CO2 methanation catalyzed by ZrO2-supported Rh. *Appl. Catal. B-Environ.* **2022**, *304*, 120955.
(19) Su, C.; Yeh, J.-C.; Chen, C.-C.; Lin, J.-C.; Lin, J.-L. Study of adsorption and reactions of methyl iodide on TiO2. *J. Catal.* **2000**, *194*, 45-54.
